# Supplementary material for: A missense variant effect map for the human tumor-suppressor protein CHK2
Source: Am J Hum Genet. 2024 Dec 5;111(12):2675–92. doi: 10.1016/j.ajhg.2024.10.013 (PMC11639082; doi:10.1016/j.ajhg.2024.10.013)
Supplement: Document S1. Figures S1–S13 and Tables S1–S4 [file mmc1.pdf]

The American Journal of Human Genetics, Volume *111*

Supplemental information

A missense variant effect map

for the human tumor-suppressor protein CHK2

Marinella Gebbia, Daniel Zimmerman, Rosanna Jiang, Maria Nguyen, Jochen Weile, Roujia Li, Michelle Gavac, Nishka Kishore, Song Sun, Rick A. Boonen, Rayna Hamilton, Jennifer N. Dines, Alexander Wahl, Jason Reuter, Britt Johnson, Douglas M. Fowler, Fergus J. Couch, Haico van Attikum, and Frederick P. Roth

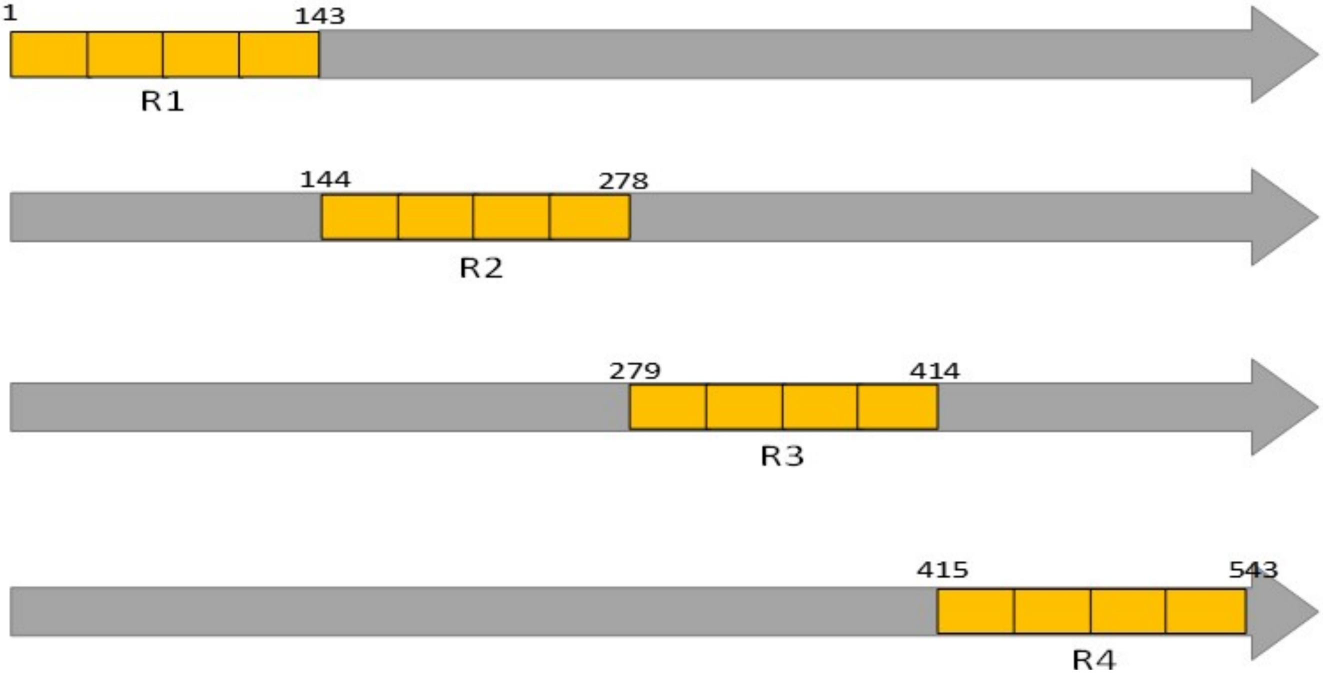

Figure S1. Mutagenized region of CHK2. We defined four regions of CHK2, corresponding to an average length of 150 AA each. Mutagenesis was targeted to each region in turn to generate four mutagenized libraries. For each region, we designed four sequencing tiles for the purpose of sequencing to estimate mutational frequencies before and after selection. The DMS-tileseq framework was followed separately for each regionally-mutagenized library.

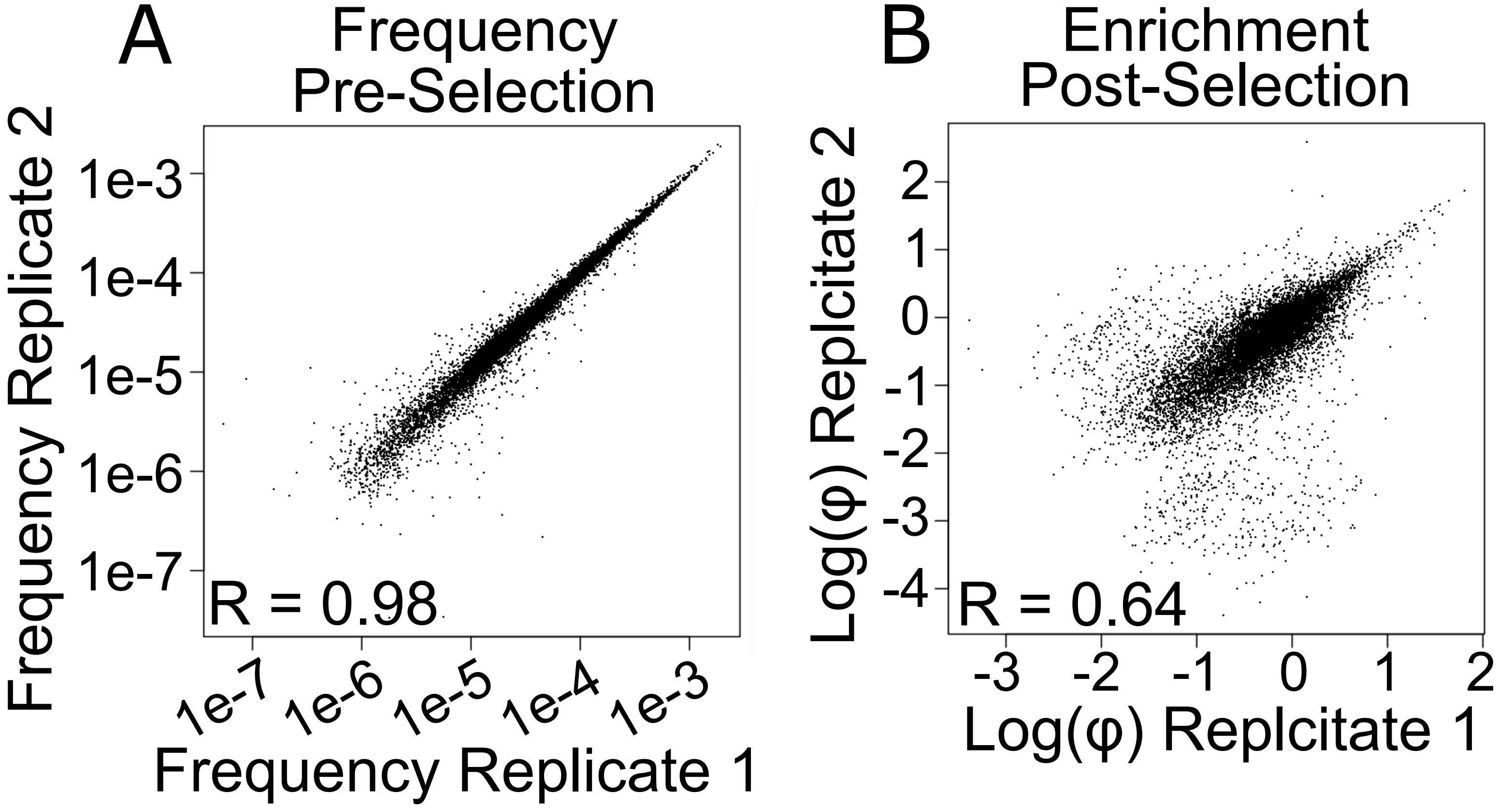

Figure S2. Replicate correlation across sequencing and selection. A To calculate the frequency of each CHEK2 variant in the pool and estimate error rates associated with tiling PCR and sequencing, two independent sequencing libraries from the non-select condition (baseline media without MMS) were prepared (see "quantifying variant abundance" section in Methods). Correlation of variant frequency between replicate pools was assessed by Pearson correlation. **B** Two independent experimental replicates from the selective condition (media with 0.007%MMS) were performed and correlation of variant-specific log(φ) enrichment ratios across replicates (comparing the frequency of each variant in the non-select condition to the select condition) was assessed by Pearson correlation.

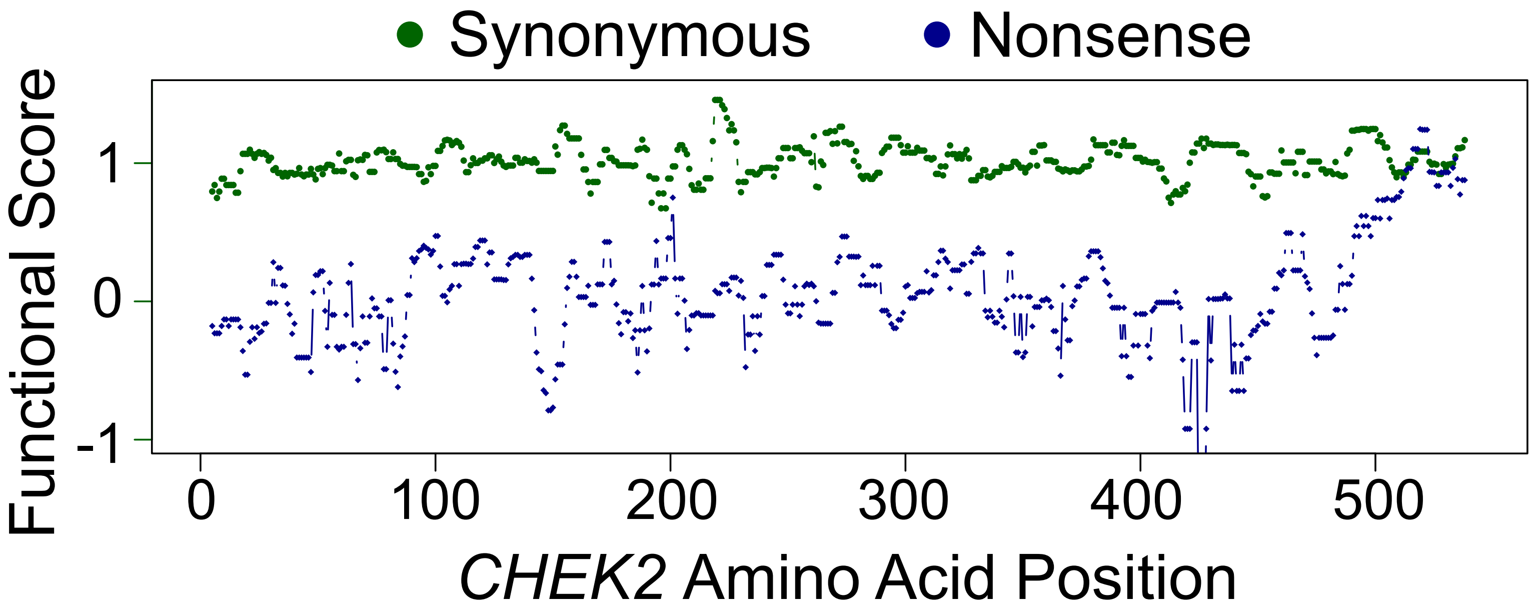

Figure S3. CHEK2 MMS assay effectively separates synonymous and nonsense variants. A moving window analysis of synonymous and nonsense scores along CHK2 positions was performed to assess the experiment's ability to separate neutral and loss-of-function variants. For each position evaluated, a window of 10 residues centered on that position was captured and median synonymous (green) and median nonsense (blue) scores were plotted. The majority of scores ranged from -1 to 1 with the exception of positions 425 to 427 for nonsense variants where scores dropped to between -2 to -3 (not shown).

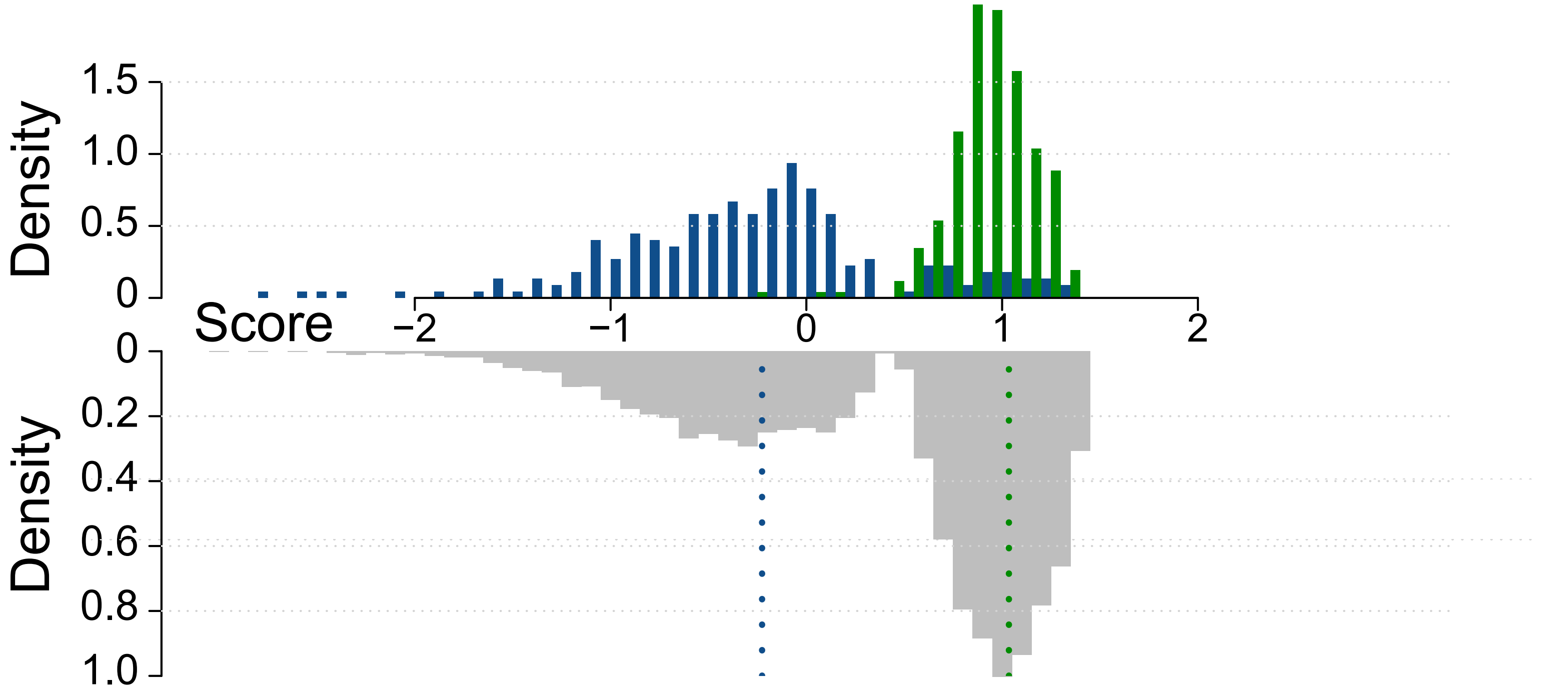

Figure S4. Distribution of CHK2 scores after confidence interval filtering and removal of hyper-complementing variants. Functional scores for synonymous, nonsense, and missense variants from the original map were plotted as a histogram with scores on the x-axis and density on the y-axis. Synonymous variants are shown in green, nonsense in blue, and missense in grey. The median value for each variant type is shown as dotted vertical lines that match the colour code above. Confidence interval filtering and removal of hypercomplementing variants as applied to the original functional as described in the methods.

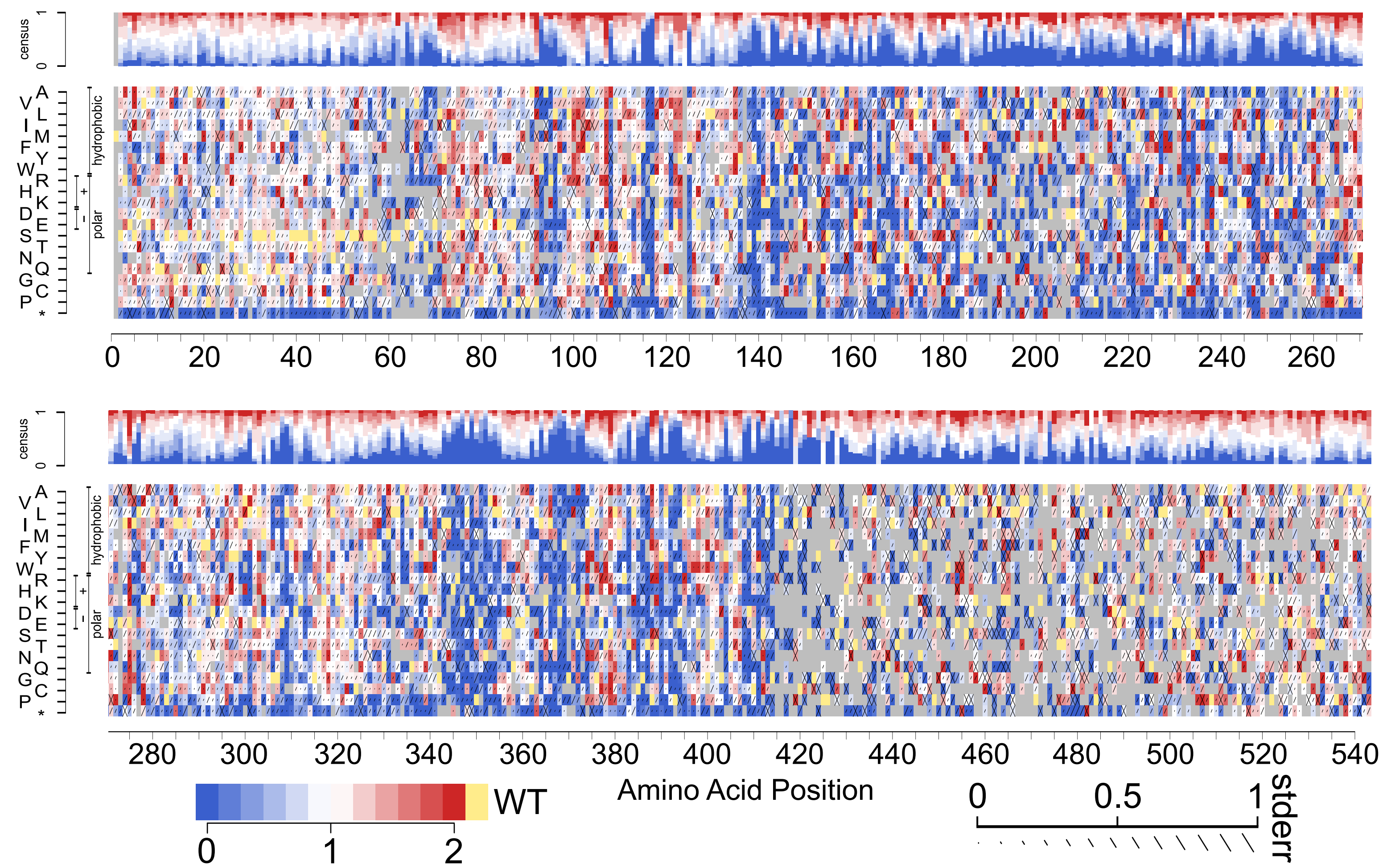

Figure S5. Full length CHK2 variant effect map.

Functional scores derived directly from the original assays are shown as a heatmap with positions along CHK2 on the x-axis and amino acid substitutions on the y-axis. Blue indicates deleterious variants with scores near 0, white represents tolerated variants with scores near 1, red indicates apparently 'hyper-complementing' variants with scores above 1, yellow indicates the canonical wild-type amino acid, and grey indicates missing data. Within each cell, the estimated standard error is indicated by the total length of lines as described in the legend (e.g., 0 error is indicated with a dot, error of 1 is indicated by a full-length slash, and an error higher than 1 is indicated by an X). The census track along the top of the plot depicts the distribution of scores at each position.

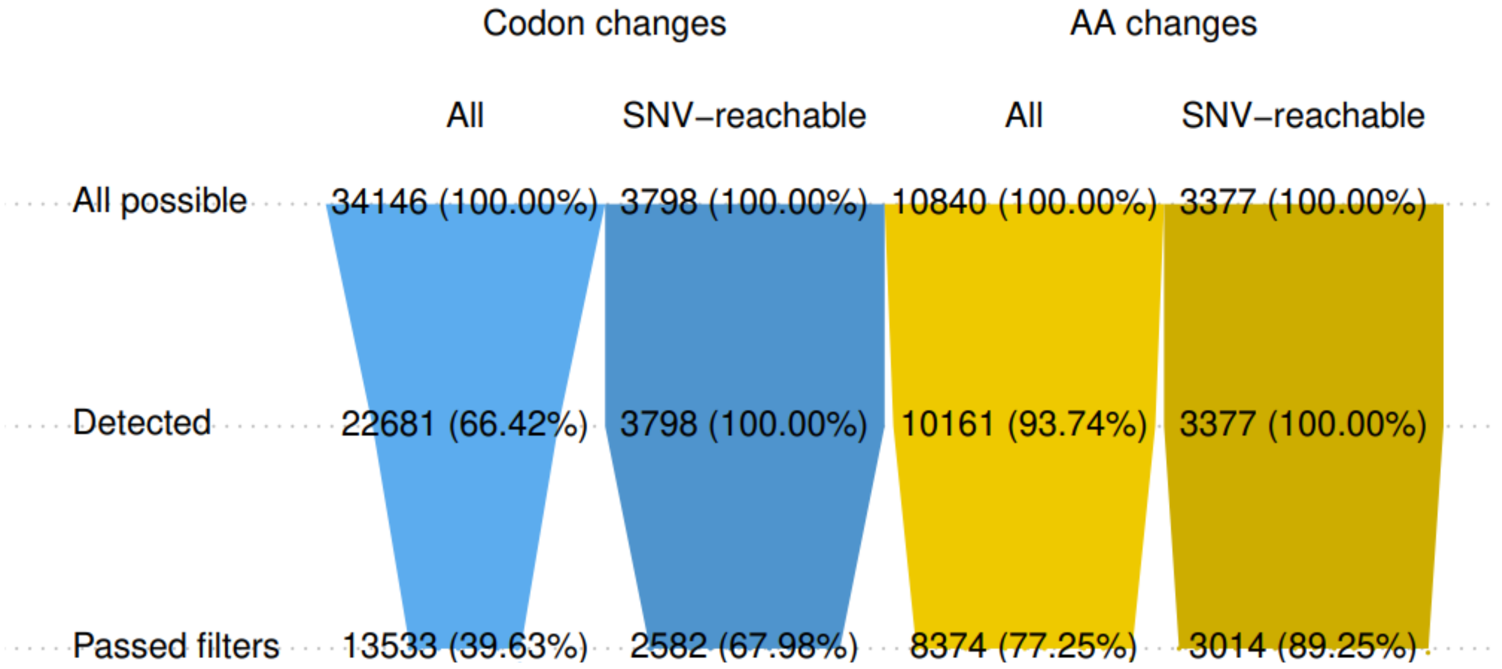

Figure S6. Coverage of all possible codons and amino acids in the CHK2 variant effect map.
The proportion of variants in CHEK2 covered by our experiment is indicated, either as: a fraction of all possible codon-level substitutions (lighter blue), a fraction of codon-level substitutions that can be reached via a single nucleotide change (darker blue); a fraction of all possible amino acid changes (lighter yellow); and a fraction of all possible amino acid changes that can be reached via a single-nucleotide change (darker yellow). “Detected” indicates variants that were observed during sequencing while “passed filters” refers to variants that were sufficiently well represented in the non-select library to be considered well-measured and were included in the final CHEK2 variant effect map.

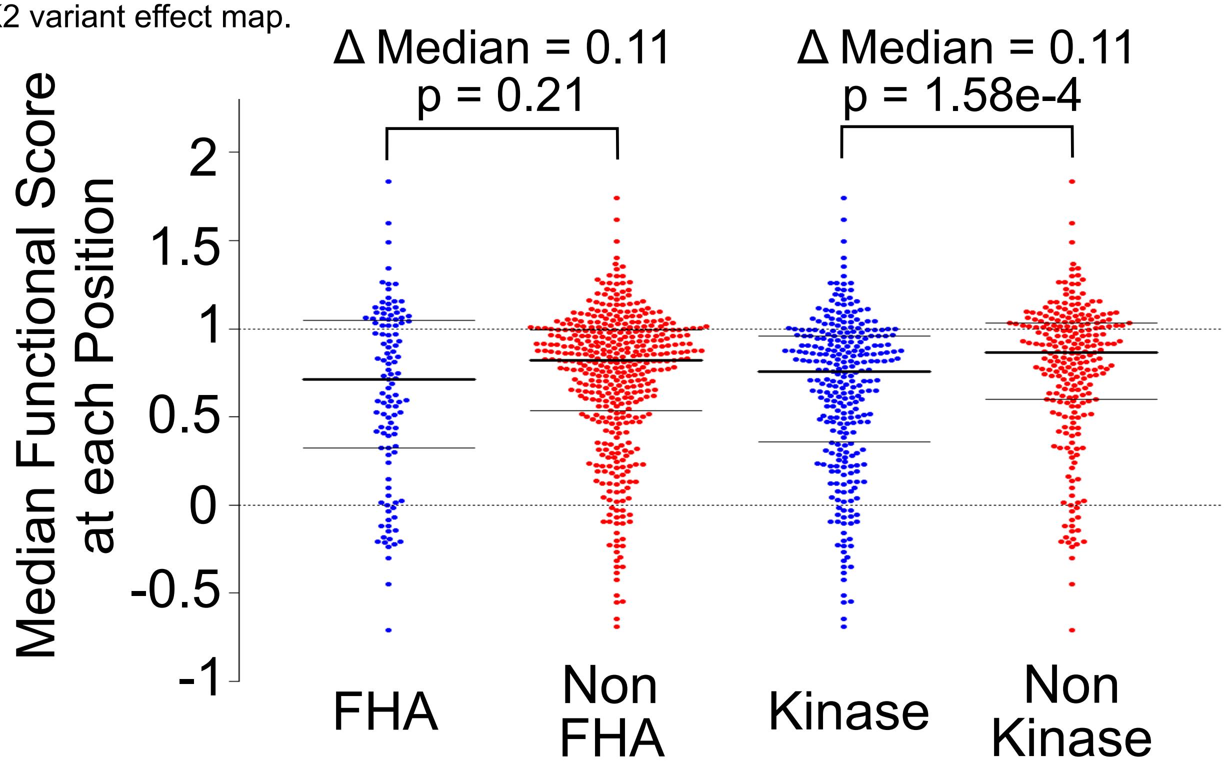

Figure S7. Comparing Functional scores in the FHA and kinase domain to the rest of CHK2.
The median functional score for missense variants at each position were stratified into those located in the FHA domain (92 to 205), the kinase domain (212 to 501), and those not located in the FHA domain (2 to 91 and 206 to 543) or not in the kinase domain (2 to 211 and 502 to 543). Positions located in the FHA domain were compared to those outside the FHA, as well as kinase domain positions compared to non-kinase domain, by Wilcoxon rank-sum test. The median value and 25th and 75th quantiles are overlaid on each distribution as solid horizontal lines. The dashed horizontal lines across all distributions indicate scores of zero and one, indicating deleterious and neutral variants respectively.

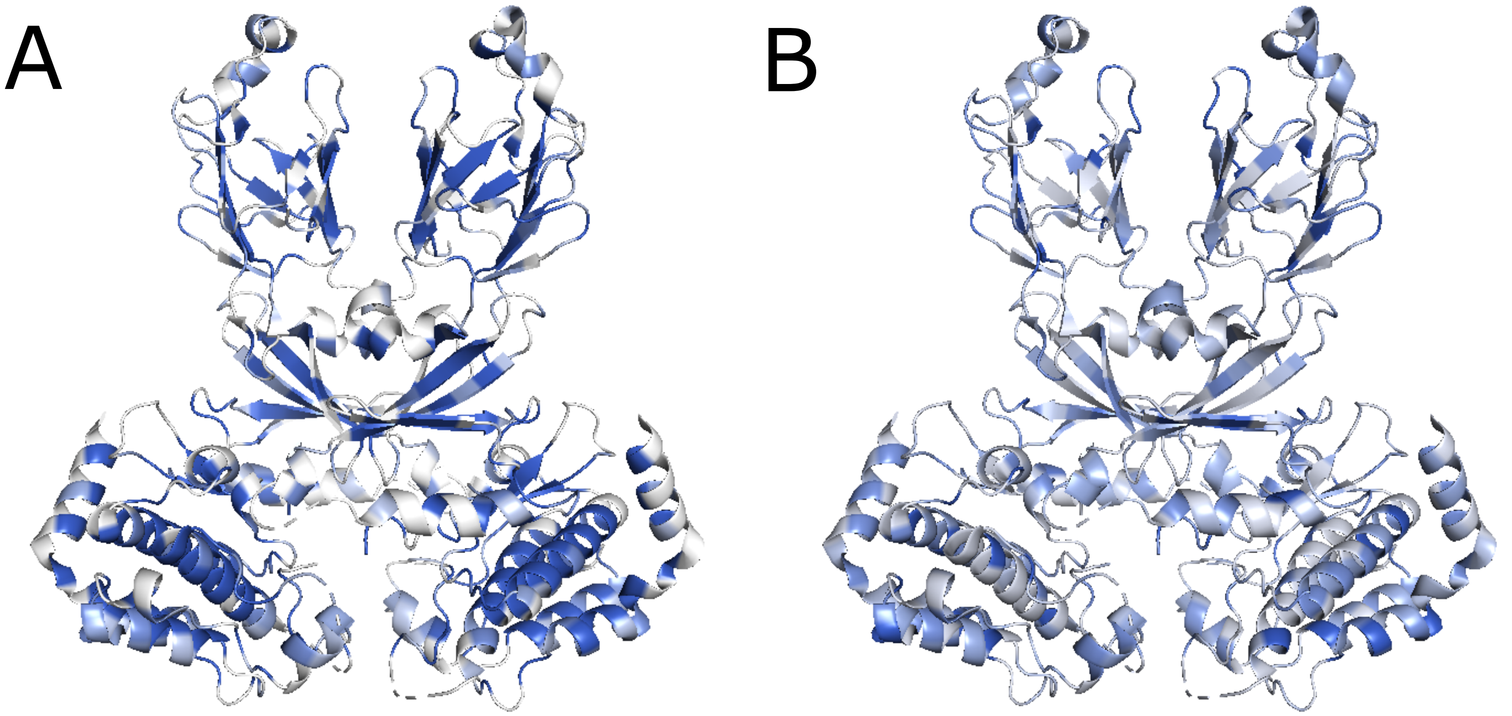

Figure S8. FoldX ΔΔG predicted stability effects overlaid on a homodimeric CHK2 crystal structure. **A** The median ΔΔG score for each position was calculated using FoldX and overlaid on the 3i6u CHK2 crystal structure. Darker blue colours indicate regions of CHK2 with destabilising ΔΔG scores (i.e. ΔΔG greater than 2), light blue indicates regions that were somewhat destabilising (i.e. ΔΔG less than 2 but greater than 0.5), and grey colouration indicates neutral effects i.e. ΔΔG less than 0.5 but greater than -0.5). **B** The difference in medians between FoldX-derived ΔΔG and our functional scores were overlaid on the 3i6u CHK2 crystal structure. FoldX and functional score were transformed to have a maximum value of 1 and minimum value of 0, with the absolute difference calculated. Regions that differed between ΔΔG and functional score are depicted in shades of blue, while areas coloured in white indicate agreement between ΔΔG and functional score (i.e. destabilising regions by ΔΔG are damaging by functional score, or stable regions of ΔΔG are tolerated according to functional score).

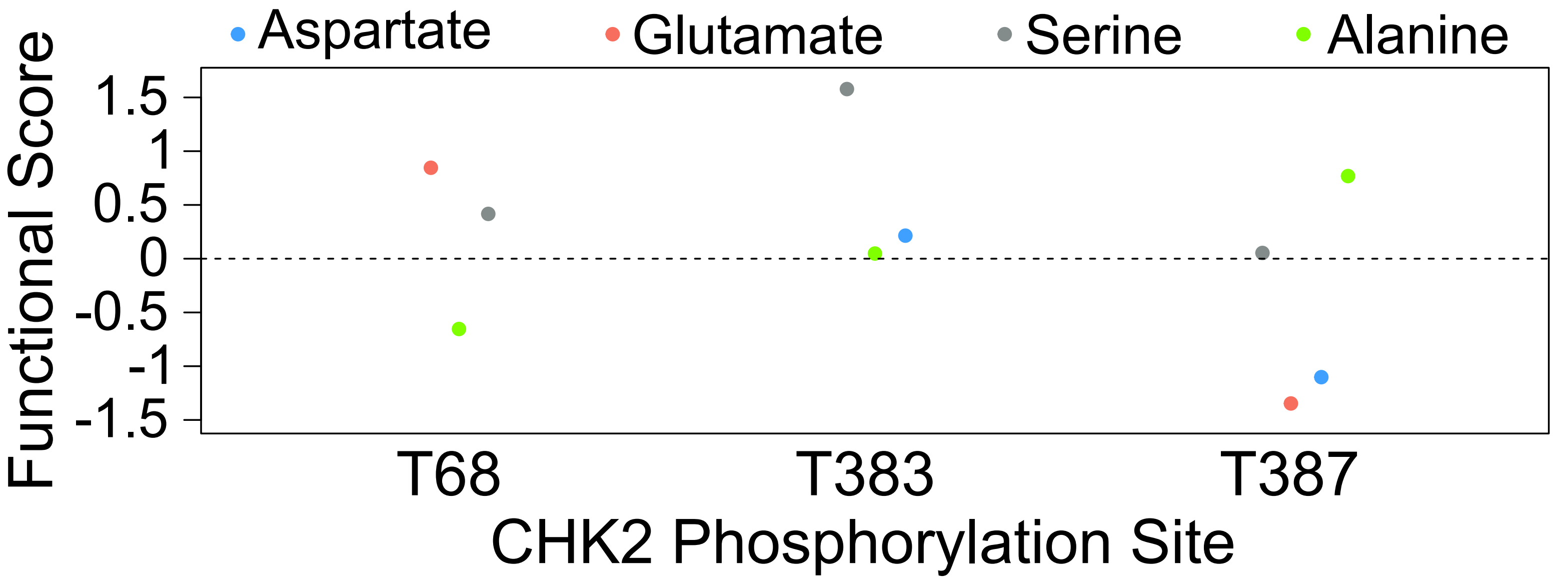

Figure S9. Effect of phosphomimetic and phosphodead variants at known CHK2 phosphorylation sites. Functional scores are shown for individual variants located at phosphorylation sites critical for CHK2 activation. Phosphomimetic mutations, aspartate and glutamate, are shown in blue and red respectively, the purportedly neutral variant (due to its ability to be phosphorylated) serine is shown in grey, and phosphodead alanine shown in green.

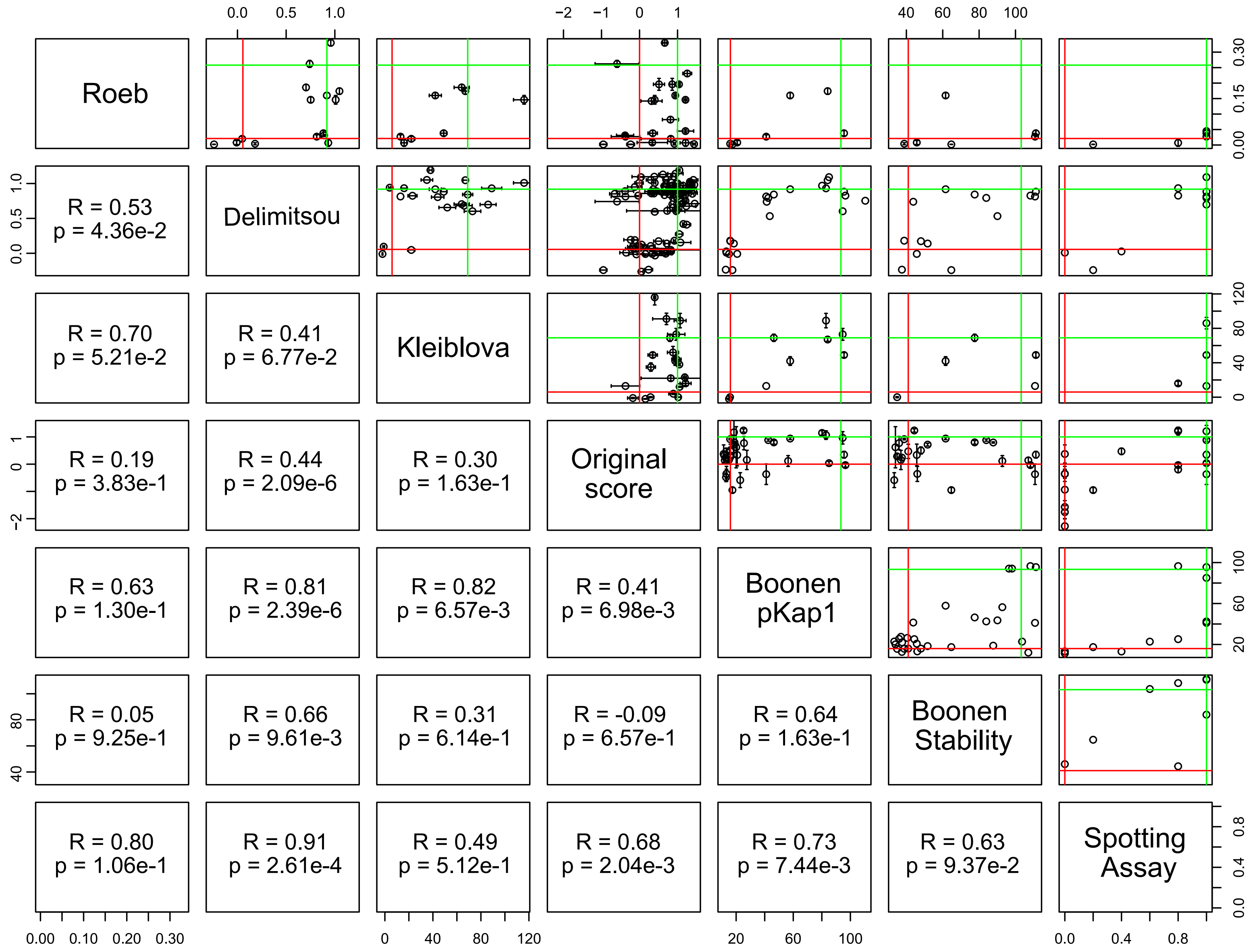

Figure S10. Scatterplots comparing all functional assays.
Scatterplots were generated comparing each of the functional assay datasets indicated on the diagonal axis. Starting from the top-left, this includes Roeb et al. 2012, Delimitsou et al. 2019, Kleiblova et al. 2019, our experimental functional scores, Boonen et al. 2022 pKAP1 and stability scores, and finally MMS spotting assays we performed for individual variants (data not shown). Correlation between datasets was assessed by Pearson correlation coefficient.

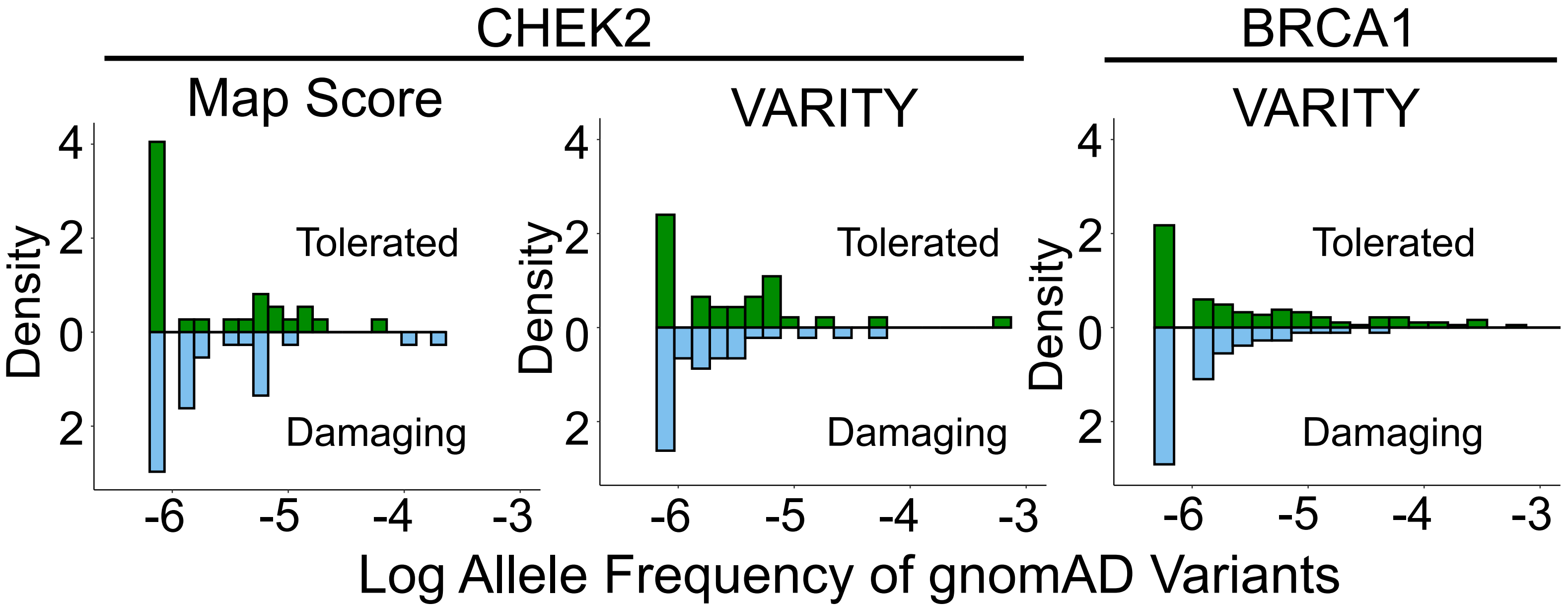

Figure S11. gnomAD allele frequencies for CHEK2 variants does not correlate with functional scores or VARITY. Allele frequencies for all missense variants in CHEK2 or BRCA1 were collected from gnomAD v4.1.0 and matched to either the variant effect map or to VARITY_R. Variants with map or VARITY_R scores in the bottom or top 5th-95th percentiles were isolated and their log10 allele frequencies plotted as a histogram. The top green half of the plot includes the variants expected to be tolerated by map score or VARITY_R, and the bottom blue half of the plot includes variants expected to be damaging variants by map score or VARITY_R.

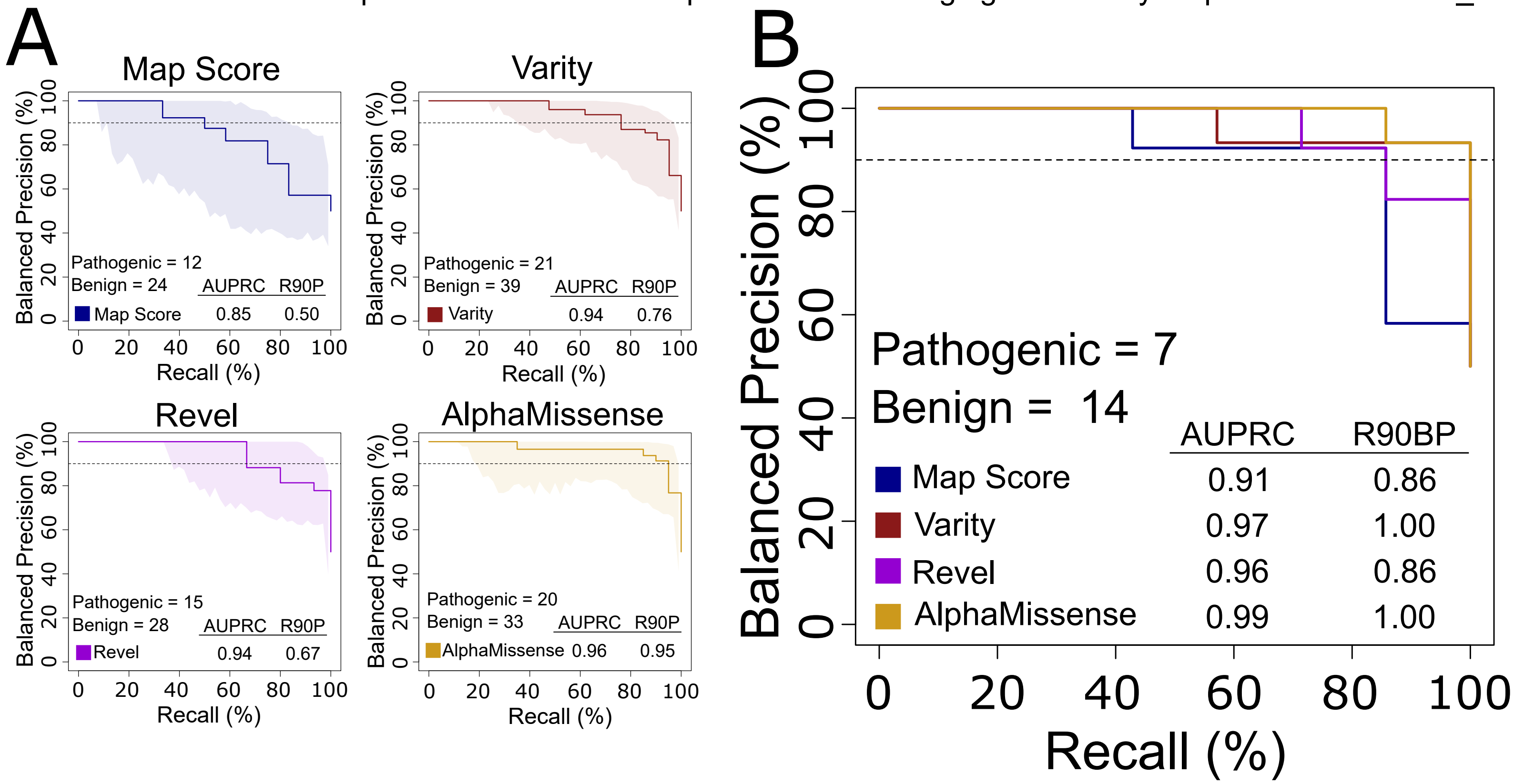

Figure S12. Balanced Precision-Recall Curves of computational and functional scores against variants with known pathogenic or benign annotations. A Using the direct original CHEK2 map scores and a known set of clinically annotated pathogenic or benign CHEK2 variants from Invitae, we evaluated balanced precision—defined at each score threshold by the fraction of variants that are pathogenic given a balanced (50% prior probability of pathogenicity) test set—versus recall (fraction of pathogenic variants captured at this threshold). The horizontal dashed line indicates Recall at 90% Balanced Precision (R90BP) with the numerical Area Under the Precision Recall Curve (AUPRC) and R90BP listed in the bottom-left hand legend. Individual PRCs are shown for functional scores (blue), VARITY_R (red), REVEL (purple), and AlphaMissense (Gold). **B** PRCs were generated for an overlapping set of variants that had scores in all three computational predictors (VARITY_R, REVEL, and AlphaMissense) as well as functional scores in the CHEK2 map.

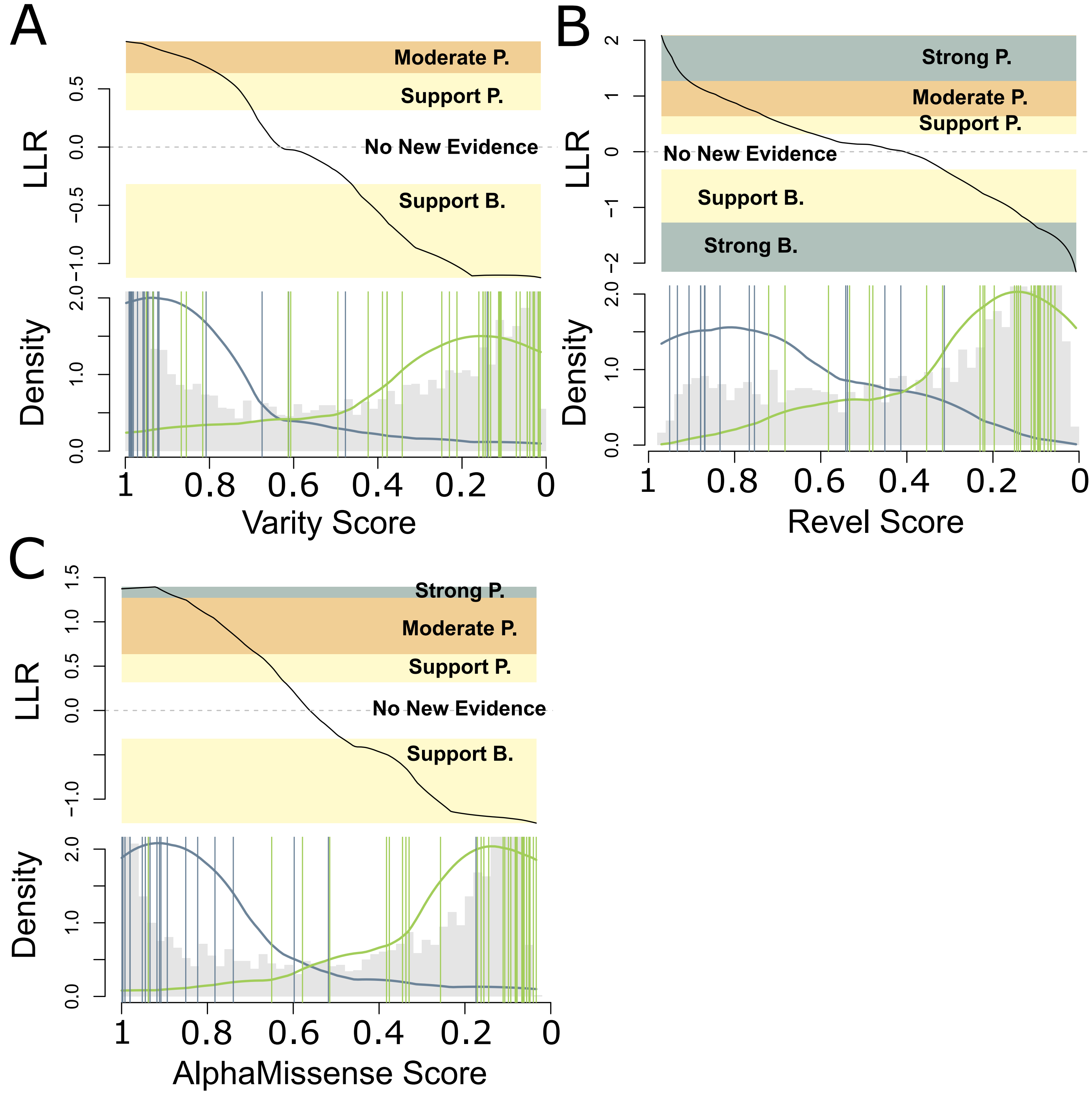

Figure S13. CHEK2 Log-Likelihood Ratios for computational predictors Varity, Revel, and AlphaMissense. LLRs of pathogenicity were calculated as described in Figure 8 panel C and in the methods. The log ratio between likelihood of observing a score in the positive pathogenic reference set (blue) compared to the negative benign reference set (green) was calibrated to ACMG evidence strengths. Probability distributions are overlaid on the grey histogram of CHEK2 missense variant scores with the top panel showing which score ranges correspond to each ACMG evidence strength. Panel **A** shows Varity_R, panel **B** Revel, and panel **C** AlphaMissense.

Region	Amino Acid (AA) Changes Per Clone	Fraction of all possible AA changes that are well measured	Fraction of all possible 1 nt-accessible AA changes that are well measured
1	0.51	86%	99%
2	0.17	81%	100%
3	0.37	92%	100%
4	0.22	62%	98%

Table S1. Fractions of amino acid substitutions that were sufficiently well represented in the “non-select” expression library (after transformation into yeast but not subjected to selection) to be considered well-measured. Here, we considered amino-acid substitutions to be well measured if at least 50 read counts were observed in the non-select library. Fraction of all possible codon changes considers missense and nonsense variants across all CHEK2 positions.

CHK2 amino acid position	Nucleotide and inhibitor binding sites	Median	Mean	Number of variants with score > 0.5	Number of variants with score < 0.5
**G227**	ADP	0.19	0.49	8	9
**S228**	ADP	0.50	0.49	8	8
**G229**	ADP	0.06	0.33	4	9
**G232**	ADP	-0.35	-0.38	0	16
**V234**	ADP and DBQ	-0.39	-0.13	4	13
**A247**	ADP and DBQ	-0.26	-0.26	2	14
**K249**	ADP and DBQ	-0.31	0.08	3	12
**L301**	DBQ	0.23	0.32	6	11
**E302**	ADP and DBQ	1.12	1.23	18	0
**L303**	ADP	1.20	1.37	15	0
**M304**	ADP and DBQ	0.70	0.65	10	7
**E308**	ADP and DBQ	0.04	-0.11	3	14
**E351**	ADP and DBQ	0.22	0.07	4	12
**N352**	ADP and DBQ	0.04	-0.27	0	18
**L354**	ADP and DBQ	-0.22	0.17	6	12
**D368**	ADP	-0.62	-0.38	1	16

Table S2. List of residues that had been previously identified as important for CHK2 activation on the basis of CHK2 crystal structures, based on proximity to either complexed ADP or the competitive ADP inhibitor debromohymenialdisine (DBQ). Columns of median and mean score and the number of variants above or below a score of 0.5 consider all well-measured missense variants at each position.

Hotspot	CHK2 amino acid position	Median	Mean	Number of variants with score > 0.5	Number of variants with score < 0.5
**APE**	392	-0.06	-0.02	5	14
	393	-0.76	-0.56	2	13
	394	-0.34	-0.28	2	12
**Salt bridge E**	273	0.43	0.41	1	2
**VAIK**	246	0.35	0.53	9	10
	247	-0.26	-0.26	2	14
	248	-0.05	0.25	8	10
	249	-0.31	0.08	3	12
**HRD**	345	-0.11	-0.27	2	14
	346	0.05	-0.04	2	15
	347	-0.55	-0.45	2	15
**GxGxxG**	227	0.19	0.49	8	9
	228	0.50	0.49	8	8
	229	0.06	0.33	4	9
	230	1.27	1.12	3	1
	231	1.12	0.98	13	5
	232	-0.35	-0.38	0	16
**DFG**	368	-0.62	-0.38	1	16
	369	-0.42	-0.57	0	18
	370	0.07	0.07	2	6
**HRD + 5**	352 (Asn)	0.04	-0.27	0	18
**APE – 6**	386 (Gly)	0.05	-0.03	5	14
**HRD – 6**	339 (His)	0.86	0.72	10	7
**HRD + 7**	354 (Leu)	-0.22	0.17	6	12
**HRD – 7**	338 (Leu)	0.56	0.49	7	7

Table S3. Mutational Hotspots Mutational hotspots identified in Hudson et al. 2018 were separated according to their correspondence to distinct kinase activation motifs. For each hotspot, the table indicates the amino acid position relative to the beginning (negative values) or end (positive values) of the nearest motif, as well as the median and mean of original functional scores at these positions. Of the 23 CHK2 hotspots identified, all 16 that fell within a named motif (APE, VAIK, HRD, DFG and the three G positions within the GxGxxG motif) scored as intolerant to variation in our map (i.e., with median scores below 0.5).

Tiling primer name	Tiling primer sequence
**CHEK2_1F**	TACACGACGCTCTTCCGATCTCAACTTTGTACAAAAAAGCAGGCTGCATG
**CHEK2_1R**	AGACGTGTGCTCTTCCGATCTGTAGAGGAGCTGGATATGCC
**CHEK2_2F**	TACACGACGCTCTTCCGATCTTCCTCCTCACAGTCCCAG
**CHEK2_2R**	AGACGTGTGCTCTTCCGATCTCAGGTTCTTGGTCCTCAGG
**CHEK2_3F**	TACACGACGCTCTTCCGATCTTGTCCACTCAGGAACTCTATTCTATT
**CHEK2_3R**	AGACGTGTGCTCTTCCGATCTCCAAACCAGTAGTTGTCATTCAC
**CHEK2_4F**	TACACGACGCTCTTCCGATCTGATGGATTTGCCAATCTTGAATGT
**CHEK2_4R**	AGACGTGTGCTCTTCCGATCTCCACTTCCCTGAAAATCCGAA
**CHEK2_5F**	TACACGACGCTCTTCCGATCTCCGAACATACAGCAAGAAACAC
**CHEK2_5R**	AGACGTGTGCTCTTCCGATCTATTGTTATTCAAAGGACGGCG
**CHEK2_6F**	TACACGACGCTCTTCCGATCTCAGAGCTTGTAGGGAAAGGAAAA
**CHEK2_6R**	AGACGTGTGCTCTTCCGATCTATGATGTATTCATCTCTTAATGCCTTAG
**CHEK2_7F**	TACACGACGCTCTTCCGATCTCTGACTGTAGATGATCAGTCAGTTTAT
**CHEK2_7R**	AGACGTGTGCTCTTCCGATCTTTTTGCTGATGATCTTTATGGCTACT
**CHEK2_8F**	TACACGACGCTCTTCCGATCTGCTTTCGAGAGGAAAACATGTAAG
**CHEK2_8R**	AGACGTGTGCTCTTCCGATCTTGATGATGCAAGGATGATTTAGCT
**CHEK2_9F**	TACACGACGCTCTTCCGATCTAATGTTGAAACAGAAATAGAAATTTTGAAA
**CHEK2_9R**	AGACGTGTGCTCTTCCGATCTGGCGTTTATTCCCCACCA
**CHEK2_10F**	TACACGACGCTCTTCCGATCTGGGGAGAGCTGTTTGACAAA
**CHEK2_10R**	AGACGTGTGCTCTTCCGATCTGACAGTAAAACATTCTCTGGCTTTAAG
**CHEK2_11F**	TACACGACGCTCTTCCGATCTTTCATGAAAACGGTATTATACACCGT
**CHEK2_11R**	AGACGTGTGCTCTTCCGATCTGGGGTTCCACATAAGGTTCTC
**CHEK2_12F**	TACACGACGCTCTTCCGATCTGATTTTGGGAGAGACCTCTCTC
**CHEK2_12R**	AGACGTGTGCTCTTCCGATCTCCCACTAAGGCAGATAAAAAGAATAAC
**CHEK2_13F**	TACACGACGCTCTTCCGATCTTGGACTGCTGGAGTTTAGGA

Table S4. Tiling primers. For each of the plasmid libraries from non-selective and selective pools, primers carrying a binding site for Illumina sequencing adaptors were used to amplify short template amplicons (tiles) of ~150 bp such that the union of tile positions internal to the priming sites covered the entire ORF.
