# Supplementary material for: A missense variant effect map for the human tumor-suppressor protein CHK2
Source: Am J Hum Genet. 2024 Dec 5;111(12):2675–92. doi: 10.1016/j.ajhg.2024.10.013 (PMC11639082; doi:10.1016/j.ajhg.2024.10.013)
Supplement: Document S2. Article plus supplemental information [file mmc4.pdf]

A missense variant effect map for the human tumor-suppressor protein CHK2

Authors

Marinella Gebbia, Daniel Zimmerman, Rosanna Jiang, ..., Fergus J. Couch, Haico van Attikum, Frederick P. Roth

Correspondence

fritz@pitt.edu

***CHEK2* missense variants have been linked to breast and other cancers; however, ∼90% are classified as variants of uncertain significance. To explore sequence-structure-function relationships and to provide new evidence for clinical variant classification, we used a yeast-complementation assay to test over 8,000 *CHEK2* coding variants.**

Gebbia et al., 2024, The American Journal of Human Genetics *111*, 2675–2692
December 5, 2024

© 2024 The Authors. Published by Elsevier Inc. on behalf of American Society of Human Genetics.
https://doi.org/10.1016/j.ajhg.2024.10.013

A missense variant effect map for the human tumor-suppressor protein CHK2

Marinella Gebbia,[1,2,3,11] Daniel Zimmerman,[1,2,3,11] Rosanna Jiang,[1,2,3] Maria Nguyen,[1,2,3] Jochen Weile,[1,2,3] Roujia Li,[1,2,3] Michelle Gavac,[1,2,3] Nishka Kishore,[1,2,3] Song Sun,[1,2,3] Rick A. Boonen,[4] Rayna Hamilton,[5] Jennifer N. Dines,[6] Alexander Wahl,[7] Jason Reuter,[7] Britt Johnson,[7] Douglas M. Fowler,[6,8] Fergus J. Couch,[9] Haico van Attikum,[4] and Frederick P. Roth[1,2,3,10,*]

Summary

The tumor suppressor *CHEK2* encodes the serine/threonine protein kinase CHK2 which, upon DNA damage, is important for pausing the cell cycle, initiating DNA repair, and inducing apoptosis. CHK2 phosphorylation of the tumor suppressor BRCA1 is also important for mitotic spindle assembly and chromosomal stability. Consistent with its cell-cycle checkpoint role, both germline and somatic variants in *CHEK2* have been linked to breast and other cancers. Over 90% of clinical germline *CHEK2* missense variants are classified as variants of uncertain significance, complicating diagnosis of CHK2-dependent cancer. We therefore sought to test the functional impact of all possible missense variants in CHK2. Using a scalable multiplexed assay based on the ability of human CHK2 to complement DNA sensitivity of *Saccharomyces cerevisiae* cells lacking the *CHEK2* ortholog, *RAD53*, we generated a systematic "missense variant effect map" for *CHEK2* missense variation. The map reflects known biochemical features of CHK2 while offering new biological insights. It also provides strong evidence toward pathogenicity for some clinical missense variants and supporting evidence toward benignity for others. Overall, this comprehensive missense variant effect map contributes to understanding of both known and yet-to-be-observed CHK2 variants.

Introduction

DNA lesions activate cell-cycle checkpoints, which are important for maintaining genome integrity.[1–3] *CHEK2* (MIM: 604373), a tumor suppressor gene encoding the serine/threonine checkpoint kinase 2 (CHK2), is an important checkpoint regulator of DNA repair, cell-cycle regulation, and apoptosis in response to DNA damage.[4] Germline variants in *CHEK2* have been linked to multi-organ cancer predisposition,[5] and *CHEK2* is now typically included with *BRCA1*, *BRCA2*, and *PALB2* as a breast cancer risk gene.[6,7]

Extensive sequencing has led to the identification of many *CHEK2* variants, both rare and common. Although *CHEK2* founder mutations c.1100delC (p.Thr367Metfs*15) and c.470T>C (p.Ile157Thr) have been shown to increase breast cancer risk by 2.6- and 1.4-fold, respectively,[7–9] the extent to which the vast majority of *CHEK2* variants are associated with elevated risk remains unclear. Indeed, 94.5% of the 1,519 *CHEK2* missense variants reported on ClinVar[10] are classified as variants of uncertain significance (VUS), limiting genetic diagnosis of CHK2-dependent cancers.

According to current guidelines from the American College of Medical Genetics and Association of Molecular Pathology (ACMG/AMP), cell-based or *in vitro* functional assays of variant impact are one of the stronger forms of evidence for clinical variant interpretation. Although functional assay results are typically unavailable for rare variants, mutational scanning technologies have made it possible to systematically test variants at a large scale.[11,12] The resulting variant effect maps, measuring the functionality of nearly every possible coding variant, can provide evidence "proactively" (even in advance of the first clinical presentation of a variant). A systematic evaluation of clinical missense variants in tumor suppressors *TP53*, *BRCA1*, *PTEN*, and *MSH2* found that, of variants that were previously annotated as VUS and had variant effect map evidence, more than half could be more informatively classified.[13,14]

To assess the functional consequences of all possible mutations in *CHEK2*, we exploited a yeast-based complementation assay. *CHEK2* is the human ortholog of *Saccharomyces cerevisiae RAD53*, which also helps to maintain genome integrity after DNA damage.[15] Yeast strains lacking *RAD53* lose viability and are unable to recover from genotoxic stress.[16] Despite one billion years of divergence between yeast and humans, yeast functional complementation assays have been shown to detect ~60%[17] of pathogenic variants at a stringency where 90% of variants identified as damaging are pathogenic.[17–20] Wild-type (WT)

[1]The Donnelly Centre, University of Toronto, Toronto, ON, Canada; [2]Department of Molecular Genetics, University of Toronto, Toronto, ON, Canada; [3]Lunenfeld-Tanenbaum Research Institute, Sinai Health, Toronto, ON, Canada; [4]Leiden University Medical Center, Leiden, the Netherlands; [5]Woods Hole Oceanographic Institution, Woods Hole, MA, USA; [6]Department of Genome Sciences, University of Washington, Seattle, WA, USA; [7]Invitae Corp, San Francisco, CA 94103, USA; [8]Department of Bioengineering, University of Washington, Seattle, WA, USA; [9]Mayo Clinic, Rochester, MN 55905, USA; [10]Department of Computational and Systems Biology, University of Pittsburgh School of Medicine, Pittsburgh, PA, USA
[11]These authors contributed equally
*Correspondence: fritz@pitt.edu
https://doi.org/10.1016/j.ajhg.2024.10.013.
© 2024 The Authors. Published by Elsevier Inc. on behalf of American Society of Human Genetics.
This is an open access article under the CC BY license (http://creativecommons.org/licenses/by/4.0/).

human CHK2 restores the loss of Rad53 activity after DNA damage is induced in the presence of methyl methanesulfonate (MMS). This complementation relationship, previously exploited at smaller scale,[21] offers a facile assay to test the functional impact of CHK2 missense variants.

We integrated this functional assay within a deep mutational scanning framework[11,22] to produce a comprehensive functional variant effect map of human CHK2 in the presence of MMS. We validated this map based on agreement with known biochemical features of CHK2, smaller-scale functional assay results, and the ability to separate pathogenic and benign variants.

Material and methods

Yeast strains and plasmids

An *S. cerevisiae* strain (*MAT***a** *sml1*Δ::kanMX *rad53*Δ::hygMX) was used as a host for the *CHEK2* variant library. The double-deletion strain was generated by PCR replacement of the *RAD53* gene with the hygromycin selectable marker (cassette) in the single-deletion strain (*MAT***a** *sml1*Δ::kan*MX*) derived from the yeast knockout collection.[23]

The *CHEK2* open reading frame (ORF) clone, corresponding to UniprotKB accession O96017 (RefSeq: NM_007194), was obtained from the Human ORFeome v.8.1 library.[24] A Gateway compatible yeast expression vector, pHYC-Dest2 (CEN/ARS-based, *ADH1* promoter, and *LEU2* marker), was used for the complementation assay.

WT reference or mutated disease-associated versions of the *CHEK2* ORFs were transferred into pHYC-Dest2 by Gateway LR reactions. After confirmation of ORF identity and expected mutations by Sanger sequencing, the expression clones were transformed into the double-deletion yeast strain in parallel with an "empty" expression vector control (bearing the counterselectable ccdB marker controlled by a bacterial promoter).

CHEK2 yeast complementation assay

Single colonies of yeast transformed with vectors expressing *CHEK2* cDNAs were picked from SC-Leu+Kan+Hyg with 2% glucose plates and grown at 30°C to saturation (overnight) in liquid medium SC-Leu+Kan+Hyg with 2% glucose. Each culture was then adjusted to an OD_{600} of 0.2 and diluted 1:5 with five serial dilutions. 4 μL of these cultures was spotted on agar plates of SC-Leu containing 2% glucose and a final concentration of 0.007% MMS (spotting assay). The plates were incubated at 30°C and after imaging, the comparison of the effect of MMS on growth/fitness of yeast—carrying either *CHEK2* variants, *CHEK2*-WT, or an empty vector—was made starting from day 3 (Figure 1).

Construction of codon randomized CHEK2 variant libraries

As a first step of the TileSeq framework, a pooled random-codon mutagenesis method (precision oligo-pool-based code alteration, or POPCode)[11] was used to construct the *CHEK2* variant libraries. Mutagenesis was separately applied in four regions of the *CHEK2* ORF (which has a total length of 1,632 bp) that are each ~450 bp in length (encoding ~150 amino acids) (Figure S1), and we followed each step of the framework below separately to generate four distinct regionally mutagenized libraries. Oligonucleotides (28–38 bp long), each containing central NNK-degenerate codons and collectively covering the entire *CHEK2* coding sequence, were designed to achieve a common melting temperature for each NNK-flanking subsequence using the POPCode oligo suite tool.[11] Oligonucleotides for each of the four *CHEK2* regions were pooled and phosphorylated to perform regional POPCode mutagenesis. Phosphorylated oligonucleotides were annealed to a uracilated full-length template of WT *CHEK2* using KAPA HiFi Uracil+ DNA polymerase (KapaBiosystems) and a mix of dNTP/dUTP from each regional oligo pool. After annealing, KAPA HiFi Uracil+ DNA polymerase (KapaBiosystems) was used to fill in the gaps and Taq DNA ligase (NEB) was applied to seal the nicks. The samples were treated with uracil-DNA-glycosylase from NEB to degrade the original uracilated template. The newly POPCode-mutagenized strand was then amplified with primers containing attB sites. The mutagenized attB-PCR products were next cloned *en masse* into the entry vector pDONR223 by Gateway BP reactions, generating regional entry libraries that had ~150,000 clones per region, with the goal of achieving high complexity, i.e., so that each variant would be present in an average of 50 or more independent clones. These Gateway-entry clone libraries were then transferred to a pHYC-Dest2 expression vector by *en masse* Gateway LR reactions, generating regional expression libraries from >300,000 transformants per region in order to maintain pool complexity. Both Gateway-entry and -expression library transformations used NEB5α *Escherichia coli* cells (NEB) with selection on LB agar plates using spectinomycin and ampicillin, respectively. Both Gateway-entry and -expression libraries and the yeast transformants were grown on 245 × 245-mm^2 bioassay dishes (Corning). Finally, regional expression libraries were transformed into the *S. cerevisiae* double-mutant strain *sml1*Δ *rad53*Δ using the EZ Kit Yeast Transformation kit (Zymo Research) to obtain ~1,000,000 clones per region, thus maintaining library complexity. Yeast transformants for each regional library were pooled separately and grown in synthetic complete (SC) medium without leucine (US Biological) as the non-selective medium. In an initial attempt at mutagenesis of the fourth region, frequent frameshift mutations were observed (data not shown). After attributing this to a TG-rich segment in the last 23 nucleotides of the *CHEK2* ORF, we designed a synthetic clone that was codon optimized to limit potential secondary structures before repeating regional POPCode mutagenesis for the fourth region. No enrichment for frameshifts was subsequently observed in the final version of the fourth regional mutagenesis library.

Multiplexed assay for CHK2 variant function

The yeast-based functional complementation assay we used to evaluate *CHEK2* missense variants was similar to that of Roeb et al.,[21] who assayed recovery of cells from DNA-damage treatment (0.014% MMS in liquid medium). For this study, we selected cells using SC-Leu solid-agar medium plates with 0.007% MMS. More specifically, from the yeast transformants grown in non-selective conditions, two replicates of ~9M cells from each of the regional transformant pools were plated onto selective SC-Leu-medium plates with an MMS concentration of 0.007% and incubated at 30°C for 3 days. After pooling the colonies of each replicate, plasmid DNA was extracted from ~4M cells per region from both non-selected and selected pools and used for subsequent PCR amplification of specific targeted tiles. Controls were assayed, in parallel, using the *S. cerevisiae sml1Δ rad53Δ* strain transformed

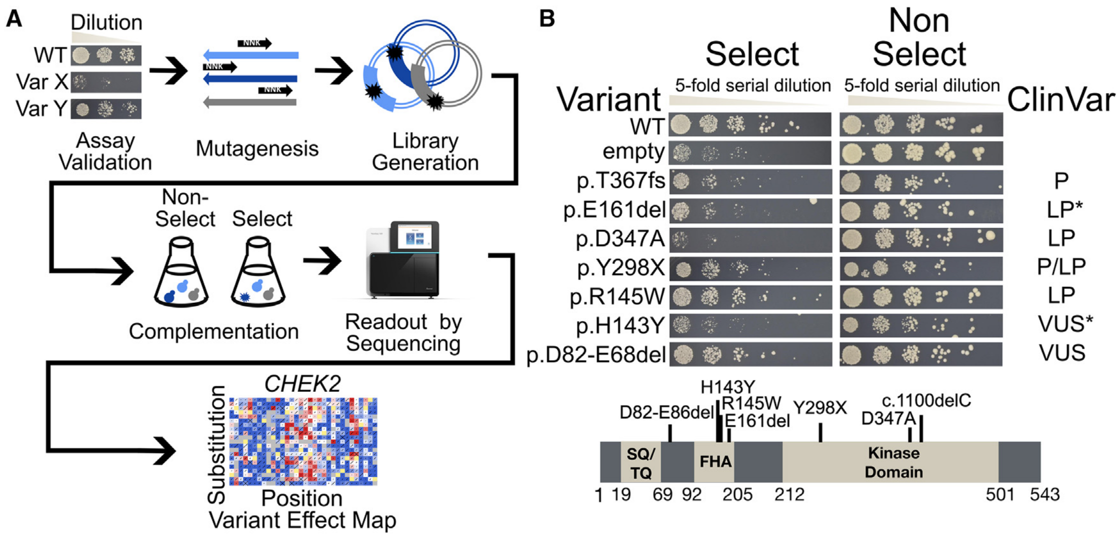

Figure 1. Assay validation and generation of the CHK2 variant effect map
(A) Overview of the TileSeq framework that was followed to produce the CHK2 variant effect map.
(B) Yeast-based functional complementation assay for CHK2 domains labeled with tested variants. The *sml1Δ rad53Δ* yeast strain was transformed with the expression vector pADH1-Leu carrying wild-type (WT) *CHEK2* or the empty pADH1-Leu expression vector (empty). The selective condition was 0.007% MMS, and the non-selective condition was 2% DMSO. Yeast growth was assessed by spotting serial dilutions of yeast cells on selective media and incubation for 3 days. P, LP, and VUS indicate pathogenic, likely pathogenic, and variant of uncertain significance, respectively. We note LP* for p.Glu161del (p.E161del) because it has been annotated variously as LP, P, and VUS, and note VUS* for p.His143Tyr (p.H143Y) because one of six annotations in the ClinVar database was LP, while the others were VUS. The CHK2 protein domain graphic is based on an image from Wang et al.[25] and information from Boonen et al.[26]

with an empty pHYC-Dest2 expression vector as the negative control and WT *CHEK2* in the pHYC-Dest2 vector as the positive control. The control strains were grown in Petri dishes of solid non-selective and selective media during the same incubation period as the large-scale variant library regional assays.

Quantifying variant abundance

Each region was conceptually divided into four tiles, each approximately 151 nt long. Each tile was amplified from the plasmid DNA extracted from each pool of yeast from non-selective and selective conditions, amplifying targeted tiles with primers carrying an Illumina sequencing adapter binding site (Table S4). Next, a unique Illumina sequencing adapter was added to each tile via a subsequent "indexing PCR." Equal amounts of the tiled indexed PCR products were pooled together, and the resulting pooled library of an expected size of ~300 bp was purified using a 4% EX e-gel (Life Technologies) followed by MinElute Gel Extraction (Qiagen). After the library quantification via NEBNext Library Quant Kit (NEB), paired-end sequencing was performed on the tiles of each region with a sequencing depth of ~2M reads per tile using an Illumina NextSeq 500 instrument via a NextSeq 500/550 High Output Kit v2.

Deriving functional scores for variants

An analysis pipeline called TileseqMave (v.1.0.0) was used for the generation of the variant effect map (code, installation instructions, and documentation are available on Github at https://github.com/rothlab/tileseqMave). TileseqMave uses bowtie2[27] forward and reverse reads to the *CHEK2* reference sequence. For each divergent base call, the posterior probability of being a true variant is calculated. Variants with posteriors exceeding a threshold of 0.9 are counted. For each codon change, a "marginal count" is calculated, i.e., the number of times the change was observed irrespective of other co-occurring variants. To calculate the frequency of each codon change, the marginal count for each mutation is normalized by its "effective sequencing depth," i.e., the number of reads in which the variant call at the given position was decidable. An error-corrected enrichment log ratio is then calculated by subtracting the frequency of the variant in the corresponding WT control from the post- and pre-selection frequencies and calculating the log ratio between the latter. (Where multiple sequencing runs were required to obtain sufficient read counts, we subtracted counts from matched WT control libraries before aggregating counts.) Finally, the enrichment log ratio is rescaled such that, after rescaling, synonymous variants have a median score of 1 and nonsense variants a median score of 0. For each variant, measurement error is regularized using the method described by Baldi and Long[28] and propagated via bootstrapping.

To include only well-measured results, we filtered out variants that were seen in fewer than ten reads in the pre-selection library and those for which pre-selection frequencies were statistically indistinguishable from those in the WT control. We also removed variants for which replicates diverged by more than three times the amount expected based on their Poisson variance, given their underlying read count.

To identify the highest-quality scores for clinical analysis, we removed variants for which the interval of a variant's score plus and minus error included 0.5 (the midpoint between synonymous and nonsense).

Conservation, solvent accessibility, and protein stability

To quantify evolutionary conservation across CHK2 positions, we used ConSurf.[29] As ConSurf considers structural homology when choosing homologs for comparison, we provided a predicted structure (using AlphaFold v.2022-11-01, Monomer v.2.0 pipeline;

UniProt: O96017). ConSurf assigned a conservation score to each position along CHK2 ranging from highly conserved scores below −0.47 to lowly conserved scores above 0.91. The median functional score for every position with a conserved grade was compared to functional scores in non-conserved positions using a Wilcoxon rank-sum test.

Solvent accessibility was calculated using FreeSASA (v.2.02, October 22, 2017) using the aforementioned CHK2 structure.[30] Positions with relative side-chain accessibility to solvent below 20% were considered inaccessible and positions greater than 50% considered accessible. Median functional scores for positions in the forkhead-associated (FHA) domain (residues 92–205) and the kinase domain (residues 212–501) were stratified into solvent-accessible and solvent-inaccessible positions before being plotted and compared by Wilcoxon rank-sum test.

ΔΔG thermostability predictions were made for all possible CHK2 missense variants using FoldX software (v.4.0).[31] ΔΔG scores above 0 predict protein destabilization, while scores below 0 are predicted to retain structure. With the median functional score and median stability score at each position as the input, we performed a moving window analysis across CHK2 that took the median value from a bin width of ten positions and moved at a step size of one residue. Correlation between functional score windows and ΔΔG stability scores was evaluated by Spearman correlation.

Reference set of clinically annotated variants

A set of clinically annotated *CHEK2* variants was provided by Invitae using their Sherloc v.6.0 variant classification system, which captures evidence of strength in terms of points toward pathogenicity or benignity.[32] Variants with at least four pathogenic points were included in the positive reference set (including variants annotated as either pathogenic or likely pathogenic variants), and variants with at least three benign points were included in the negative reference set (including variants annotated as either benign or likely benign). From our sets of 21 positive and 39 negative reference variants, 12 positive and 28 negative variants had high-confidence functional scores.

Calibrating log-likelihood ratios to ACMG evidence strength

To derive estimates of the strength of evidence toward pathogenicity annotation that are both compatible with a Bayesian clinical annotation approach and are more continuously quantitative than points-based approaches, we estimated the log-likelihood ratio of pathogenicity (LLR) for each variant. More specifically, probability density functions were separately estimated for scores from the positive and negative reference variant sets using kernel density estimation (using a Gaussian kernel with a bandwidth determined by biased cross-validation). For each variant, the LLR could then be calculated as the log ratio of the two probability densities regularized against a uniform distribution. LLR values were then compared to threshold values defined by the ACMG/AMP variant classification framework using the strategy of Tavtigian et al.[33] as subsequently adapted by van Loggerenberg et al.[34]

Structural modeling and analysis

We used OpenPyMol to visualize structural models of CHK2 using previously reported structural models (PDB: 3i6u,[35] 2CN5,[36] and 2CN8[36]). Structural models were colored according to the median variant effect map score for each residue.

Results

To assess the impact of human *CHEK2* variants in response to DNA damage, we carried out functional assays for nearly all possible missense variants (Figure 1A). Here, after confirming validity of a previously published assay, we describe the large-scale variant testing process in greater detail before relating the resulting functional scores to known biochemical and structural features of CHK2 and to clinical variant pathogenicity.

Confirming and validating a yeast-complementation assay for human CHEK2

Yeast is dependent on *RAD53* for both viability and response to stress[16] and for resistance to DNA damage induced by MMS.[37,38] Building on observations that expression of human CHK2 in yeast rescues the loss of *RAD53*[16] and on the observation that deletion of yeast *SML1* enables the viability of *rad53*Δ yeast strains while retaining their sensitivity to DNA damage,[39,40] Roeb et al. measured the ability of dozens of CHK2 variants to rescue the MMS sensitivity phenotype of an *sml1*Δ *rad53*Δ yeast strain.[21] This assay was later used by Delimitsou et al. to test over 100 CHK2 missense variants.[9] Here we recapitulated the complementation assay of Roeb et al. with minor modifications (see material and methods) and showed for a set of control variants that (with the single exception of pathogenic variant p.Arg145Trp) the expected impact on MMS sensitivity was observed (Figure 1B). It should be noted that spotting assays are semi-quantitative, and interpreting variants with intermediate changes in growth (i.e., p.Thr367Metfs*15, p.Tyr298*, p.Glu161del) can be subjective. Regardless, the spotting assay results gave us sufficient confidence in the assay reliability to proceed to large-scale implementation.

A proactive missense variant effect map for CHK2

To exploit the yeast-based complementation assay at scale, we adopted the TileSeq framework[11] (see material and methods), using the following steps.

First, we generated variant libraries of *CHEK2* missense variants by POPCode mutagenesis,[11] with each library focused on one of four *CHEK2* regions (Figure S1), each encoding ∼150 amino acids. Within each mutagenized region we sequenced short (∼151 nt) "tile" segments which collectively span the region.

Second, we used recombinational cloning to transfer each of these mutagenized *CHEK2* amplicon libraries en masse into the appropriate yeast expression vector, then individually transformed each vector pool into *sml1*Δ *rad53*Δ yeast and stored frozen aliquots of each transformed yeast pool ("pre-selection pools").

Third, to obtain post-selection pools, we grew yeast pools on solid media with 2% glucose and 0.007% MMS to select for cells expressing functioning CHK2 variants.

Fourth, variant frequencies were obtained by sequencing ~2M or more reads for each *CHEK2* tile from each pre- and post-selection yeast pool and also from a control WT sequence library.

Clones in the pre-selection pool were estimated to contain an average of 0.3 amino acid changing variants per clone, with less than 5% of clones bearing multiple variants (Table S1). Estimates of variant frequency in the pre-selection library were strongly correlated between sequencing libraries from replicate cell pools (Pearson correlation coefficient [PCC] = 0.98; $p < 1e-320$ (Figure S2A). To score the impact of selection on each variant, we measured the log ratio of variant frequency (log(ϕ)) in each post-selection pool relative to the corresponding pre-selection pool. To remove less-well-measured scores, e.g., where pre-selection abundance was too low, we applied filtering criteria. Replicate log(ϕ) scores were significantly correlated (PCC = 0.64; $p < 1e-320$) (Figure S2B). Log(ϕ) scores in each region were next calibrated to yield more interpretable functional scores such that a functional score of 1 corresponds to the mode of synonymous variants, and a functional score of 0 corresponds to the mode of nonsense variants (excepting positions near the C terminus as discussed below). Nonsense variants up to position 489 exhibited highly damaging scores (mean score of 0.0 ± 0.5 SD). However, nonsense variants from position 490 to the C terminus appeared less damaging (mean score 0.7 ± 0.5 SD), suggesting that these truncating protein variants are able to function (although they might still be damaging at the endogenous locus in human cells, as noted in the discussion) (Figure S3). After excluding nonsense variants beyond position 489, nonsense and synonymous variants showed strongly shifted functional score distributions, albeit with some overlap (Figure 2A) which we revisit later before exploring clinical applications (Figure S4).

Together, this process yielded a missense variant effect map with functional scores for 7,955 amino acid substitutions and 419 nonsense mutations (Figures 2 and S5), corresponding to over 77% of all possible amino acid changes (Figure S6). Most missense variants appeared "synonymous-like" (~64.5% of variants scored above 0.5), while a substantial proportion of variants yielded "nonsense-like" functional scores, with ~25% of variants having scores below 0.2. The variant map covered 2,837 (89%) of the 3,182 amino acid substitutions that are achievable by a single-nucleotide variant and therefore more likely to be observed in humans.

Functional scores reflect known structural and biochemical features of CHK2

Scores in the variant effect map generally agreed with conservation and known structural/biochemical features of CHK2. For example, lower-scoring variants indicated in dark blue (Figure 2B) occurred more commonly at conserved positions (|Δ median| = 0.35, $p = 1.97e-10$, Wilcoxon rank-sum test) (Figure 3A). As expected, solvent-accessible residues scored higher than non-accessible positions in the FHA (amino acids 92–205) and kinase (amino acids 212–501) domains, as well as across the entire protein (|Δ median| values of 0.39, 0.28, and 0.23, respectively; p = 0.004, 1.3e−5, and 3.4e−7; Wilcoxon rank-sum test) (Figure 3B). As one example, the founder variant p.Ile157Thr (allele frequency ~1%), which is buried at the center of the hydrophobic core, showed a low functional score for most polar substitutions but not for hydrophobic substitutions. Visualizing the dimeric CHK2 crystal structure (PDB: 3i6u) with each residue colored according to the median map score for each position further supports the expected finding that the map tended to give more damaging scores to buried residues (Figures 3C and 3D). Positions within the kinase domain exhibited significantly lower functional scores than the rest of the protein (|Δ median| = 0.11 with $p = 1.6e-4$, Wilcoxon rank-sum test). Although positions within the shorter FHA domain showed a numerically similar reduction in functional score, this result was not significant (|Δ median| = 0.11 with $p = 0.21$, Wilcoxon rank-sum test) (Figure S7).

We next compared CHK2 functional scores with computational predictions of variant impact on protein stability. Functional scores showed the expected negative correlation with predicted change in folding energy (ΔΔG from FoldX[41]; Spearman's $R = -0.43$; $p = 1.0e-308$). It has been previously noted that positions where substitutions impact overall functionality, but not stability, may correspond to positions that are important for catalysis, regulatory modification, structural flexibility, or physical interaction with other biomolecules.[34,42,43] Evaluating agreement between functional and ΔΔG scores within successive sequence "windows" identified several regions where substitutions impacted function more profoundly than stability (Figure 4), including positions 246–249, 345–347, 368–370, and 392–394, which corresponded to known kinase motifs VAIK, HRD, DFG, and APE, respectively (see further analysis below). Additionally, it is possible that positions 138–144 and 191–202 in the FHA domain are involved in CHK2 homodimerization, given the highly damaging functional scores despite moderate ΔΔG predicted impacts on stability. Positions 93–97, 115–117, and 164–169 in the FHA domain might also be involved in CHK2 homodimerization, with variants leading to both unstable ΔΔG predictions and damaging functional scores (Figures S8A and S8B). Because the FHA-FHA dimerization interface is reportedly centered on the aromatic side chains of Trp97 and Phe202 and because dimerization is required for CHK2 activation, it is likely that all of these positions are important for dimerization. Additionally, Ser140, which is required for dimer dissociation after activation, has minimal predicted impact on protein stability despite low functional scores.[35,36] Immediately N-terminal to the FHA domain, prior to any known ordered secondary structures, positions 64–70 were found to be damaging *in vitro* but stable by ΔΔG. It is possible that positions 64–70 form an ordered secondary structure that has not yet

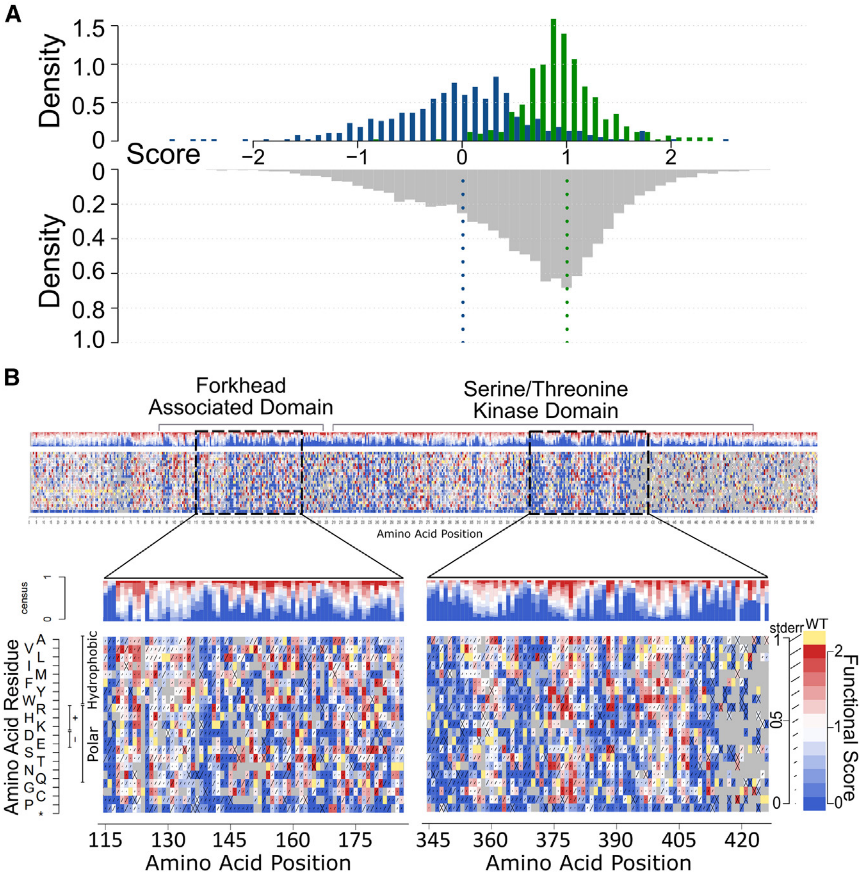

Figure 2. Experimental variant effect maps of CHK2

(A) Functional scores for synonymous (green), nonsense (blue), and missense (gray) variants from positions 2 to 489 in the original map were plotted as a histogram with scores on the x axis and density on the y axis. The dashed vertical blue and green lines overlaid on the missense distribution mark the median nonsense and median synonymous score, respectively.

(B) A preview of the full-length original CHK2 variant effect map with magnified views of segments of the FHA domain (positions 116–186) and kinase domain (positions 346–426). An enlarged version of the map can be found in Figure S5. Each heatmap shows functional scores for every possible amino acid substitution and nonsense mutation (bottom row). Above each heatmap is a consensus summary of the distribution of functional scores at each position. As shown in the legend (right), a functional score of 0 (blue) corresponds to the median score of nonsense variants; a score of 1 (white) corresponds to the median of synonymous variants; scores above 1 (red) are considered hypercomplementing; gray indicates missing data; and the length of the diagonal line on each block indicates estimated each score's estimated standard error.

been identified (due to the lack of full-length CHK2 crystal structures). Interestingly, positions 15–25 showed a slight decrease in functional score accompanied by highly unstable ΔΔG predictions, despite being in a predicted disordered region from positions 1 to 66. A few unstable regions with damaging functional scores aligned with known secondary structures, specifically highlighting a beta strand at positions 307–309 and an alpha helix at 407–422.

CHK2 activation requires phosphorylation of Thr68 in the SQ/TQ cluster domain (SCD) by the DNA-damage-activated kinase ATM. It has been shown that ATM-induced phosphorylation causes transient dimerization of CHK2, thereby inducing kinase activation through autophosphorylation of Thr383 and Thr387.[35,36] Consistent with this model, the phosphomimetic variant p.Thr68Glu showed near-WT function with a map score of 0.8, while the phospho-dead variant p.Thr68Ala showed complete loss of function with a score of −0.6 (Figure S9). Positions critical for dimerization, Thr68 and Ile157,[8,44] as well as the catalytic residue Asp347 and phospho-acceptors Thr383 and Thr387, all have low median scores of 0.2, 0.4, −0.6, 0.0, and −0.6, respectively. The activation loop, corresponding to positions 371–391 in the kinase domain, displayed variable tolerance to substitutions: 11 residues appeared highly tolerant to variation (with median scores greater than 0.8) while seven positions, including Thr383 and Thr387, had median functional scores less than 0.2. Because autophosphorylation accompanying CHK2 activation requires ATP binding via hydrogen bonding and hydrophobic interactions,[1,45] we examined residues that had been previously identified as important for CHK2 activation on the basis of CHK2 crystal structures, based on proximity to either complexed ADP or the competitive ADP inhibitor debromohymenialdisine (DBQ)[36] (Table S2). As expected, a higher proportion of variants at ADP/DBQ contact sites had damaging functional scores (65%; 171 variants out of 263) compared to the rest of the kinase domain (38%; 1,513 variants out of 3,920). Similarly, a higher proportion of ADP/DBQ positions had a median map score below the damaging threshold of 0.5 (81%; 13 out of 16) than was observed for all other positions in the kinase domain (30%; 82 out of 274).

CHK2 map scores correlate with mutational hotspots in the kinase domain

In a previous study, the combination of multiple sequence alignments of candidate tumor suppressors and missense mutation rates at each position were used to identify mutational hotspots.[46] Some of the 23 mutational hotspots identified overlap with known protein features. For example, positions 247 and 248 in the VAIK motif, as well as position 368 in DFG, are located in ADP binding sites, and the APE-6 hotspot is located in the kinase

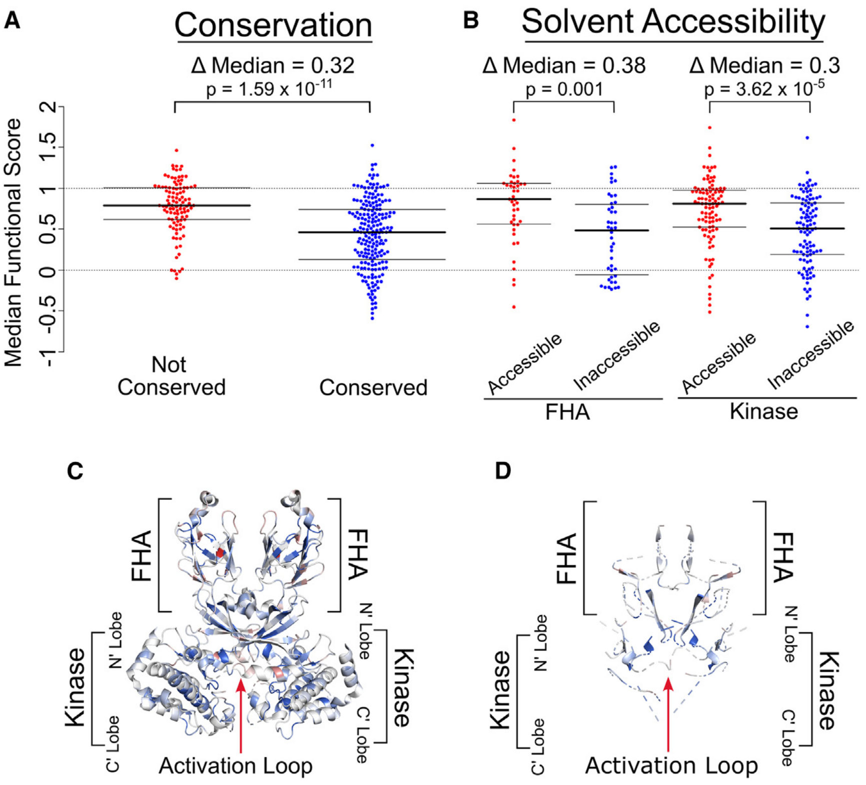

Figure 3. CHK2 map scores agree with expected effects of conservation and solvent accessibility

(A) Median CHK2 functional scores were calculated and stratified based on each position's conservation score. Conservation scores were derived from ConSurf multiple sequence alignments based on an AlphaFold predicted PDB file for CHK2. Residues labeled "Not Conserved" have ConSurf scores above 0.91, while "Conserved" positions are below −0.47. The *p* value was calculated via a Wilcoxon rank-sum test comparing functional scores at "Not Conserved" positions to "Conserved" positions

(B) Solvent accessibility was determined using FreeSASA software (v.2.02, October 22, 2017) on the AlphaFold (v.2022-11-01, Monomer v2.0 pipeline; UniProt: O96017) CHK2 structure. Residues with less than 20% accessible surface area (ASA) for their side chain were considered "inaccessible" while those with above 50% side-chain ASA are "accessible." Results were separated into the FHA domain (positions 92–205) and kinase domain (positions 212–501). *p* values comparing functional scores at solvent accessible and inaccessible positions were calculated via a Wilcoxon rank-sum test for positions in the FHA domain or the kinase domain.

(C) Crystal structure of the CHK2 dimer with colored residues based on the median functional score of each position.

(D) Crystal structure of the CHK2 dimer with blue residues for positions with a dimer interface burial greater than 0%.

activation T loop at position 386. Of the 23 CHK2 hotspots identified, all 16 that fell within a named motif (APE, VAIK, HRD, DFG, and the three G positions within the GxGxxG motif) scored as intolerant to variation in our map (median scores below 0.5). Median values of the hotspot positions within the VAIK, HRD, and APE motifs (located at positions 246–249, 345–347, 392–394, respectively) exhibited damaging scores ranging from −0.76 to 0.35 (Table S3). Within the GxGxxG motif, hotspot positions Gly227, Gly229, and Gly232 had damaging median scores of 0.19, 0.06, and −0.35, respectively, while hotspot positions corresponding to the DFG motif medians were −0.62 (Asp368), −0.42 (Phe369), and 0.07 (Gly370). These 16 mutational hotspots are evidently important for CHK2 function, as demonstrated by median scores that were significantly lower than those of CHK2 overall ($|\Delta$ median$| = 0.95$, $p = 2.0e{-41}$, Wilcoxon rank-sum test) (Figure 5). Of the six additional hotspot positions identified by Hudson et al.[46] but not listed above, only three appeared damaging in our map: HRD+5 (Asn352), HRD+7 (Leu354), and APE-6 (Gly386) showed median scores of 0.04, −0.22, and 0.05 respectively.

Relating the CHK2 map to previously reported functional assays

The functional effects of CHK2 missense variants have been previously assessed in a variety of assays that include rescue of growth (of yeast cells) under DNA damage[9,21] and mammalian cell-based protein stability and kinase activity assays.[26,47] Our map scores showed modest but significant correlation with the Delimitsou et al. yeast-growth-based study ($R = 0.44$, $p = 2.1e{-6}$),[9] but no significant correlation with the Roeb et al. study ($R = 0.19$, $p = 0.38$).[21] Variants classified as "functional" by Delimitsou et al. had a median score of 0.95 in our map, and variants deemed "non-functional" had a median score of 0.34 (Figure 6A). Of 108 variants, 27 (∼25%) had conflicting classifications between our assay and that of Delimitsou et al., and, after filtering out variants with intermediate functional scores or low-confidence measurements, 14 (∼19%) of the remaining 75 variants had conflicting results. Of these 14 variants, eight were considered damaging in our assay, seven of which have been reported to ClinVar as VUS or have conflicting interpretations. One of the variants, p.Thr68Ala, occurs at a CHK2 phosphorylation site involved in CHK2 homodimerization and might (as a phospho-dead variant) be expected to impact CHK2 function, yet it is annotated as VUS.

We also showed modest but significant correlation with assays by Boonen et al. of the ability of CHK2 to phosphorylate the downstream substrate KAP1 in mammalian cells[26] ($R = 0.41$, $p = 0.007$) (Figure 6B). Variants classified as "functional" by Boonen et al. had a median score of 0.97 in our map, in contrast to a median map score of 0.32 for the variants they called "damaging".[26] Eleven out of 41 (∼27%) variants had discordant results between our assay

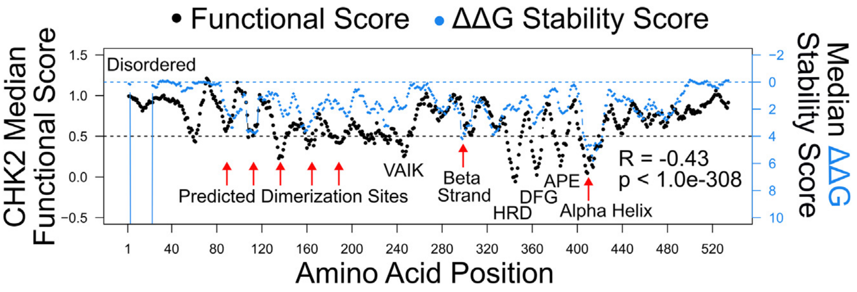

Figure 4. Regions with functional but not (predicted) protein stability impacts highlight positions in the FHA domain important for dimerization
Stability scores (estimated ΔΔG folding energy values) were generated via FoldX software using the AlphaFold predicted protein structure of CHK2. At each position, ΔΔG was calculated for every substitution and the mean value was plotted against the average positional CHK2 functional scores in a ten-amino-acid-wide moving window analysis (with single-residue step size). Variants at positions with a ΔΔG near 0 are predicted to be typically stable, while ΔΔG values greater than 0 are predicted to be less stable. Variants with functional scores of 1 should be considered "wild-type-like," while scores near 0 should be considered to have profound loss of function. The dashed horizontal black line indicates the damaging threshold functional score of 0.5 and the dashed horizontal blue line a stable ΔΔG score of 0. Spearman's R and *p* value were calculated comparing the ten-amino-acid-wide moving windows of functional scores and ΔΔG stability scores.

and that of Boonen et al., but after removing intermediate or low-confidence variants only 6 (~23%) of the remaining 26 variants had conflicting results. We observed a low (and not significantly different from zero) correlation coefficient between our map scores and another mammalian cell-based study[47] ($R = 0.30$, $p = 0.16$) of pKAP phosphorylation and the Boonen et al.[26] protein stability assay ($R = -0.09$, $p = 0.66$) (Figure S10). It is possible that the modest correlation with results from mammalian cells reflects species-specific differences in the CHK2 pathway; however, alternative yeast-based assays also showed intermediate levels of correlation.

In a more detailed comparison of our map with the two published assay sets showing significant correlation with our map—the Boonen et al.[26] pKAP1 assay and the Delimitsou et al.[9] yeast-growth assay—we examined the 18 clinical missense variants tested in all three studies and found that nine had results that agreed in all three. All assays correctly detected many variants as damaging, e.g., p.Asp347Asn, a position known to impact kinase function,[48] and residues associated with elevated risk of breast cancer such as p.Tyr390Ser.[10,25] Interestingly, both p.Asp347Asn and p.Tyr390Ser had conflicting clinical annotations, with a mixture of either "likely pathogenic" or "pathogenic" and "VUS"' annotations. In positions adjacent to Asp347 and Tyr390, all three assays found detrimental effects for p.Arg346His and p.Ala392Val (with predominantly VUS annotations). Predicted dimerization variants p.Arg117Gly and p.Gly167Arg, which exhibited damaging scores in all three studies, are predominantly annotated as likely pathogenic or pathogenic. Only three variants—p.Arg180Cys, p.Arg181His, and p.Asn446Asp—were found to have normal function in all assays. ClinVar describes a mixture of likely benign or benign and VUS clinical annotations for all three variants, without any likely pathogenic or pathogenic classifications. Thus, our map, taken together with previous assay data, concurs with pathogenic clinical annotations for p.Arg117Gly and p.Gly167Arg, adds to the weight of evidence toward pathogenicity for p.Arg346His, p.Asp347Asn, p.Tyr390Ser, and p.Ala392Val, and provides evidence toward benignity for p.Arg180Cys, p.Arg181His, and p.Asn446Asp.

Of the remaining nine variants for which results differed between our map and either the Boonen et al. pKAP1 assay[26] or the Delimitsou et al. yeast-growth assay,[9] three substitutions (p.Asp203Gly, p.Glu239Lys, and p.Asp438Tyr) had intermediate scores in Boonen et al. but were seen as well tolerated by Delimitsou et al. and our map. Conversely, some variants with intermediate scores in our assay were observed to be damaging (p.Ile160Thr, p.Asp162Gly) by both Boonen et al. and Delimitsou et al. studies or were found to be nondamaging (p.Ile157Thr) by both. Finally, our assay found p.Arg145Trp to be well tolerated and variants p.Cys243Arg and p.Asn186His to be damaging, while Boonen et al. and Delimitsou et al. both found results opposite to ours for each of these. All nine of these variants except p.Arg145Trp (which was classified as likely pathogenic in ClinVar) either had conflicting interpretations or were classified as VUS.

Preparing variant effect map scores for clinical analysis

Although the evaluation of our map in the context of known biochemical features of CHK2 gave us confidence that it is capturing real impacts, in this process we also identified features of the CHK2 variant effect map suggesting that we should undertake further filtering before considering its scores for clinical applications. First, although most nonsense variants before position 489 had damaging scores (82%; 317 variants out of 385) and most synonymous variants appeared tolerated (95%; 402 variants out of 424), the remaining apparently misclassified nonsense and synonymous variants were problematic, suggesting errors that could also impact missense variant scores. We therefore imposed a further filtering step (see material and methods), which improved separation between synonymous and nonsense distributions (Figure S4). This decreased the number of apparently misclassified nonsense variants from 68 to 28 and the number of apparently misclassified synonymous variants from 22 to 4.

Second, we noticed that a small but appreciable fraction of missense variants received scores that were substantially

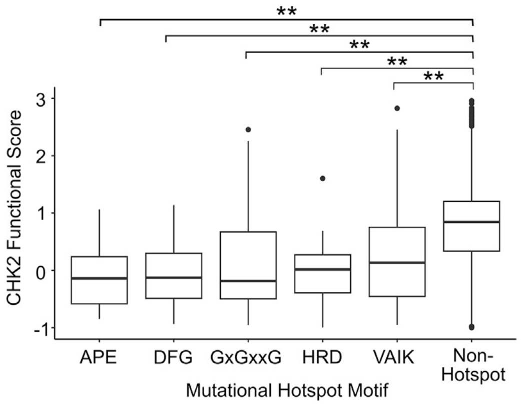

Figure 5. Known mutational hotspots in CHK2 are largely intolerant to missense variants
Mutational hotspots identified in Hudson et al.[46] were separated into distinct kinase activation motifs (APE, DFG, GxGxxG, HRD, and VAIK) and plotted against CHK2 functional scores for each variant at these positions. Boxplots depict the median functional score for each motif as well as the 25th and 75th percentiles with vertical lines extending to 1.5 times the interquartile range above and below 25th and 75th percentiles. All motifs were conserved, matching the consensus sequence observed in other kinases. Double asterisks indicate significance by Mann-Whitney U test of less than 1e−5 as compared to non-hotspot positions. The absolute delta medians for motifs APE, DFG, GxGxxG, HRD, and VAIK, as compared to non-hotspot positions, are 1.07, 1.08, 1.04, 0.96, and 0.81, respectively.

above the WT-like score of 1. More specifically, 12% of missense variants had scores that were above 1 by more than twice the median estimated standard error of all variants (i.e., had scores above 1.46). While CHK2 primarily functions as a tumor suppressor, where loss of function contributes to cancer, gain-of-function variants have been associated with drug-resistant cancers.[49,50] Variants that increase stability, activity, or expression are expected to be tolerated in our yeast-complementation assay, since they will presumably still be MMS resistant .

To assess whether variants that appear hyper-complementing in our yeast assay are likely to be deleterious in humans, we performed a previously described phylogenetic analysis method, which essentially asks whether hyper-complementing variants are more or less likely than chance to be found in orthologs of human-related species[51] Briefly, we quantitatively evaluated three models according to the Akaike information criterion (AIC; which is defined by the sum of the log-likelihood of the data and the log count of model parameters) and found the model in which hypercomplementing variants are deleterious in humans and related species to yield the lowest (best) AIC with neutral or advantageous models yielding AIC scores that were higher (worse) by 104 and 603, respectively. Thus, phylogenetic analysis suggested that hyper-complementing variants in our assay tend to be deleterious rather than neutral or even advantageous as a "better than wild-type" score might otherwise suggest. Therefore, to err on the side of caution, we also removed all hyper-complementing variants (using the aforementioned score threshold of 1.46) from all clinical analysis below.

The resulting more conservatively quality-filtered missense variant effect (“HiQ”) map scored 4,604 amino acid substitutions, corresponding to over 45% of all possible amino acid changes. It covers 1,492 (47%) of the 3,182 amino acid substitutions that are achievable by a single-nucleotide variant.

CHK2 variant effect map scores do not correlate with allele frequency in gnomAD

Mutational scanning studies can be evaluated according to whether scores are distinct for annotated pathogenic and benign variants (e.g., drawn from the ClinVar database). Unfortunately, only a limited number of benign (1) and likely benign variants (5) have been reported on ClinVar. There is precedent for supplementing reference sets with “proxy-benign” variants chosen on the basis of having a high minor allele frequency reported on gnomAD.[52] Support for this idea can be found for BRCA1, with predicted tolerated variants (least damaging 5% according to VARITY_R) showing a significantly higher allele frequency than damaging variants (most damaging 5% according to VARITY_R; $|\Delta \text{ median}| = 0.17$, $p = 1.93\text{e}{-2}$, Wilcoxon rank-sum test). However, previous analysis of *CHEK2* variants on gnomAD showed that *CHEK2* is not strongly depleted for damaging variants, i.e., that it has a low probability of being loss-of-function intolerant,[52] challenging the assumption that (even high-allele frequency) gnomAD variants are neutral. Indeed, *CHEK2* log allele frequencies were not significantly different ($|\Delta \text{ median}| = 0.24$, $p = 0.32$, Wilcoxon rank-sum test) between the least- and most-damaging variants according to VARITY_R. A similar result was found using scores from our map: we again did not observe a significant difference in the log allele frequency between damaging (map scores below 5th percentile) and tolerated (map scores above >95th percentile) CHK2 variants ($|\Delta \text{ median}| = 0.28$, $p = 0.95$, Wilcoxon rank-sum test) (Figure S11).

Ability of the CHK2 map to distinguish pathogenic from benign variation

We next wished to evaluate the ability of our HiQ CHK2 map to distinguish pathogenic from benign variants. We employed a reference set of 21 pathogenic and 39 benign missense variants (annotated by Invitae using their previously described Sherloc variant classification framework), of which 12 pathogenic and 24 benign had scores in the HiQ map.[32]

We assessed performance of the HiQ map in terms of precision (fraction of variants with scores below a given threshold that are known to be pathogenic) and recall (fraction of known pathogenic variants that received a score below this threshold). Because precision is dependent on the (somewhat arbitrary) balance of pathogenic and benign variants in the reference set, we calculated balanced precision values (precision transformed to the

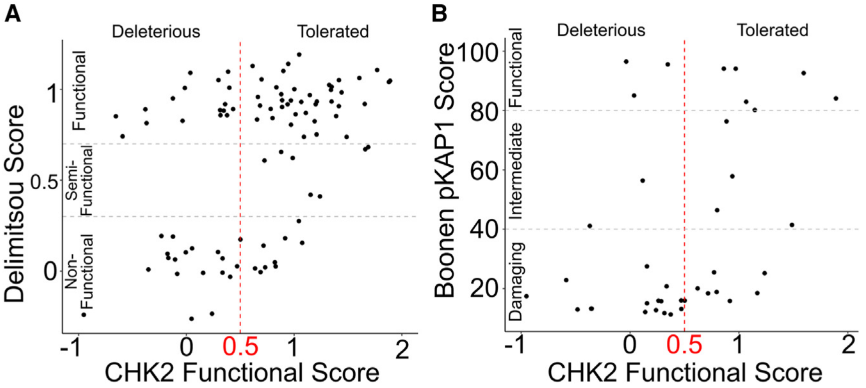

Figure 6. CHK2 variant effect map scores agree with mammalian and yeast functional assays
Scatterplot relating CHK2 variant effect map scores (x axis, indicating map score threshold of 0.5 for reference) to: (A) functional complementation scores for 120 CHK2 missense variants in yeast in the presence of MMS (y axis, indicating map score threshold of 0.5 for reference), indicating previously assigned categories of non-functional, semi-functional, and functional[9]; and (B) measurements of pKAP1 phosphorylation after ionizing radiation in mouse embryonic stem cells (y axis), indicating previously assigned categories of damaging, intermediate, and functional scores.[26]

value that would be expected given a reference set containing 50% pathogenic and 50% benign variants). Our HiQ map achieved an area under the balanced precision vs. recall curve (AUBPRC) of 0.85 and a recall of 50% at a 90% balanced precision (R90BP). Of course, different score thresholds yield different tradeoffs between balanced precision and recall, e.g., the HiQ map detects >70% of pathogenic variants at a balanced precision of >80% (Figure 7A).

Under current ACMG clinical variant interpretation guidelines, evidence from functional assays and computational predictors are treated independently and are therefore complementary. Nevertheless, we were curious to compare the performance of our HiQ map with that of computational predictors, so we investigated the performance of three recent high-performing[53] computational variant effect predictors: VARITY_R, REVEL, and AlphaMissense. All predictors performed well, with AlphaMissense achieving the highest AUBPRC and R90BP (AUBPRC 0.96 and R90BP 0.95, based on the 20 pathogenic and 33 benign variants for which scores were available), followed closely by VARITY_R (AUBPRC 0.94 and R90BP 0.76, based on 21 pathogenic and 39 benign variants) and REVEL (AUBPRC 0.94 and R90BP 0.67, based on 15 pathogenic and 28 benign variants) (Figure S12A). Individually, each predictor was able to detect at least 80% of pathogenic variants at 80% balanced precision. To ensure a fair comparison, we next restricted our reference set to the seven pathogenic and 14 benign variants that were scored by all three predictors and in the HiQ *CHK2* map. For this common variant set, both our HiQ map and REVEL achieved an R90BP of 86%, while VARITY_R and AlphaMissense were nominally better at 100% (Figure S12B). However, no predictor had an R90BP performance that was significantly better than the HiQ map ($p = 1$ for all three comparisons; Fisher's exact test).

To provide our CHK2 map in the most useful form to support clinical variant classification, we derived calibrated measures of evidentiary value for each variant. Using the set of reference variants derived from filtering the original CHK2 scores, we first calculated LLRs of pathogenicity for every variant's functional score by comparing the score distributions observed for pathogenic and benign variants (see material and methods). Conversion from LLR values to the framework of evidence strength descriptors in ACMG/AMP guidelines was performed using an adaptation of the Tavtigian et al. approach[33] (see material and methods). In this calibration LLR scores above 2.5 correspond to "PS3_very strong" evidence of pathogenicity, LLR between 2.5 and 1.3 corresponds to "PS3_strong" evidence, LLR from 1.3 to 0.64 corresponds to "PS3_moderate" evidence, and LLR from 0.64 to 0.32 corresponds to "PS3_supporting" evidence of pathogenicity. Furthermore, LLR scores between −0.32 and −1.32 correspond to "BS3_supporting" evidence of benignity. Using these thresholds, the CHK2 map provided at least a supporting level of evidence (toward either pathogenicity or benignity) for 651 out of 1,519 *CHEK2* VUS in ClinVar (Figure 7B), including 30, 105, 9, and 44 VUS for which the map provided supporting, moderate, strong, and very strong evidence for pathogenicity, respectively, and 463 VUS for which the map provided supporting evidence of benignity (Figure 7C). While the LLR scores and suggested evidence strengths that we provide are based on objective empirical analysis that may inform future clinical variant interpretations, we must provide the important caveat that the variant curation expert panel overseeing guidelines for *CHEK2* variant interpretation has yet to review this calibration procedure, and indeed that current guidelines only allow consideration of yeast-based assays if they are accompanied by functional data from a mammalian cell-based assay (hereditary breast, ovarian and pancreatic cancer VCEP: https://clinicalgenome.org/affiliation/50039/).

To determine whether HiQ variant effect map scores synergize with computational predictors, we calculated CHK2 LLRs using scores from VARITY_R, REVEL, and AlphaMissense (Figure S13). Of the 1,519 VUS in ClinVar, VARITY_R, REVEL, and AlphaMissense provided at least supporting evidence toward benignity for 612, 425, and 647 variants, respectively, and at least supporting evidence toward pathogenicity for 706, 309, and 574 variants,

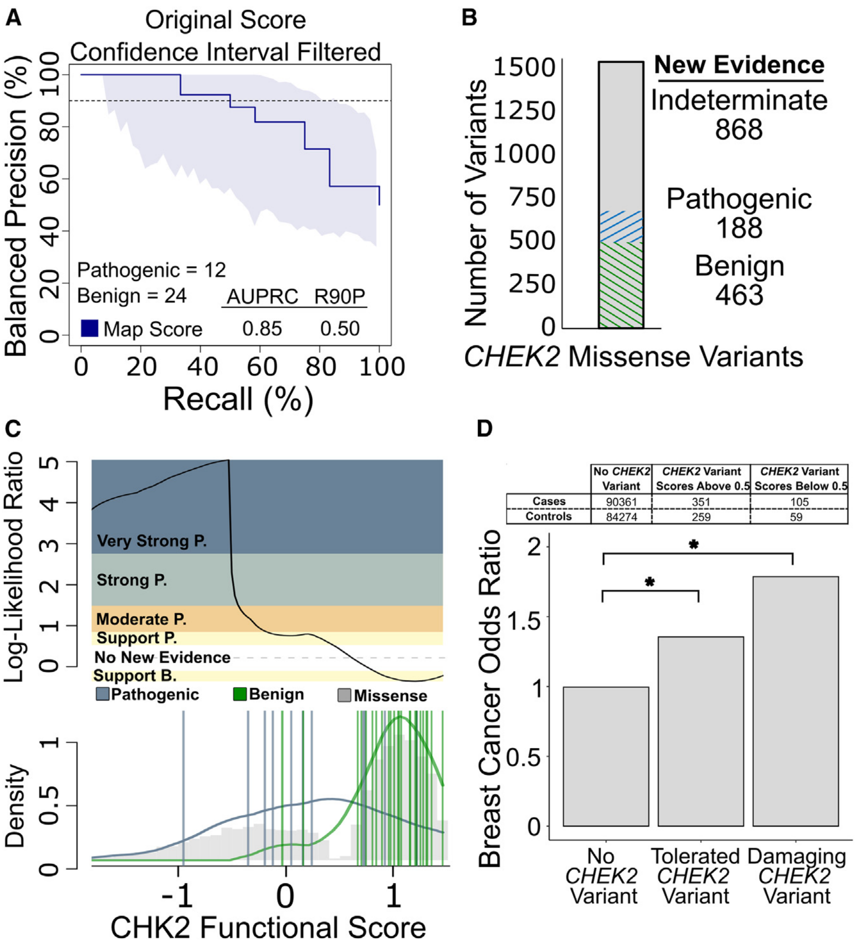

Figure 7. CHK2 functional scores correctly classify clinical variants with known annotations and provide evidence for VUS reclassification

(A) Using CHK2 map scores after confidence-interval filtering and a known set of clinically annotated pathogenic or benign *CHEK2* variants from Invitae, we evaluated balanced precision—defined at each score threshold by the fraction of variants that are pathogenic given a balanced (50% prior probability of pathogenicity) test set—vs. recall (fraction of pathogenic variants captured at this threshold). The horizontal dashed line indicates R90BP with the numerical AUBPRC and R90BP listed in the bottom-right legend. LB/B indicates likely benign and benign, while LP/P means likely pathogenic and pathogenic.

(B) CHK2 functional score ranges were converted into ACMG evidence strengths using log-likelihood ratios (LLRs) and matched against all VUS in ClinVar (as of June 2023). Evidence toward pathogenicity or benignity are split into discrete categories where V. Str, Str., Mod., and Sup. mean very strong, strong, moderate, and supporting, respectively. Sections in blue represent evidence toward pathogenicity and green toward benignity, while gray are VUS where no new evidence is provided. The numbers below each label show how many variants fall into each category.

(C) LLRs of pathogenicity were calculated by comparing the probability distributions of scores for known pathogenic and benign variants in the reference set. The log ratio between likelihood of observing a score in the positive pathogenic reference set (blue) compared to the negative benign reference set (green) was calibrated to ACMG evidence strengths.[33] Probability distributions are overlaid on the gray histogram of CHK2 missense variant scores, with the top panel showing which score ranges correspond to each ACMG evidence strength.

(D) Functional scores were matched with subjects with breast cancer from the BRIDGES and CARRIERS studies, and odds ratios were calculated for individuals without a CHK2 variant, individuals with variants found to be tolerated (score above 0.5) in our functional assay, and individuals with variants that were found to be damaging (score below 0.5). The asterisk indicates a significant difference ($p < 0.05$) according to Fisher's exact test.

respectively. For each predictor, a relatively small fraction of variants (less than 15% of all *CHEK2* VUS) had a computationally predicted score that did not provide evidence toward pathogenicity or benignity. Pairwise comparisons of evidence strengths from the HiQ variant effect map and from each of the predictors showed agreement in terms of evidence toward pathogenicity or benignity. Specifically, of all variants with evidence toward benignity by either the HiQ map or a given predictor, 54% had evidence toward benignity from both HiQ and VARITY_R, 51% from HiQ and REVEL, and 65% from HiQ and AlphaMissense. For all variants with evidence toward pathogenicity from either HiQ or a given predictor, 82% had evidence toward pathogenicity from both HiQ and VARITY_R, 67% from HiQ and REVEL, and 80% from HiQ and AlphaMissense.

We next focused on variants with conflicting evidence (i.e., pathogenic vs. benign rather than pathogenic vs. indeterminate): Of variants with evidence toward benignity in the HiQ map, 34%, 20%, and 29% of variants had evidence toward pathogenicity from VARITY_R, REVEL, and AlphaMissense, respectively. Of variants with evidence toward pathogenicity in the HiQ map, 12%, 17%, and 18% of variants had conflicting evidence toward benignity from VARITY_R, REVEL, and AlphaMissense, respectively. Thus, there are few conflicts with predictors where calibration considers HiQ to provide strong evidence toward pathogenicity and, perhaps not surprisingly, many conflicts when it considers HiQ to offer only weak (supporting) evidence toward benignity.

Finally, we wanted to explore whether our HiQ map has potential to contribute not only to variant interpretation but also to the estimation of breast cancer risk. To this end, we combined two large-scale breast cancer case- and control-cohort sequencing studies,[7,54] comprising a total of 92,713 affected individuals and 86,005 control subjects, to calculate the probability of observing a variant among individuals with breast cancer ("cases") relative to that probability rate in control individuals. Of the 478 *CHEK2* missense variants observed in this dataset, 241 (∼50%) had HiQ map scores, with 190 variants having a

"tolerated" score above 0.5 and 51 variants having a "damaging" score below 0.5. Cohort participants bearing damaging variants had an odds ratio of 1.79, which was significantly higher than the 0.99 odds ratio observed for participants without a *CHEK2* variant (p = 1.10e−3, Fisher's exact test; Figure 7D). Participants with tolerated variants exhibited an odds ratio of 1.35, which was nominally but not significantly lower than that of damaging variants ($p = 0.08$) but still significantly higher than participants without a *CHEK2* variant (p = 2.76e−3). The odds ratios we observed for HiQ-damaging and HiQ-tolerated variants are each similar to the confidence interval for breast cancer odds ratio (1.2–1.77) reported recently for *CHEK2* missense variants in an overlapping cohort.[55] Taken together, these results suggest that the HiQ map has potential to identify risk variants but is less likely to prove useful in identifying neutral variants.

Discussion

The missense variant effect map we generated for CHK2, covering >77% of all possible amino acid changes and 89% of single-nucleotide-substitution-reachable amino acid changes, both recapitulates many known biochemical features of CHK2 and offers a new window on sequence-structure-function relationships and potential clinical applications, albeit with some limitations discussed below.

Correspondence to known biochemical features of CHK2

As expected, our map tended to show low (damaging) functional scores for conserved positions (Figure 3A). Also as expected, solvent-accessible residues appeared more tolerant to variation than non-accessible positions across the entire protein, especially in the FHA domain (amino acids 92–205) and kinase domain (amino acids 212–501) (Figure 3B). Damaging variants were also enriched in known mutational hotspots, with median scores near 0 for substitutions in the reported hotspot motifs APE, DFG, GxGxxG, HRD, and VAIK (Figure 5).

Identifying residues important for CHK2 homodimerization

Variant effect maps can provide new hypotheses about the importance of specific amino acid residues. One approach to this is to combine scores and proximity of residue positions to known protein features. For example, we note that Tyr220 in the N lobe of the kinase domain appeared intolerant to variation (median score 0.08). Although its role is not well described, Tyr220's proximity to Ile221 and Lys224, which are contact sites of the homodimerization interface between CHK2's FHA and kinase domains,[35] coupled with Tyr220's low map scores, suggests that Tyr220 serves to stabilize the FHA/kinase dimer. The FHA-kinase dimerization (FHA-KD) interface is mediated by contacts between hydrophobic residues from both the FHA domain (Gly151, Pro152, Ile157, Tyr159, and Pro182) and the N lobe of the kinase domain (Ile221, Leu226, Leu236, Phe238, Cys243, Lys224, and Lys245).[35] The results from our map support the idea that close proximity of Tyr220 to the hydrophobic core of the FHA-KD interface is important for maintaining favorable contacts and stabilizing the FHA-KD interface.

A similar logic also suggests a role for kinase domain position Ala237 in stabilizing the FHA-KD interface. With a low median score of 0.2, Ala237 is also located at the FHA-KD dimerization interface and is in close proximity to Phe238 which, along with Leu236 and Lys224, is reported to make van der Waals contact with Ile157 in the FHA-KD interface.[35] The substitution of non-polar Ala237 with polar or bulkier amino acids could perturb contacts in the hydrophobic core and thus affect FHA-KD dimer stability.

Several cancer-associated germline variants at the FHA-KD interface[56] are classified as variants of uncertain significance[10] but have low map scores, which suggest their importance. These include: p.Ala247Asp (map score 0.2), which alters a highly conserved residue in the kinase domain in close proximity to Cys243 and Lys245 in the hydrophobic center of the FHA-KD interface, which has previously been reported to affect protein function[57]; p.Pro152Ser (map score 0.2), located at a β5′-β6′ hairpin very close to Ile157 in the hydrophobic core of the interface, which could alter the hairpin conformation and destabilize the dimer by affecting the van der Waals contacts[35]; and p.Arg181Cys (map score 0.3), located at the periphery of the FHA-KD interface, which could affect intramolecular contacts and cause instability at the interface.[35]

During CHK2 activation, a second homodimerization interface is formed between the FHA domains of the two protomers,[58] mediated by van der Waals and hydrogen-bond contacts from three adjacent β strands. The FHA-FHA interaction is mainly centered around the aromatic side chains of Trp97 and Phe202 which make contacts with Trp97 and Phe202, respectively, in the homomeric partner.[35] Substitution at these positions or in positions near the FHA-FHA interface may interrupt van der Waals and hydrogen-bond contacts and further affect the dimerization.[35] Supporting this idea, positions adjacent to Trp97 (93–96) have a median score of 0.0 in our map, indicating abnormal function.

As described in results, the comparison of measured impacts on function with predicted impacts on stability (ΔΔG) can reveal active-site residues or other roles beyond providing stability. For CHK2, this analysis revealed five regions in the FHA domain (positions 93–97, 115–117, 138–144, 164–169, and 191–202) where substitutions impacted function more profoundly than stability (Figure 4). It is interesting to note that in the region 138–144, in which substitutions appear to impact protein function but not protein stability, position Ser140 plays an important role as its autophosphorylation triggers dissociation of the dimer after CHK2 activation.[59] Phosphomimetic

mutations p.Ser140Asp and p.Ser140Glu and phospho-dead mutant p.Ser140Ala exhibited damaging map scores of −1.1, −0.6, and 0.2, respectively. It has been suggested that the WT *CHEK2* allele can allow activation and dissociation of an Ser140-mutant CHK2 partner,[59] potentially explaining how we might observe a functional impact in our (haploid) setting but limited impact on protein stability. Taken together, these results support the previously suggested importance of CHK2 dimerization[26] and that our map provides information about residue function beyond impacts on protein stability.

Inferring residues with roles in CHK2 catalytic activity

Several well-established motifs, including GxGxxG, VAIK, HRD, DFG, and APE, coordinate kinase activation by influencing CHK2 conformation and binding of the substrate and cofactors ATP and magnesium.[36] During kinase activation, after phosphorylation of Thr68 by ATM and subsequent CHK2 homodimerization, the autophosphorylation of residues Thr383 and Thr387 in the activation T loop (positions 371–391) affects several residues corresponding to the above kinase motifs. For example, phosphorylation of Thr383 and Thr387 would be expected to promote electrostatic interaction with the positively charged catalytic HRD residues (345–347) and accompanying conformational change of the DFG residues (368–370).[60] Switching from the inactive "DFG-out" conformation to an active "DFG-in" state helps to create the nucleation site and enable binding of ATP and magnesium.[61] Given the importance of these residues, damaging scores for HRD and DFG were expected; however, several flanking residues surrounding both HRD (343–344 and 348–355) and DFG (364–367 and 371–373) also showed damaging scores, suggesting the possibility that these residues may also be important for kinase activation. Future structural analysis, exploring electrostatic dynamics of the HRD motif as well as the fraction of time spent in the "DFG-in" vs. "DFG-out" conformations for different CHK2 variants, could elucidate residues necessary for catalysis.

Interestingly, the T loop itself showed that substitutions at some (but not all) positions impacted protein function. The activation T loop (positions 371–391) is characterized by two amphipathic α helices spanning positions 377–386 and 392–402.[36] Many positions in the T loop (including 374–380, 384, 388, 389, and 391) displayed surprising tolerance to most substitutions. Indeed, both of the involved alpha helices had a segment displaying tolerance to a range of mutations, including helix-breaking prolines. Discovering tolerated positions at the C-terminal end of the activation T loop was somewhat surprising considering that the conserved APE motif is directly downstream at positions 392–394, and that changing the size and charge of residues adjacent to APE might be expected to impact its ability to stabilize the kinase C lobe during activation. Indeed, a role in stabilizing the C lobe may explain why residues in an alpha helix C-terminal to APE (405–423) were intolerant to substitution. In any case, our results show that many residues in the functionally important and generally conserved T loop are tolerant to substitutions.

Conformational changes in CHK2 following T-loop phosphorylation affect the GxGxxG motif (positions 227–232) in the glycine-rich loop.[36] In the active CHK2 conformation, the GxGxxG motif acts as a flexible clamp, anchoring and orienting ATP for transfer of its phosphate group.[62] Interestingly, residues 220–226 preceding the GxGxxG motif were sensitive to polar substitutions (median score 0.0) but fairly tolerant to hydrophobic variants (median 0.8). Positions C-terminal to the GxGxxG motif may also play a role, based on damaging scores for most substitutions at positions 234, 237, and 238. Substitutions with bulky side chains may reduce flexibility of the glycine-rich loop and limit its ability to anchor ATP.

Conformational changes in CHK2 following T-loop phosphorylation also enable the VAIK (positions 246–249) salt bridge[63] between Lys249 and Glu273 across an intervening disordered loop from position 255 to 268. This stabilizes the glycine-rich loop and thereby enables ATP binding.[36] As expected, the disordered loop is relatively tolerant to variation (median score 0.7). Positions adjacent to the VAIK salt bridge, including 239–245 and 250–252, were surprisingly tolerant to substitutions (median score 0.7).

Further downstream of the VAIK motif, positions 253–258 were somewhat intolerant to variation and appeared to match an enrichment of arginine and lysine residues typical of protein kinase C (PKC) substrates. PKC is known to regulate proliferation[64] and DNA-damage repair.[65] However, the nearby Ser260 phosphorylation site is not conserved and, with a median map score of 1.2, appears to tolerate substitutions. Therefore, damaging scores at positions 253–258 more likely suggest roles for the positions in VAIK salt-bridge formation and ATP binding. Together, our map sheds light on additional positions that may be important for functions of the glycine-rich loop and VAIK salt bridge that work together to stabilize ATP for phosphoryl transfer during catalysis.

Several amino acids in CHK2 have been shown to coordinate ATP binding more directly. Crystal structures of CHK2 complexed with ADP or the ATP analog DBQ identified hydrogen bonds involved in ATP binding at positions Lys249, Glu302, Met304, Glu308, Glu351, Asn352, and Asp368.[36] Our map showed that most of these residues are intolerant to substitutions. The exceptions (Glu302 and Met304, which had median scores of 1.2 and 0.7, respectively) are understandable, given that Glu302 and Met304 form hydrogen bonds via backbone rather than side-chain atoms. Adjacent substitutions (at positions 301, 306, 307, and 309) also exhibited damaging scores, suggesting that these residues may indirectly promote hydrogen bonding by Glu302, Met304, and Glu308. More specifically, substituting small and uncharged residues for bulky amino acids at these positions may affect CHK2 function by steric hindrance, occluding the

hydrogen binding sites needed for ATP binding. For the remaining ATP binding residues (Lys249, Glu351, Asn352, and Asp368), distinguishing between their roles in catalysis and ATP binding is challenging, given that Lys249, Asn352, and Asp368 overlap with the VAIK, HRD+5, and DFG mutational hotspots, respectively.

Potential clinical applications for the CHK2 variant effect map

While loss of CHK2 function has been linked to several cancer types including prostate and colorectal, we focus here on the association with breast cancer. In contrast with the predisposition to breast cancer that is well established for truncating CHK2 variants, the risk associated with missense variants is less often clear.[66] Some CHK2 missense variants, such as p.Ile157Thr and p.Ser428Phe (which received map scores of 0.3 and 1.2, respectively), have been reported to convey a modest (<1.5-fold) elevation of breast cancer risk. By contrast, p.Arg117Gly, which reportedly conveys a >2-fold breast cancer risk that is on par with truncating variants,[67] showed a correspondingly low map score of −1.0. Indeed, a recent analysis showed that CHK2 variants found to be dysfunctional in mammalian cell-based assays (found in 0.5% of individuals with breast cancer) were associated with an increased risk of breast cancer, while functionally normal or mildly dysfunctional variants (found in 2.2% of individuals with breast cancer) were not associated with a clinically relevant increased risk of cancer.[68] Additionally, results from breast cancer case-control cohorts, such as BRIDGES and CARRIERS, suggest that not all *CHEK2* variants confer equivalent risk.[7,54] Thus, a CHK2 variant effect map could help stratify variants by clinical risk and thereby focus the management of clinical resources.

Improved variant interpretation and risk estimation can enable personalized medicine with surveillance and treatment plans that depend on an individual's genotype. For example, heightened screening may be warranted for patients with pathogenic germline *CHEK2* variants. It has been estimated that establishing regular MRI and mammograms based on *CHEK2* genotype could reduce breast cancer mortality by over 50%.[69] Given the suggestion from a recent phase 2 clinical trial that *CHEK2*-associated breast cancers respond less well to poly-adenosine diphosphate ribose polymerase inhibitors, there is the future potential for knowledge of *CHEK2* genotype to inform therapy.[70] Individuals with high-risk *CHEK2* variants and a strong family history of breast cancer may benefit from a pre-emptive or contralateral risk-reducing mastectomy.[66] Together, these results support the potential clinical value of our proactive CHK2 variant effect map.

Limitations/caveats of the map

Our study has several important caveats. First, because of the nature of our assay, our map can only capture those aspects of human CHK2 that are required to rescue the MMS sensitivity phenotype that emerges in a yeast *sml1*Δ strain upon loss of yeast *RAD53*. Thus, variants impacting other functions of human CHK2 may be missed. Post-translational modifications such as phosphorylation and ubiquitination are both important for CHK2 function as a DNA-damage checkpoint.[71] Both CHK2 and its yeast ortholog, Rad53, are phosphorylated after translation, but only CHK2 is ubiquitinated by E3 ligases, which serve to regulate CHK2 protein stability.[72] Indeed, Kleiblova et al.[47] hypothesized that post-translational modifications can influence CHK2 catalytic activity in human cells. Using a mammalian cell-based *in vivo* assay they demonstrated that, when CHK2 undergoes physiological post-translational modifications, the protein has a greater ability to phosphorylate KAP1-Ser473 compared to unmodified recombinant CHK2 tested in an *in vitro* assay. Thus, CHK2 may have (and depend on) post-translational modifying activities in human cells that are not present or required for human CHK2 to functionally replace Rad53 in yeast under our assay conditions.

Furthermore, many CHK2 substrates, including CDC25 A/B/C, the PIK3 kinase, the E2F1 transcription factor, BRCA1/2, and p53, do not have clear yeast counterparts.[4,73] Also, structural differences between Rad53 and CHK2 could explain differences in response to DNA damage. For example, unlike human CHK2, yeast Rad53 contains two FHA domains, which yield differing energetics and dynamics of dimerization (which is in turn required for activation)[74] and also contains two SQ/TQ domains flanking the kinase domain, which may affect the efficiency and context of Thr68 phosphorylation by Mec1/Tel1 (the yeast orthologs of ATR/ATM). Also, the two FHA domains can interact with multiple binding partners during the checkpoint response.[75] For example, during the replication checkpoint response, Rad53 interacts with two binding partners: Dbf4-dependent kinase (a heterodimeric complex of Cdc7 and its regulatory subunit Dbf4) and Rad9. While Dbf4 interacts with the Rad53's N-terminal FHA domain (FHA1), Rad9 interacts preferentially with the C-terminal FHA domain (FHA2). CHK2 contains only one FHA domain so that it may not perfectly functionally rescue Rad53.[4,73] Another limitation is that our yeast assay was conducted at a temperature suitable for optimal yeast growth, which is 30°C instead of 37°C, so that thermodynamic stability of human CHK2 variants expressed in the yeast model may differ from that in human physiological conditions.[76] This could explain why some CHK2 variants exhibiting intermediate functional effects in a mammalian system[26] appear functional in a yeast-based assay.[9]

In our assay, human *CHEK2* cDNA was expressed under the constitutive *ADH1* promoter. Although this promoter is often considered to have moderate strength, we cannot be sure that this does not represent overexpression of the protein, such that some variants that would be mildly dysfunctional at physiological expression levels in a human cell could provide sufficient total activity when overexpressed in yeast. Conversely,

some variants might be toxic to yeast when overexpressed but tolerated at physiological expression levels in human cells.

A further limitation of any cDNA rescue assay is that it will miss some purely non-coding effects of CHK2 coding variants, e.g., on splicing efficiency. Also, observations that a nonsense codon is tolerated in our assay should not be taken as strong evidence that the variant will be tolerated in humans, given that strength of nonsense-mediated decay effects can depend on downstream introns not present in the yeast context.

Despite all of these caveats, assays based on the expression of human cDNA in yeast can provide excellent empirical performance in identifying pathogenic variation.[20,23] Here, we showed that approximately half of CHK2 known pathogenic missense variants could be identified at a stringency achieving 90% balanced precision, i.e., if applied to a test set in which 50% of variants are pathogenic, we would expect that 90% of the map's inferred pathogenic variants would in fact be pathogenic.

A final limitation of this study is that it focused on data from our screen and did not combine evidence from all available independent functional assays. Of particular note is a recent study from McCarthy-Leo et al., which describes another large-scale functional analysis of CHK2 variants using a closely related yeast-based assay.[77] Combining these studies, together with further integration with cancer cohorts and population databases, such as UK BioBank, FinnGen, and All of Us, has the potential to provide yet stronger evidence to enable more definitive classifications of clinical *CHEK2* variants and provide patient-level information for assessing variant-specific risk.[78–80]

Conclusion

This study provides a large resource of *in vitro*-based functional assays of *CHEK2* missense variants, enabling both biochemical insights and representing a proactive assessment of nearly all possible missense variants of *CHEK2*, with potential to enable more rapid and accurate clinical action of *CHEK2* clinical variants.

Data and code availability

All original CHK2 variant functional scores generated in this study are provided in the supplemental .csv file named "Table S5. All CHK2 amino acid scores" and are also available online at MaveDB: https://www.mavedb.org, accession number: urn:mavedb:00001205-a-1.

Acknowledgments

The authors gratefully acknowledge funding from the National Human Genome Research Institute of the National Institutes of Health (NIH/NHGRI) Center of Excellence in Genomic Science (CEGS) Initiative (HG004233 and HG010461), the NIH/NHGRI Impact of Genomic Variation on Function (IGVF) Initiative (UM1HG011989), NIH/NHLBI grant HL164675, NIH/NCI grant R35CA253187, the Canada Excellence Research Chairs, Government of Canada (CERC) Program, the Breast Cancer Research Foundation, and a Canadian Institutes of Health Research Foundation grant (FDN-159926) to F.R.

Declaration of interests

Unrelated to this work, F.P.R. is an investor in Ranomics Inc. and an investor in and advisor for SeqWell Inc. and BioSymetrics Inc. and has accepted grant funding from Alnylam Inc., Biogen Inc., Deep Genomics Inc., and Beam Therapeutics. He is also an investor and advisor in Constantiam Biosciences Inc., which provides related services. A.W., J.R., and B.J. are employed by and invested in Invitae. F.J.C. has received research support from GRAIL and consulting funding from AstraZeneca.

Supplemental information

Supplemental information can be found online at https://doi.org/10.1016/j.ajhg.2024.10.013.

Received: February 29, 2024
Accepted: October 22, 2024
Published: December 5, 2024

References

1. Stolz, A., Ertych, N., and Bastians, H. (2011). Tumor suppressor CHK2: regulator of DNA damage response and mediator of chromosomal stability. Clin. Cancer Res. *17*, 401–405.
2. Gunjan, A., and Verreault, A. (2003). A Rad53 kinase-dependent surveillance mechanism that regulates histone protein levels in S. cerevisiae. Cell *115*, 537–549.
3. Emili, A., Schieltz, D.M., Yates, J.R., 3rd, and Hartwell, L.H. (2001). Dynamic interaction of DNA damage checkpoint protein Rad53 with chromatin assembly factor Asf1. Mol. Cell *7*, 13–20.
4. Apostolou, P., and Papasotiriou, I. (2017). Current perspectives on CHEK2 mutations in breast cancer. Breast Cancer *9*, 331–335.
5. Bell, D.W., Varley, J.M., Szydlo, T.E., Kang, D.H., Wahrer, D.C., Shannon, K.E., Lubratovich, M., Verselis, S.J., Isselbacher, K.J., Fraumeni, J.F., et al. (1999). Heterozygous germ line hCHK2 mutations in Li-Fraumeni syndrome. Science *286*, 2528–2531.
6. Maxwell, K.N., Hart, S.N., Vijai, J., Schrader, K.A., Slavin, T.P., Thomas, T., Wubbenhorst, B., Ravichandran, V., Moore, R.M., Hu, C., et al. (2016). Evaluation of ACMG-Guideline-Based Variant Classification of Cancer Susceptibility and Non-Cancer-Associated Genes in Families Affected by Breast Cancer. Am. J. Hum. Genet. *98*, 801–817.
7. Breast Cancer Association Consortium, Dorling, L., Carvalho, S., Allen, J., González-Neira, A., Luccarini, C., Wahlström, C., Pooley, K.A., Parsons, M.T., Fortuno, C., et al. (2021). Breast Cancer Risk Genes - Association Analysis in More than 113,000 Women. N. Engl. J. Med. *384*, 428–439.
8. Cybulski, C., Górski, B., Huzarski, T., Masojć, B., Mierzejewski, M., Debniak, T., Teodorczyk, U., Byrski, T., Gronwald, J., Matyjasik, J., et al. (2004). CHEK2 is a multiorgan cancer susceptibility gene. Am. J. Hum. Genet. *75*, 1131–1135.
9. Delimitsou, A., Fostira, F., Kalfakakou, D., Apostolou, P., Konstantopoulou, I., Kroupis, C., Papavassiliou, A.G., Kleibl, Z.,

Stratikos, E., Voutsinas, G.E., and Yannoukakos, D. (2019). Functional characterization of CHEK2 variants in a Saccharomyces cerevisiae system. Hum. Mutat. *40*, 631–648.
10. Landrum, M.J., Lee, J.M., Benson, M., Brown, G.R., Chao, C., Chitipiralla, S., Gu, B., Hart, J., Hoffman, D., Jang, W., et al. (2018). ClinVar: improving access to variant interpretations and supporting evidence. Nucleic Acids Res. *46*, D1062–D1067.
11. Weile, J., Sun, S., Cote, A.G., Knapp, J., Verby, M., Mellor, J.C., Wu, Y., Pons, C., Wong, C., van Lieshout, N., et al. (2017). A framework for exhaustively mapping functional missense variants. Mol. Syst. Biol. *13*, 957.
12. Tabet, D., Parikh, V., Mali, P., Roth, F.P., and Claussnitzer, M. (2022). Scalable Functional Assays for the Interpretation of Human Genetic Variation. Annu. Rev. Genet. *56*, 441–465.
13. Fayer, S., Horton, C., Dines, J.N., Rubin, A.F., Richardson, M.E., McGoldrick, K., Hernandez, F., Pesaran, T., Karam, R., Shirts, B.H., et al. (2021). Closing the gap: Systematic integration of multiplexed functional data resolves variants of uncertain significance in BRCA1, TP53, and PTEN. Am. J. Hum. Genet. *108*, 2248–2258.
14. Scott, A., Hernandez, F., Chamberlin, A., Smith, C., Karam, R., and Kitzman, J.O. (2022). Saturation-scale functional evidence supports clinical variant interpretation in Lynch syndrome. Genome Biol. *23*, 266.
15. Zhou, B.B., and Elledge, S.J. (2000). The DNA damage response: putting checkpoints in perspective. Nature *408*, 433–439.
16. Matsuoka, S., Huang, M., and Elledge, S.J. (1998). Linkage of ATM to cell cycle regulation by the Chk2 protein kinase. Science *282*, 1893–1897.
17. Weile, J., Kishore, N., Sun, S., Maaieh, R., Verby, M., Li, R., Fotiadou, I., Kitaygorodsky, J., Wu, Y., Holenstein, A., et al. (2021). Shifting landscapes of human MTHFR missense-variant effects. Am. J. Hum. Genet. *108*, 1283–1300.
18. Sun, S., Yang, F., Tan, G., Costanzo, M., Oughtred, R., Hirschman, J., Theesfeld, C.L., Bansal, P., Sahni, N., Yi, S., et al. (2016). An extended set of yeast-based functional assays accurately identifies human disease mutations. Genome Res. *26*, 670–680.
19. Weile, J., and Roth, F.P. (2018). Multiplexed assays of variant effects contribute to a growing genotype–phenotype atlas. Hum. Genet. *137*, 665–678.
20. Sun, S., Weile, J., Verby, M., Wu, Y., Wang, Y., Cote, A.G., Fotiadou, I., Kitaygorodsky, J., Vidal, M., Rine, J., et al. (2020). A proactive genotype-to-patient-phenotype map for cystathionine beta-synthase. Genome Med. *12*, 13.
21. Roeb, W., Higgins, J., and King, M.-C. (2012). Response to DNA damage of CHEK2 missense mutations in familial breast cancer. Hum. Mol. Genet. *21*, 2738–2744.
22. Fowler, D.M., Araya, C.L., Fleishman, S.J., Kellogg, E.H., Stephany, J.J., Baker, D., and Fields, S. (2010). High-resolution mapping of protein sequence-function relationships. Nat. Methods *7*, 741–746.
23. Giaever, G., and Nislow, C. (2014). The yeast deletion collection: a decade of functional genomics. Genetics *197*, 451–465.
24. Yang, X., Boehm, J.S., Yang, X., Salehi-Ashtiani, K., Hao, T., Shen, Y., Lubonja, R., Thomas, S.R., Alkan, O., Bhimdi, T., et al. (2011). A public genome-scale lentiviral expression library of human ORFs. Nat. Methods *8*, 659–661.
25. Wang, N., Ding, H., Liu, C., Li, X., Wei, L., Yu, J., Liu, M., Ying, M., Gao, W., Jiang, H., and Wang, Y. (2015). A novel recurrent CHEK2 Y390C mutation identified in high-risk Chinese breast cancer patients impairs its activity and is associated with increased breast cancer risk. Oncogene *34*, 5198–5205.
26. Boonen, R.A.C.M., Wiegant, W.W., Celosse, N., Vroling, B., Heijl, S., Kote-Jarai, Z., Mijuskovic, M., Cristea, S., Solleveld-Westerink, N., van Wezel, T., et al. (2022). Functional Analysis Identifies Damaging CHEK2 Missense Variants Associated with Increased Cancer Risk. Cancer Res. *82*, 615–631.
27. Langmead, B., and Salzberg, S.L. (2012). Fast gapped-read alignment with Bowtie 2. Nat. Methods *9*, 357–359.
28. Baldi, P., and Long, A.D. (2001). A Bayesian framework for the analysis of microarray expression data: regularized t -test and statistical inferences of gene changes. Bioinformatics *17*, 509–519.
29. Jumper, J., Evans, R., Pritzel, A., Green, T., Figurnov, M., Ronneberger, O., Tunyasuvunakool, K., Bates, R., Žídek, A., Potapenko, A., et al. (2021). Highly accurate protein structure prediction with AlphaFold. Nature *596*, 583–589.
30. Mitternacht, S. (2016). FreeSASA: An open source C library for solvent accessible surface area calculations. F1000Res. *5*, 189.
31. Montanucci, L., Capriotti, E., Frank, Y., Ben-Tal, N., and Fariselli, P. (2019). DDGun: an untrained method for the prediction of protein stability changes upon single and multiple point variations. BMC Bioinf. *20*, 335.
32. Nykamp, K., Anderson, M., Powers, M., Garcia, J., Herrera, B., Ho, Y.-Y., Kobayashi, Y., Patil, N., Thusberg, J., Westbrook, M., et al. (2017). Sherloc: a comprehensive refinement of the ACMG-AMP variant classification criteria. Genet. Med. *19*, 1105–1117.
33. Tavtigian, S.V., Greenblatt, M.S., Harrison, S.M., Nussbaum, R.L., Prabhu, S.A., Boucher, K.M., Biesecker, L.G.; and ClinGen Sequence Variant Interpretation Working Group ClinGen SVI (2018). Modeling the ACMG/AMP variant classification guidelines as a Bayesian classification framework. Genet. Med. *20*, 1054–1060.
34. van Loggerenberg, W., Sowlati-Hashjin, S., Weile, J., Hamilton, R., Chawla, A., Sheykhkarimli, D., Gebbia, M., Kishore, N., Frésard, L., Mustajoki, S., et al. (2023). Systematically testing human HMBS missense variants to reveal mechanism and pathogenic variation. Am. J. Hum. Genet. *110*, 1769–1786.
35. Cai, Z., Chehab, N.H., and Pavletich, N.P. (2009). Structure and activation mechanism of the CHK2 DNA damage checkpoint kinase. Mol. Cell *35*, 818–829.
36. Oliver, A.W., Paul, A., Boxall, K.J., Barrie, S.E., Aherne, G.W., Garrett, M.D., Mittnacht, S., and Pearl, L.H. (2006). Trans-activation of the DNA-damage signalling protein kinase Chk2 by T-loop exchange. EMBO J. *25*, 3179–3190.
37. Krogh, B.O., and Symington, L.S. (2004). Recombination proteins in yeast. Annu. Rev. Genet. *38*, 233–271.
38. Lundin, C., North, M., Erixon, K., Walters, K., Jenssen, D., Goldman, A.S.H., and Helleday, T. (2005). Methyl methanesulfonate (MMS) produces heat-labile DNA damage but no detectable in vivo DNA double-strand breaks. Nucleic Acids Res. *33*, 3799–3811.
39. Zhao, X., Georgieva, B., Chabes, A., Domkin, V., Ippel, J.H., Schleucher, J., Wijmenga, S., Thelander, L., and Rothstein, R. (2000). Mutational and structural analyses of the ribonucleotide reductase inhibitor Sml1 define its Rnr1 interaction domain whose inactivation allows suppression of mec1 and rad53 lethality. Mol. Cell Biol. *20*, 9076–9083.

40. Zhao, X., Chabes, A., Domkin, V., Thelander, L., and Rothstein, R. (2001). The ribonucleotide reductase inhibitor Sml1 is a new target of the Mec1/Rad53 kinase cascade during growth and in response to DNA damage. EMBO J. *20*, 3544–3553.
41. Montanucci, L., Capriotti, E., Birolo, G., Benevenuta, S., Pancotti, C., Lal, D., and Fariselli, P. (2022). DDGun: an untrained predictor of protein stability changes upon amino acid variants. Nucleic Acids Res. *50*, W222–W227.
42. Høie, M.H., Cagiada, M., Beck Frederiksen, A.H., Stein, A., and Lindorff-Larsen, K. (2022). Predicting and interpreting large-scale mutagenesis data using analyses of protein stability and conservation. Cell Rep. *38*, 110207.
43. Cagiada, M., Johansson, K.E., Valanciute, A., Nielsen, S.V., Hartmann-Petersen, R., Yang, J.J., Fowler, D.M., Stein, A., and Lindorff-Larsen, K. (2021). Understanding the Origins of Loss of Protein Function by Analyzing the Effects of Thousands of Variants on Activity and Abundance. Mol. Biol. Evol. *38*, 3235–3246.
44. Schwarz, J.K., Lovly, C.M., and Piwnica-Worms, H. (2003). Regulation of the Chk2 protein kinase by oligomerization-mediated cis- and trans-phosphorylation. Mol. Cancer Res. *1*, 598–609.
45. Zannini, L., Delia, D., and Buscemi, G. (2014). CHK2 kinase in the DNA damage response and beyond. J. Mol. Cell Biol. *6*, 442–457.
46. Hudson, A.M., Stephenson, N.L., Li, C., Trotter, E., Fletcher, A.J., Katona, G., Bieniasz-Krzywiec, P., Howell, M., Wirth, C., Furney, S., et al. (2018). Truncation- and motif-based pan-cancer analysis reveals tumor-suppressing kinases. Sci. Signal. *11*, eaan6776.
47. Kleiblova, P., Stolarova, L., Krizova, K., Lhota, F., Hojny, J., Zemankova, P., Havranek, O., Vocka, M., Cerna, M., Lhotova, K., et al. (2019). Identification of deleterious germline *CHEK2* mutations and their association with breast and ovarian cancer. Int. J. Cancer *145*, 1782–1797.
48. Bell, D.W., Kim, S.H., Godwin, A.K., Schiripo, T.A., Harris, P.L., Haserlat, S.M., Wahrer, D.C.R., Haiman, C.A., Daly, M.B., Niendorf, K.B., et al. (2007). Genetic and functional analysis of CHEK2 (CHK2) variants in multiethnic cohorts. Int. J. Cancer *121*, 2661–2667.
49. Wu, Q., Fang, C., Wang, X., Huang, S., and Weng, G. (2023). CHEK2 is a potential prognostic biomarker associated with immune infiltration in clear cell renal cell carcinoma. Sci. Rep. *13*, 21928.
50. Hsieh, C.-C., Hsu, S.-H., Lin, C.-Y., Liaw, H.-J., Li, T.-W., Jiang, K.-Y., Chiang, N.-J., Chen, S.-H., Lin, B.-W., Chen, P.-C., et al. (2022). CHK2 activation contributes to the development of oxaliplatin resistance in colorectal cancer. Br. J. Cancer *127*, 1615–1628.
51. Weile, J., Sun, S., Cote, A.G., Knapp, J., Verby, M., Mellor, J.C., Wu, Y., Pons, C., Wong, C., van Lieshout, N., et al. (2017). A framework for exhaustively mapping functional missense variants. Mol. Syst. Biol. *13*, 957.
52. Chen, S., Francioli, L.C., Goodrich, J.K., Collins, R.L., Kanai, M., Wang, Q., Alföldi, J., Watts, N.A., Vittal, C., Gauthier, L.D., et al. (2024). Author Correction: A genomic mutational constraint map using variation in 76,156 human genomes. Nature *626*, E1.
53. Livesey, B.J., and Marsh, J.A. (2023). Updated benchmarking of variant effect predictors using deep mutational scanning. Mol. Syst. Biol. *19*, e11474.
54. Hu, C., Hart, S.N., Gnanaolivu, R., Huang, H., Lee, K.Y., Na, J., Gao, C., Lilyquist, J., Yadav, S., Boddicker, N.J., et al. (2021). A Population-Based Study of Genes Previously Implicated in Breast Cancer. N. Engl. J. Med. *384*, 440–451.
55. Mukhtar, T.K., Wilcox, N., Dennis, J., Yang, X., Naven, M., Mavaddat, N., Perry, J.R.B., Gardner, E.J., and Easton, D.F. (2024). Protein-truncating and rare missense variants in*ATM*and*CHEK2*and associations with cancer in UK Biobank whole-exome sequenced data.
56. Bartek, J., and Lukas, J. (2003). Chk1 and Chk2 kinases in checkpoint control and cancer. Cancer Cell *3*, 421–429.
57. Zoppoli, G., Solier, S., Reinhold, W.C., Liu, H., Connelly, J.W., Jr., Monks, A., Shoemaker, R.H., Abaan, O.D., Davis, S.R., Meltzer, P.S., et al. (2012). CHEK2 genomic and proteomic analyses reveal genetic inactivation or endogenous activation across the 60 cell lines of the US National Cancer Institute. Oncogene *31*, 403–418.
58. Stolarova, L., Kleiblova, P., Janatova, M., Soukupova, J., Zemankova, P., Macurek, L., and Kleibl, Z. (2020). CHEK2 Germline Variants in Cancer Predisposition: Stalemate Rather than Checkmate. Cells *9*, 2675.
59. Li, J., Taylor, I.A., Lloyd, J., Clapperton, J.A., Howell, S., MacMillan, D., and Smerdon, S.J. (2008). Chk2 oligomerization studied by phosphopeptide ligation: implications for regulation and phosphodependent interactions. J. Biol. Chem. *283*, 36019–36030.
60. Krupa, A., Preethi, G., and Srinivasan, N. (2004). Structural modes of stabilization of permissive phosphorylation sites in protein kinases: distinct strategies in Ser/Thr and Tyr kinases. J. Mol. Biol. *339*, 1025–1039.
61. Möbitz, H. (2015). The ABC of protein kinase conformations. Biochim. Biophys. Acta *1854*, 1555–1566.
62. Steinberg, S.F. (2018). Post-translational modifications at the ATP-positioning G-loop that regulate protein kinase activity. Pharmacol. Res. *135*, 181–187.
63. McSkimming, D.I., Rasheed, K., and Kannan, N. (2017). Classifying kinase conformations using a machine learning approach. BMC Bioinf. *18*, 86.
64. Musashi, M., Ota, S., and Shiroshita, N. (2000). The role of protein kinase C isoforms in cell proliferation and apoptosis. Int. J. Hematol. *72*, 12–19.
65. Soriano-Carot, M., Quilis, I., Bañó, M.C., and Igual, J.C. (2014). Protein kinase C controls activation of the DNA integrity checkpoint. Nucleic Acids Res. *42*, 7084–7095.
66. Hanson, H., Astiazaran-Symonds, E., Amendola, L.M., Balmaña, J., Foulkes, W.D., James, P., Klugman, S., Ngeow, J., Schmutzler, R., Voian, N., et al. (2023). Management of individuals with germline pathogenic/likely pathogenic variants in CHEK2: A clinical practice resource of the American College of Medical Genetics and Genomics (ACMG). Genet. Med. *25*, 100870.
67. Southey, M.C., Goldgar, D.E., Winqvist, R., Pylkäs, K., Couch, F., Tischkowitz, M., Foulkes, W.D., Dennis, J., Michailidou, K., van Rensburg, E.J., et al. (2016). PALB2, CHEK2 and ATM rare variants and cancer risk: data from COGS. J. Med. Genet. *53*, 800–811.
68. Stolarova, L., Kleiblova, P., Zemankova, P., Stastna, B., Janatova, M., Soukupova, J., Achatz, M.I., Ambrosone, C., Apostolou, P., Arun, B.K., et al. (2023). ENIGMA CHEK2gether Project: A Comprehensive Study Identifies Functionally Impaired CHEK2 Germline Missense Variants Associated with Increased Breast Cancer Risk. Clin. Cancer Res. *29*, 3037–3050.

69. Lowry, K.P., Geuzinge, H.A., Stout, N.K., Alagoz, O., Hampton, J., Kerlikowske, K., de Koning, H.J., Miglioretti, D.L., van Ravesteyn, N.T., Schechter, C., et al. (2022). Breast Cancer Screening Strategies for Women With ATM, CHEK2, and PALB2 Pathogenic Variants: A Comparative Modeling Analysis. JAMA Oncol. *8*, 587–596.
70. Tung, N.M., Boughey, J.C., Pierce, L.J., Robson, M.E., Bedrosian, I., Dietz, J.R., Dragun, A., Gelpi, J.B., Hofstatter, E.W., Isaacs, C.J., et al. (2020). Management of Hereditary Breast Cancer: American Society of Clinical Oncology, American Society for Radiation Oncology, and Society of Surgical Oncology Guideline. J. Clin. Oncol. *38*, 2080–2106.
71. Bohgaki, M., Hakem, A., Halaby, M.J., Bohgaki, T., Li, Q., Bissey, P.A., Shloush, J., Kislinger, T., Sanchez, O., Sheng, Y., and Hakem, R. (2013). The E3 ligase PIRH2 polyubiquitylates CHK2 and regulates its turnover. Cell Death Differ. *20*, 812–822.
72. Bohgaki, M., Bohgaki, T., El Ghamrasni, S., Srikumar, T., Maire, G., Panier, S., Fradet-Turcotte, A., Stewart, G.S., Raught, B., Hakem, A., and Hakem, R. (2013). RNF168 ubiquitylates 53BP1 and controls its response to DNA double-strand breaks. Proc. Natl. Acad. Sci. USA *110*, 20982–20987.
73. Nevanlinna, H., and Bartek, J. (2006). The CHEK2 gene and inherited breast cancer susceptibility. Oncogene *25*, 5912–5919.
74. Wybenga-Groot, L.E., Ho, C.S., Sweeney, F.D., Ceccarelli, D.F., McGlade, C.J., Durocher, D., and Sicheri, F. (2014). Structural basis of Rad53 kinase activation by dimerization and activation segment exchange. Cell. Signal. *26*, 1825–1836.
75. Almawi, A.W., Matthews, L.A., Duncker, B.P., Guarné, A., Myrox, P., Myrox, P., Boulton, S., Lai, C., Moraes, T., Melacini, G., and Ghirlando, R. (2016). “AND” logic gates at work: Crystal structure of Rad53 bound to Dbf4 and Cdc7. Sci. Rep. *6*, 34237.
76. Boonen, R.A.C.M., Vreeswijk, M.P.G., and van Attikum, H. (2022). CHEK2 variants: linking functional impact to cancer risk. Trends Cancer *8*, 759–770.
77. McCarthy-Leo, C.E., Brush, G.S., Pique-Regi, R., Luca, F., Tainsky, M.A., and Finley, R.L., Jr. (2024). Comprehensive analysis of the functional impact of single nucleotide variants of human CHEK2. PLoS Genet. *20*, e1011375.
78. Kurki, M.I., Karjalainen, J., Palta, P., Sipilä, T.P., Kristiansson, K., Donner, K.M., Reeve, M.P., Laivuori, H., Aavikko, M., Kaunisto, M.A., et al. (2023). Author Correction: FinnGen provides genetic insights from a well-phenotyped isolated population. Nature *615*, E19.
79. Sudlow, C., Gallacher, J., Allen, N., Beral, V., Burton, P., Danesh, J., Downey, P., Elliott, P., Green, J., Landray, M., et al. (2015). UK biobank: an open access resource for identifying the causes of a wide range of complex diseases of middle and old age. PLoS Med. *12*, e1001779.
80. All of Us Research Program Investigators, Denny, J.C., Rutter, J.L., Goldstein, D.B., Philippakis, A., Smoller, J.W., Jenkins, G., and Dishman, E. (2019). The “All of Us” Research Program. N. Engl. J. Med. *381*, 668–676.

The American Journal of Human Genetics, Volume *111*

Supplemental information

A missense variant effect map

for the human tumor-suppressor protein CHK2

Marinella Gebbia, Daniel Zimmerman, Rosanna Jiang, Maria Nguyen, Jochen Weile, Roujia Li, Michelle Gavac, Nishka Kishore, Song Sun, Rick A. Boonen, Rayna Hamilton, Jennifer N. Dines, Alexander Wahl, Jason Reuter, Britt Johnson, Douglas M. Fowler, Fergus J. Couch, Haico van Attikum, and Frederick P. Roth

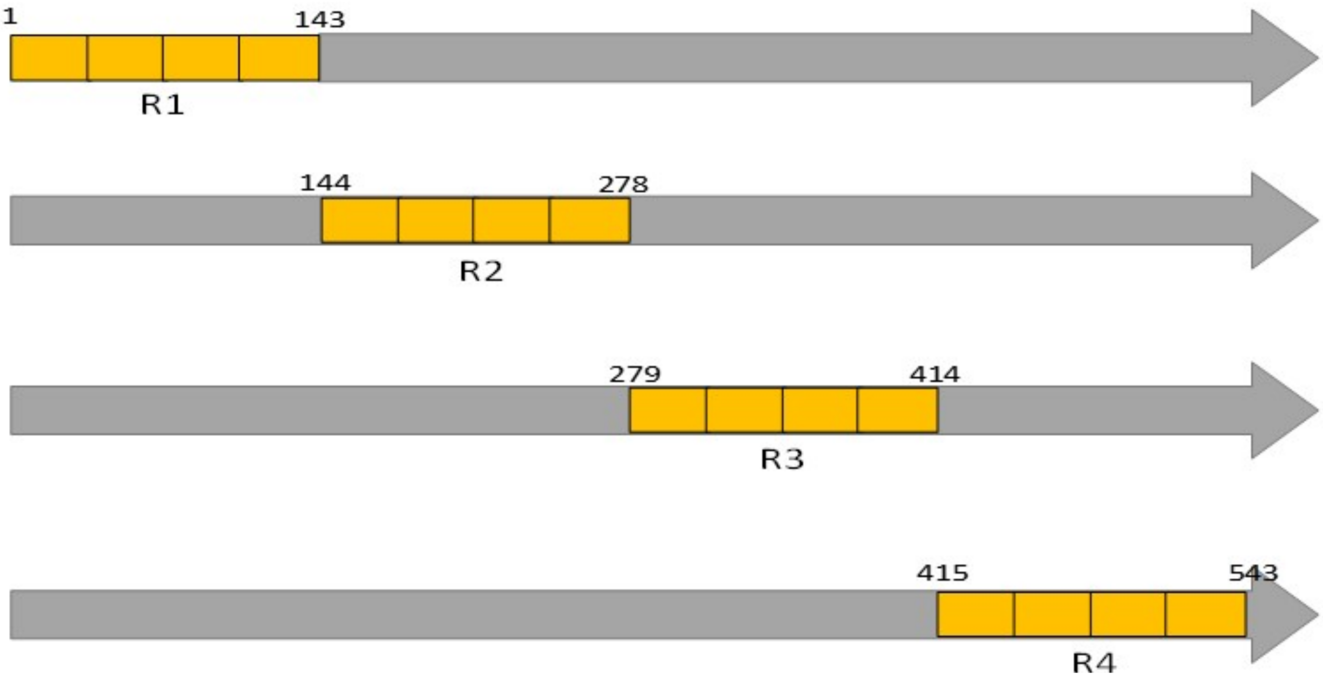

Figure S1. Mutagenized region of CHK2. We defined four regions of CHK2, corresponding to an average length of 150 AA each. Mutagenesis was targeted to each region in turn to generate four mutagenized libraries. For each region, we designed four sequencing tiles for the purpose of sequencing to estimate mutational frequencies before and after selection. The DMS-tileseq framework was followed separately for each regionally-mutagenized library.

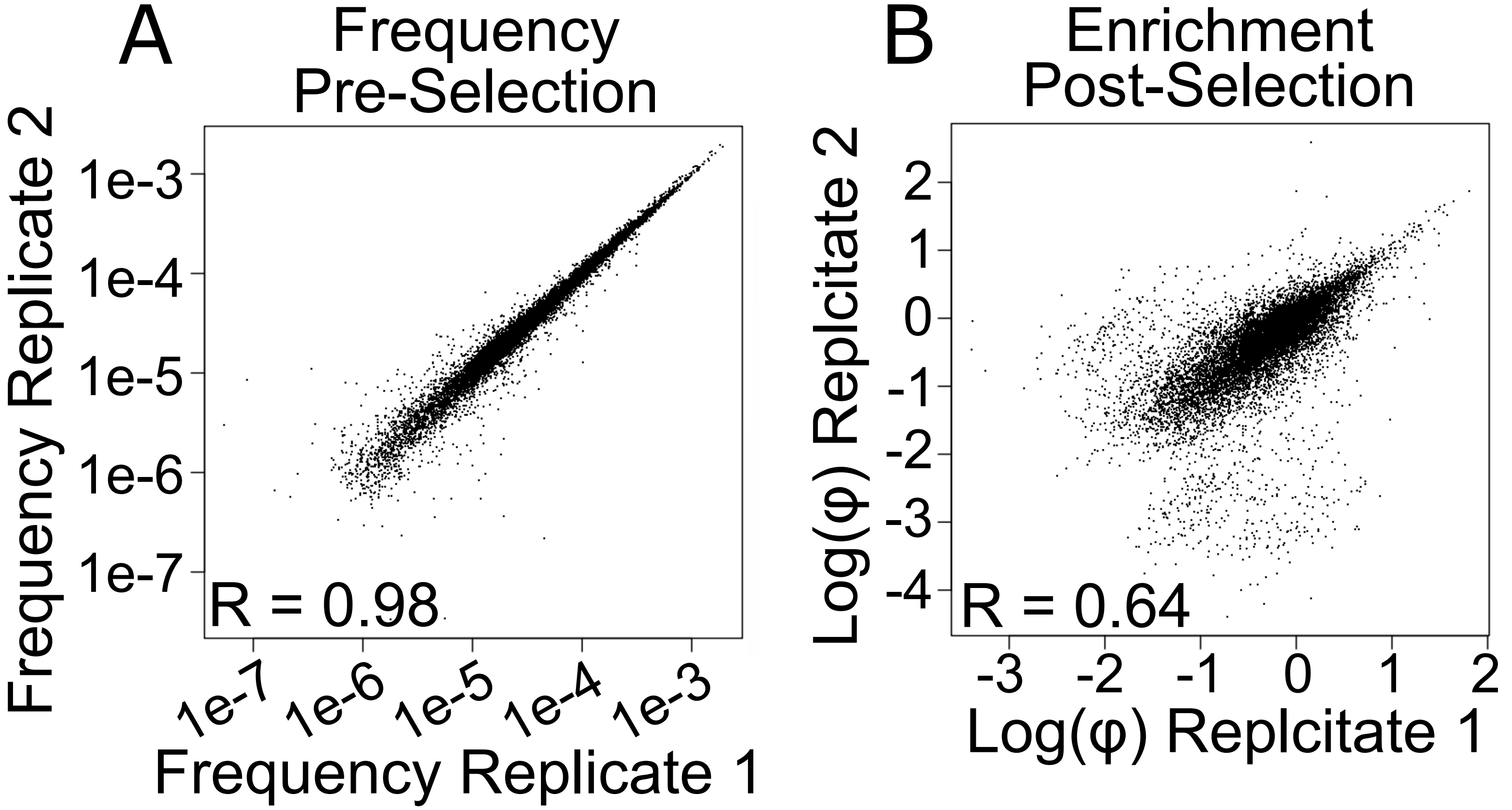

Figure S2. Replicate correlation across sequencing and selection. A To calculate the frequency of each CHEK2 variant in the pool and estimate error rates associated with tiling PCR and sequencing, two independent sequencing libraries from the non-select condition (baseline media without MMS) were prepared (see "quantifying variant abundance" section in Methods). Correlation of variant frequency between replicate pools was assessed by Pearson correlation. **B** Two independent experimental replicates from the selective condition (media with 0.007%MMS) were performed and correlation of variant-specific log(φ) enrichment ratios across replicates (comparing the frequency of each variant in the non-select condition to the select condition) was assessed by Pearson correlation.

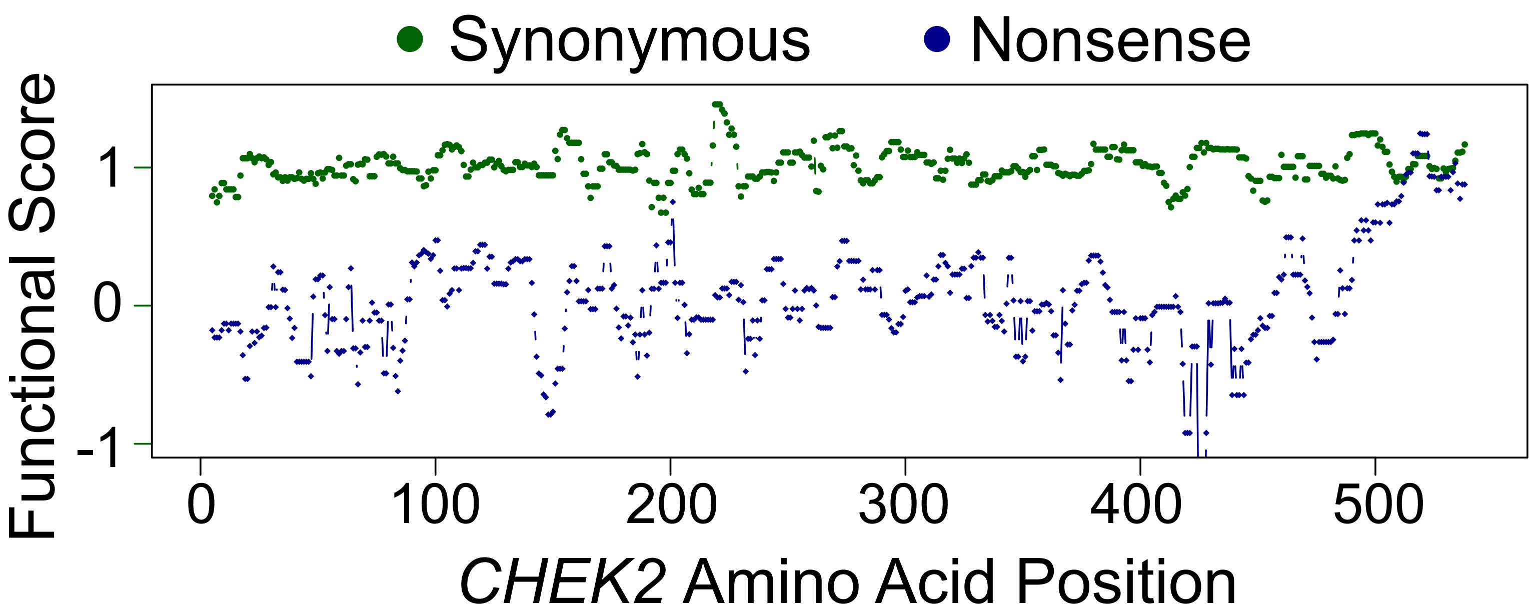

Figure S3. CHEK2 MMS assay effectively separates synonymous and nonsense variants. A moving window analysis of synonymous and nonsense scores along CHK2 positions was performed to assess the experiment's ability to separate neutral and loss-of-function variants. For each position evaluated, a window of 10 residues centered on that position was captured and median synonymous (green) and median nonsense (blue) scores were plotted. The majority of scores ranged from -1 to 1 with the exception of positions 425 to 427 for nonsense variants where scores dropped to between -2 to -3 (not shown).

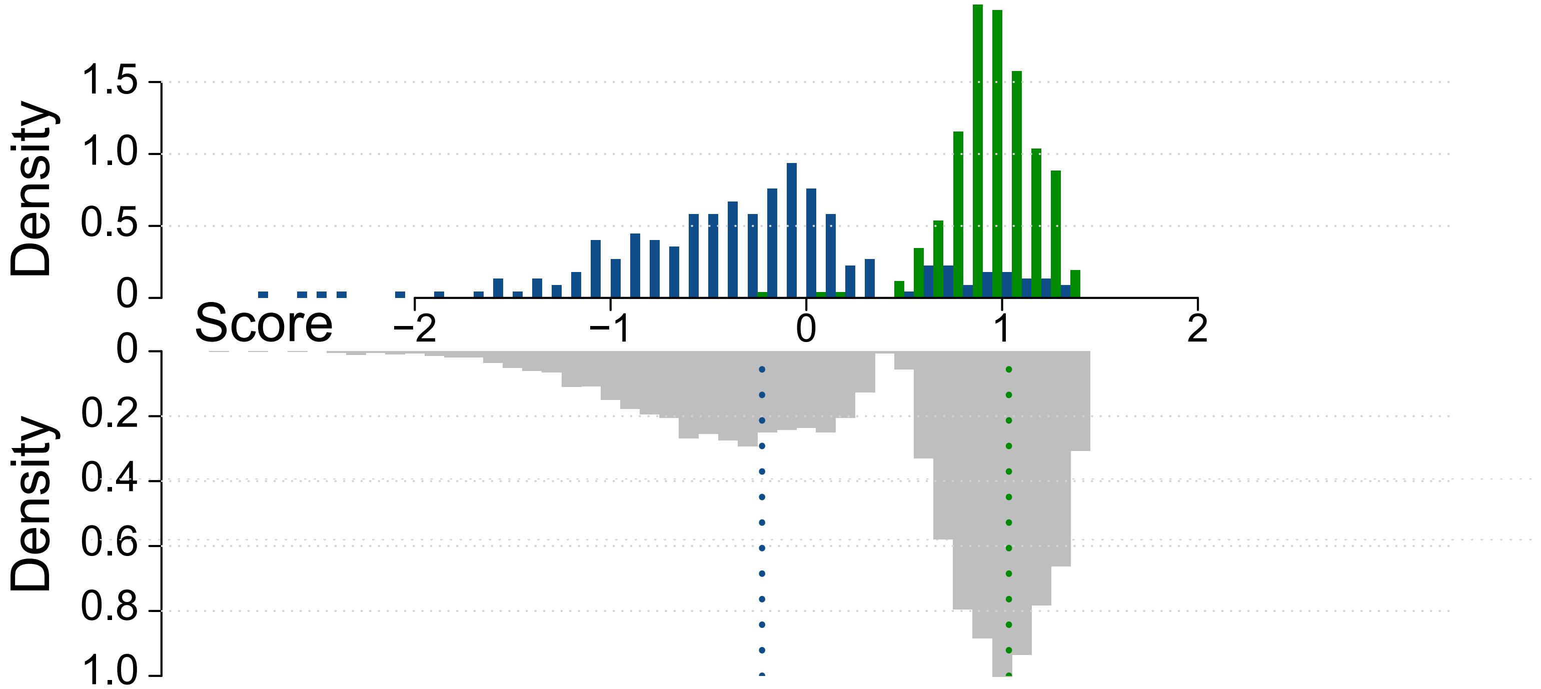

Figure S4. Distribution of CHK2 scores after confidence interval filtering and removal of hyper-complementing variants. Functional scores for synonymous, nonsense, and missense variants from the original map were plotted as a histogram with scores on the x-axis and density on the y-axis. Synonymous variants are shown in green, nonsense in blue, and missense in grey. The median value for each variant type is shown as dotted vertical lines that match the colour code above. Confidence interval filtering and removal of hypercomplementing variants as applied to the original functional as described in the methods.

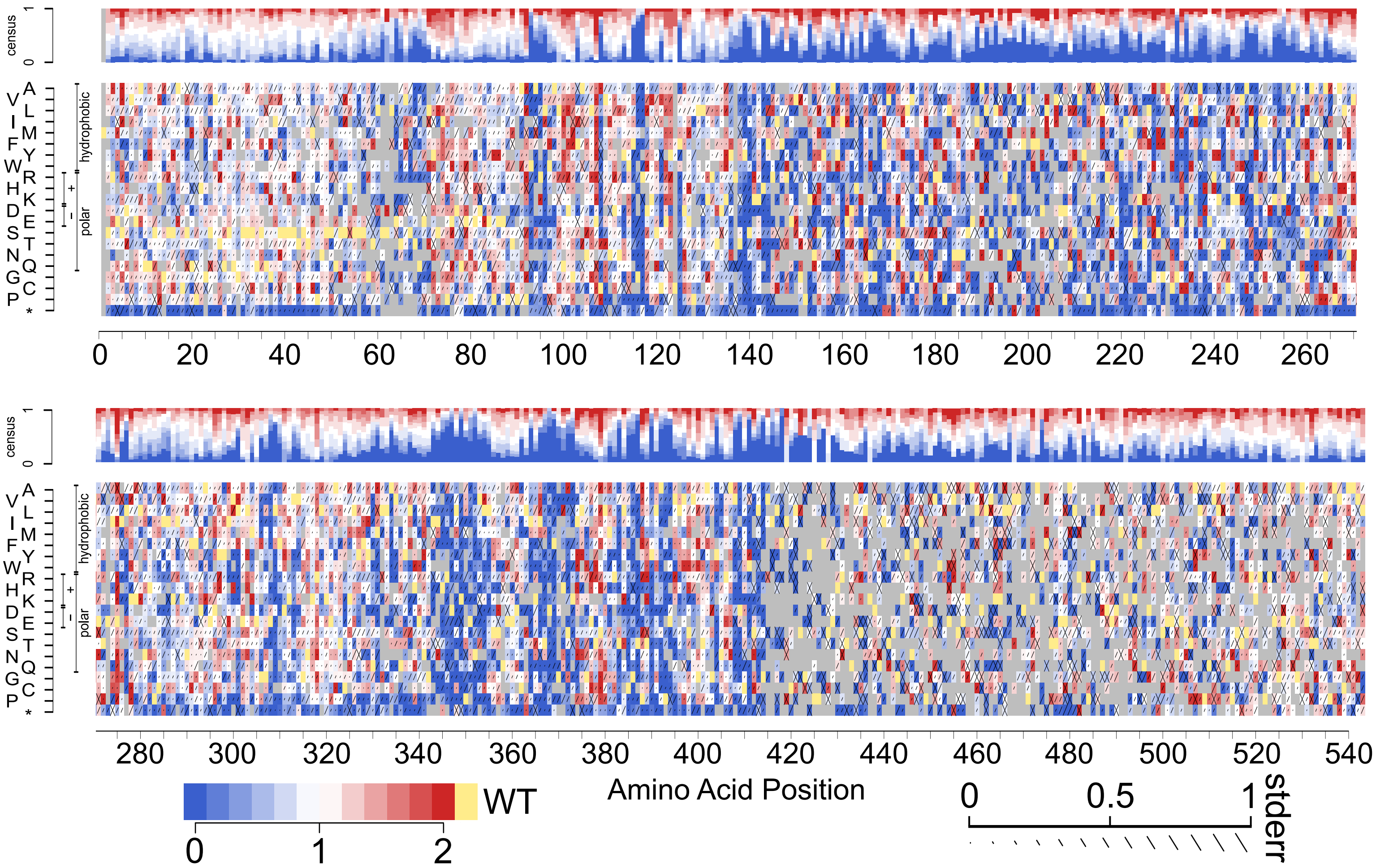

Figure S5. Full length CHK2 variant effect map.

Functional scores derived directly from the original assays are shown as a heatmap with positions along CHK2 on the x-axis and amino acid substitutions on the y-axis. Blue indicates deleterious variants with scores near 0, white represents tolerated variants with scores near 1, red indicates apparently 'hyper-complementing' variants with scores above 1, yellow indicates the canonical wild-type amino acid, and grey indicates missing data. Within each cell, the estimated standard error is indicated by the total length of lines as described in the legend (e.g., 0 error is indicated with a dot, error of 1 is indicated by a full-length slash, and an error higher than 1 is indicated by an X). The census track along the top of the plot depicts the distribution of scores at each position.

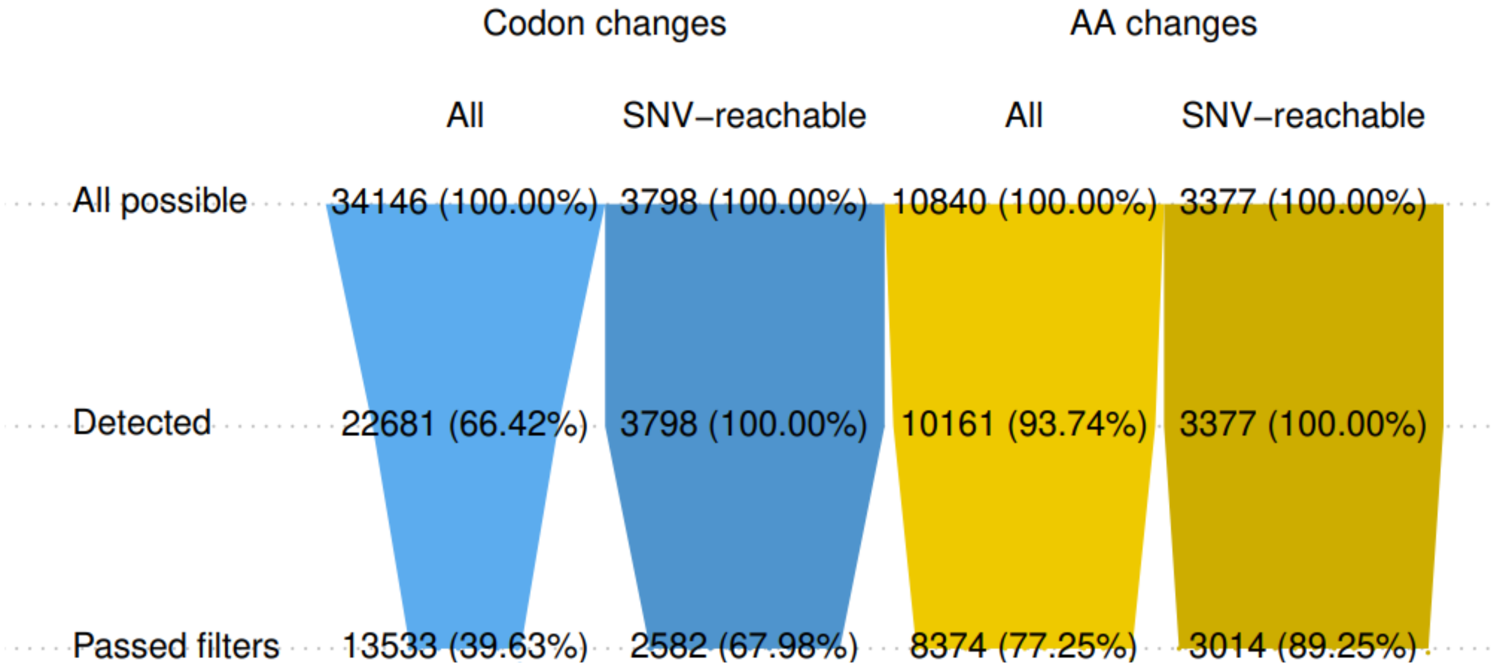

Figure S6. Coverage of all possible codons and amino acids in the CHK2 variant effect map.
The proportion of variants in CHEK2 covered by our experiment is indicated, either as: a fraction of all possible codon-level substitutions (lighter blue), a fraction of codon-level substitutions that can be reached via a single nucleotide change (darker blue); a fraction of all possible amino acid changes (lighter yellow); and a fraction of all possible amino acid changes that can be reached via a single-nucleotide change (darker yellow). “Detected” indicates variants that were observed during sequencing while “passed filters” refers to variants that were sufficiently well represented in the non-select library to be considered well-measured and were included in the final CHEK2 variant effect map.

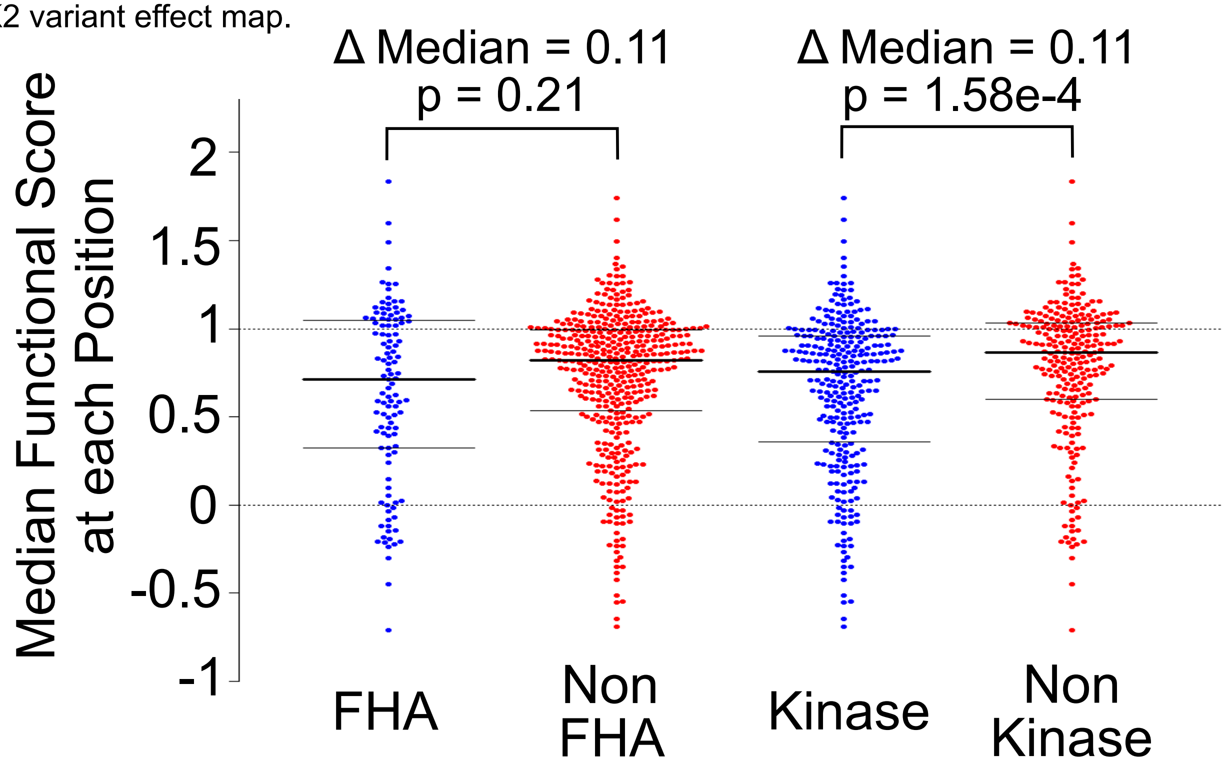

Figure S7. Comparing Functional scores in the FHA and kinase domain to the rest of CHK2.
The median functional score for missense variants at each position were stratified into those located in the FHA domain (92 to 205), the kinase domain (212 to 501), and those not located in the FHA domain (2 to 91 and 206 to 543) or not in the kinase domain (2 to 211 and 502 to 543). Positions located in the FHA domain were compared to those outside the FHA, as well as kinase domain positions compared to non-kinase domain, by Wilcoxon rank-sum test. The median value and 25th and 75th quantiles are overlaid on each distribution as solid horizontal lines. The dashed horizontal lines across all distributions indicate scores of zero and one, indicating deleterious and neutral variants respectively.

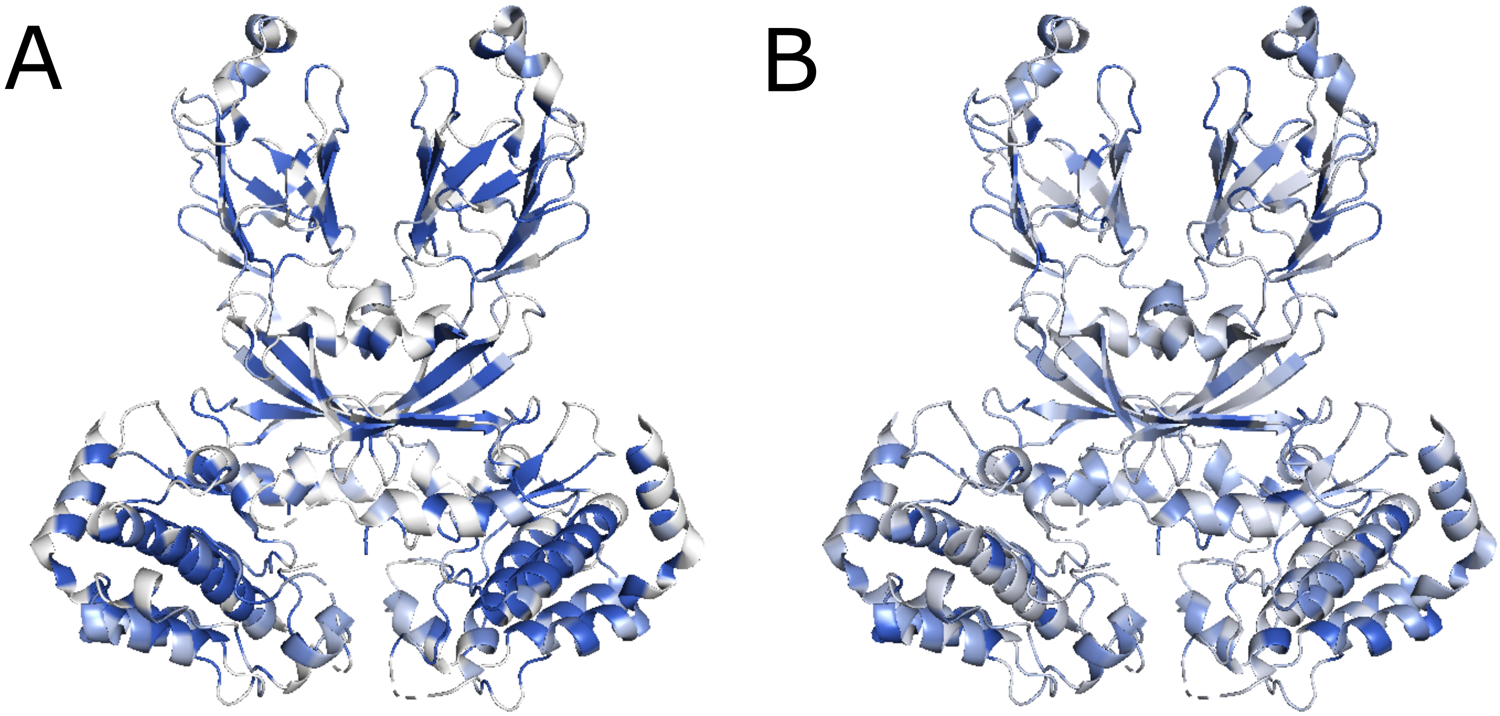

Figure S8. FoldX ΔΔG predicted stability effects overlaid on a homodimeric CHK2 crystal structure. **A** The median ΔΔG score for each position was calculated using FoldX and overlaid on the 3i6u CHK2 crystal structure. Darker blue colours indicate regions of CHK2 with destabilising ΔΔG scores (i.e. ΔΔG greater than 2), light blue indicates regions that were somewhat destabilising (i.e. ΔΔG less than 2 but greater than 0.5), and grey colouration indicates neutral effects i.e. ΔΔG less than 0.5 but greater than -0.5). **B** The difference in medians between FoldX-derived ΔΔG and our functional scores were overlaid on the 3i6u CHK2 crystal structure. FoldX and functional score were transformed to have a maximum value of 1 and minimum value of 0, with the absolute difference calculated. Regions that differed between ΔΔG and functional score are depicted in shades of blue, while areas coloured in white indicate agreement between ΔΔG and functional score (i.e. destabilising regions by ΔΔG are damaging by functional score, or stable regions of ΔΔG are tolerated according to functional score).

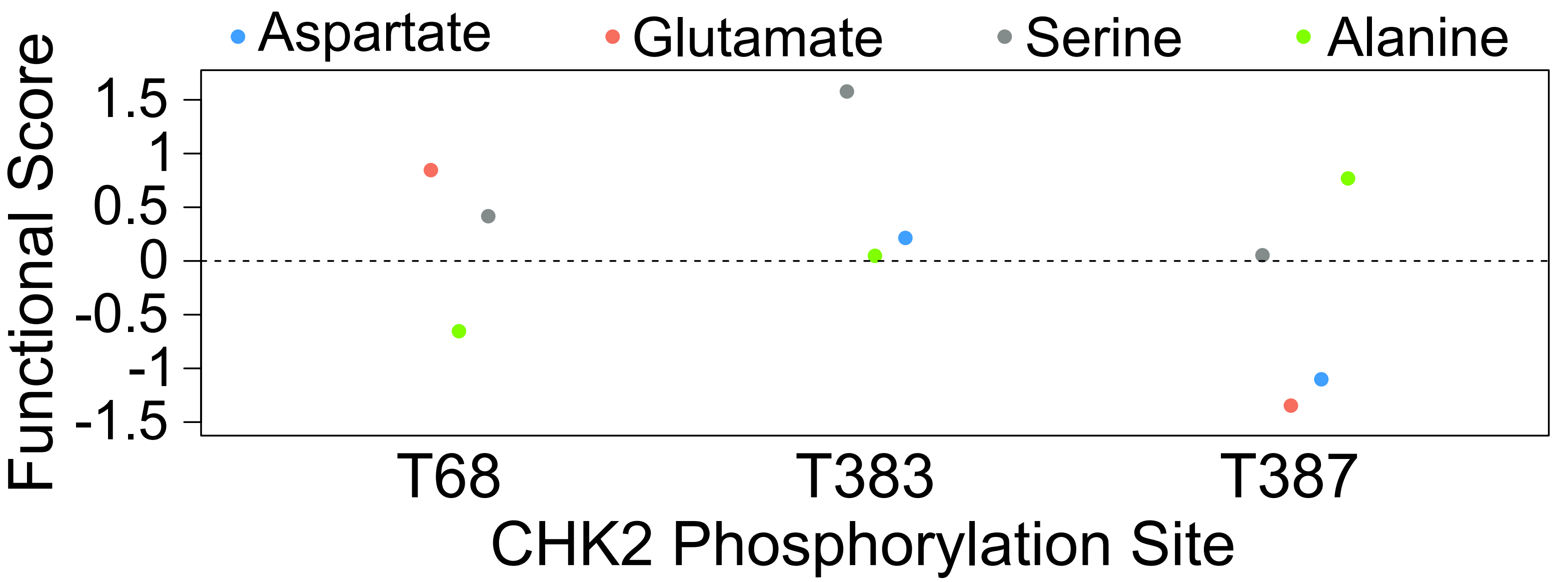

Figure S9. Effect of phosphomimetic and phosphodead variants at known CHK2 phosphorylation sites. Functional scores are shown for individual variants located at phosphorylation sites critical for CHK2 activation. Phosphomimetic mutations, aspartate and glutamate, are shown in blue and red respectively, the purportedly neutral variant (due to its ability to be phosphorylated) serine is shown in grey, and phosphodead alanine shown in green.

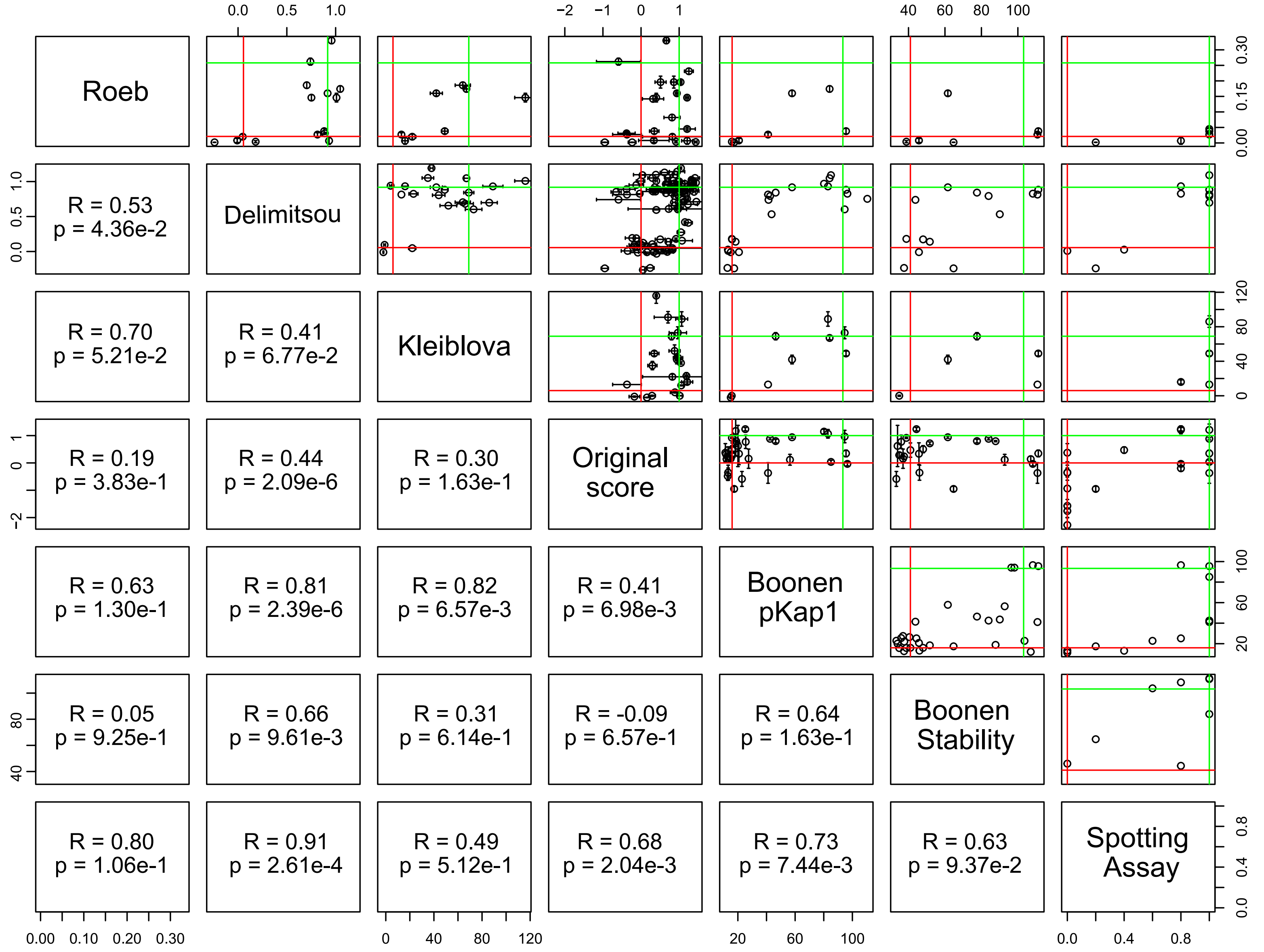

Figure S10. Scatterplots comparing all functional assays.

Scatterplots were generated comparing each of the functional assay datasets indicated on the diagonal axis. Starting from the top-left, this includes Roeb et al. 2012, Delimitsou et al. 2019, Kleiblova et al. 2019, our experimental functional scores, Boonen et al. 2022 pKAP1 and stability scores, and finally MMS spotting assays we performed for individual variants (data not shown). Correlation between datasets was assessed by Pearson correlation coefficient.

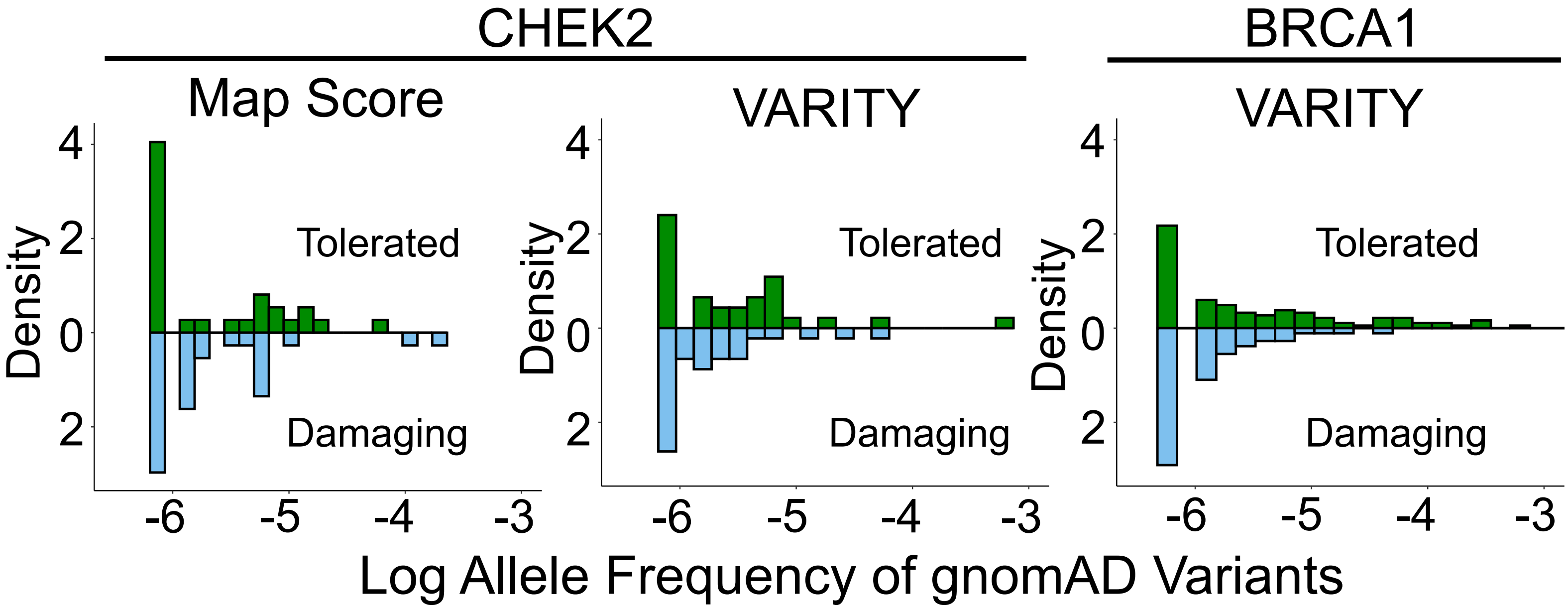

Figure S11. gnomAD allele frequencies for CHEK2 variants does not correlate with functional scores or VARITY. Allele frequencies for all missense variants in CHEK2 or BRCA1 were collected from gnomAD v4.1.0 and matched to either the variant effect map or to VARITY_R. Variants with map or VARITY_R scores in the bottom or top 5th-95th percentiles were isolated and their log10 allele frequencies plotted as a histogram. The top green half of the plot includes the variants expected to be tolerated by map score or VARITY_R, and the bottom blue half of the plot includes variants expected to be damaging variants by map score or VARITY_R.

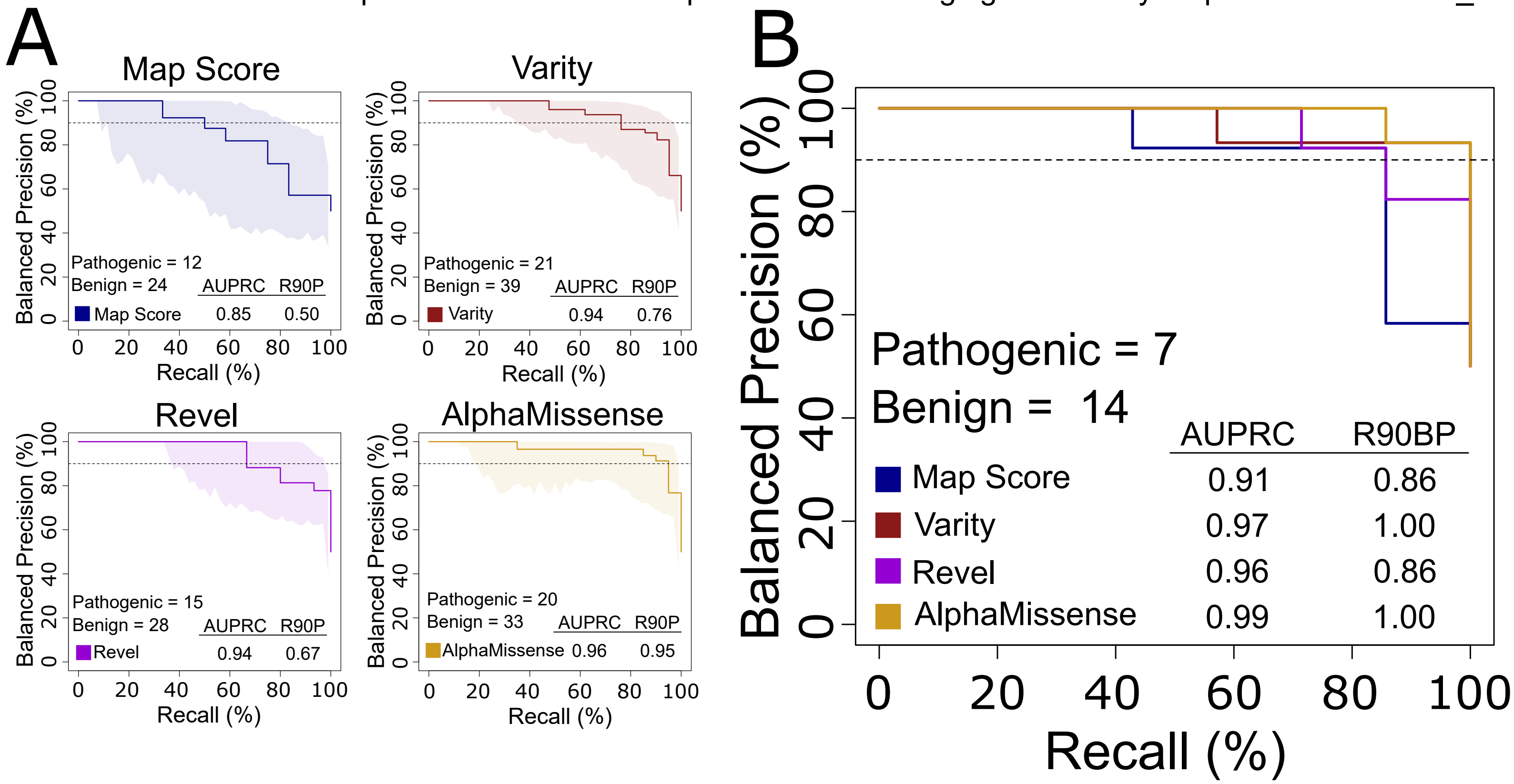

Figure S12. Balanced Precision-Recall Curves of computational and functional scores against variants with known pathogenic or benign annotations. A Using the direct original CHEK2 map scores and a known set of clinically annotated pathogenic or benign CHEK2 variants from Invitae, we evaluated balanced precision—defined at each score threshold by the fraction of variants that are pathogenic given a balanced (50% prior probability of pathogenicity) test set—versus recall (fraction of pathogenic variants captured at this threshold). The horizontal dashed line indicates Recall at 90% Balanced Precision (R90BP) with the numerical Area Under the Precision Recall Curve (AUPRC) and R90BP listed in the bottom-left hand legend. Individual PRCs are shown for functional scores (blue), VARITY_R (red), REVEL (purple), and AlphaMissense (Gold). **B** PRCs were generated for an overlapping set of variants that had scores in all three computational predictors (VARITY_R, REVEL, and AlphaMissense) as well as functional scores in the CHEK2 map.

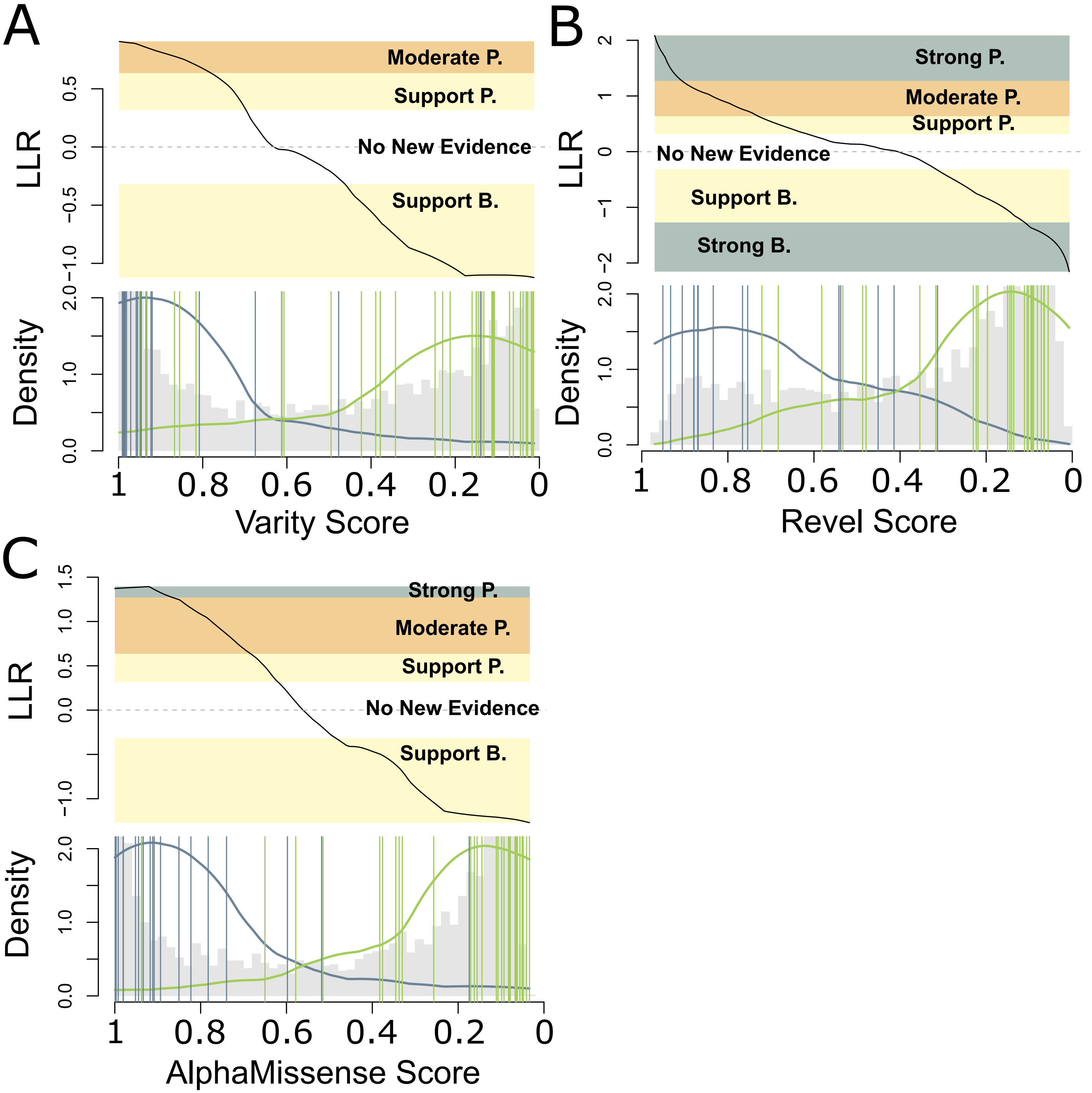

Figure S13. CHEK2 Log-Likelihood Ratios for computational predictors Varity, Revel, and AlphaMissense. LLRs of pathogenicity were calculated as described in Figure 8 panel C and in the methods. The log ratio between likelihood of observing a score in the positive pathogenic reference set (blue) compared to the negative benign reference set (green) was calibrated to ACMG evidence strengths. Probability distributions are overlaid on the grey histogram of CHEK2 missense variant scores with the top panel showing which score ranges correspond to each ACMG evidence strength. Panel **A** shows Varity_R, panel **B** Revel, and panel **C** AlphaMissense.

Region	Amino Acid (AA) Changes Per Clone	Fraction of all possible AA changes that are well measured	Fraction of all possible 1 nt-accessible AA changes that are well measured
1	0.51	86%	99%
2	0.17	81%	100%
3	0.37	92%	100%
4	0.22	62%	98%

Table S1. Fractions of amino acid substitutions that were sufficiently well represented in the “non-select” expression library (after transformation into yeast but not subjected to selection) to be considered well-measured. Here, we considered amino-acid substitutions to be well measured if at least 50 read counts were observed in the non-select library. Fraction of all possible codon changes considers missense and nonsense variants across all CHEK2 positions.

CHK2 amino acid position	Nucleotide and inhibitor binding sites	Median	Mean	Number of variants with score > 0.5	Number of variants with score < 0.5
**G227**	ADP	0.19	0.49	8	9
**S228**	ADP	0.50	0.49	8	8
**G229**	ADP	0.06	0.33	4	9
**G232**	ADP	-0.35	-0.38	0	16
**V234**	ADP and DBQ	-0.39	-0.13	4	13
**A247**	ADP and DBQ	-0.26	-0.26	2	14
**K249**	ADP and DBQ	-0.31	0.08	3	12
**L301**	DBQ	0.23	0.32	6	11
**E302**	ADP and DBQ	1.12	1.23	18	0
**L303**	ADP	1.20	1.37	15	0
**M304**	ADP and DBQ	0.70	0.65	10	7
**E308**	ADP and DBQ	0.04	-0.11	3	14
**E351**	ADP and DBQ	0.22	0.07	4	12
**N352**	ADP and DBQ	0.04	-0.27	0	18
**L354**	ADP and DBQ	-0.22	0.17	6	12
**D368**	ADP	-0.62	-0.38	1	16

Table S2. List of residues that had been previously identified as important for CHK2 activation on the basis of CHK2 crystal structures, based on proximity to either complexed ADP or the competitive ADP inhibitor debromohymenialdisine (DBQ). Columns of median and mean score and the number of variants above or below a score of 0.5 consider all well-measured missense variants at each position.

Hotspot	CHK2 amino acid position	Median	Mean	Number of variants with score > 0.5	Number of variants with score < 0.5
**APE**	392	-0.06	-0.02	5	14
	393	-0.76	-0.56	2	13
	394	-0.34	-0.28	2	12
**Salt bridge E**	273	0.43	0.41	1	2
**VAIK**	246	0.35	0.53	9	10
	247	-0.26	-0.26	2	14
	248	-0.05	0.25	8	10
	249	-0.31	0.08	3	12
**HRD**	345	-0.11	-0.27	2	14
	346	0.05	-0.04	2	15
	347	-0.55	-0.45	2	15
**GxGxxG**	227	0.19	0.49	8	9
	228	0.50	0.49	8	8
	229	0.06	0.33	4	9
	230	1.27	1.12	3	1
	231	1.12	0.98	13	5
	232	-0.35	-0.38	0	16
**DFG**	368	-0.62	-0.38	1	16
	369	-0.42	-0.57	0	18
	370	0.07	0.07	2	6
**HRD + 5**	352 (Asn)	0.04	-0.27	0	18
**APE – 6**	386 (Gly)	0.05	-0.03	5	14
**HRD – 6**	339 (His)	0.86	0.72	10	7
**HRD + 7**	354 (Leu)	-0.22	0.17	6	12
**HRD – 7**	338 (Leu)	0.56	0.49	7	7

Table S3. Mutational Hotspots Mutational hotspots identified in Hudson et al. 2018 were separated according to their correspondence to distinct kinase activation motifs. For each hotspot, the table indicates the amino acid position relative to the beginning (negative values) or end (positive values) of the nearest motif, as well as the median and mean of original functional scores at these positions. Of the 23 CHK2 hotspots identified, all 16 that fell within a named motif (APE, VAIK, HRD, DFG and the three G positions within the GxGxxG motif) scored as intolerant to variation in our map (i.e., with median scores below 0.5).

Tiling primer name	Tiling primer sequence
**CHEK2_1F**	TACACGACGCTCTTCCGATCTCAACTTTGTACAAAAAGCAGGCTGCATG
**CHEK2_1R**	AGACGTGTGCTCTTCCGATCTGTAGAGGAGCTGGATATGCC
**CHEK2_2F**	TACACGACGCTCTTCCGATCTTCCTCCTCACAGTCCCAG
**CHEK2_2R**	AGACGTGTGCTCTTCCGATCTCAGGTTCTTGGTCCTCAGG
**CHEK2_3F**	TACACGACGCTCTTCCGATCTTGTCCACTCAGGAACTCTATTCTATT
**CHEK2_3R**	AGACGTGTGCTCTTCCGATCTCCAAACCAGTAGTTGTCATTCAC
**CHEK2_4F**	TACACGACGCTCTTCCGATCTGATGGATTTGCCAATCTTGAATGT
**CHEK2_4R**	AGACGTGTGCTCTTCCGATCTCCACTTCCCTGAAAATCCGAA
**CHEK2_5F**	TACACGACGCTCTTCCGATCTCCGAACATACAGCAAGAAACAC
**CHEK2_5R**	AGACGTGTGCTCTTCCGATCTATTGTTATTCAAAGGACGGCG
**CHEK2_6F**	TACACGACGCTCTTCCGATCTCAGAGCTTGTAGGGAAAGGAAAA
**CHEK2_6R**	AGACGTGTGCTCTTCCGATCTATGATGTATTCATCTCTTAATGCCTTAG
**CHEK2_7F**	TACACGACGCTCTTCCGATCTCTGACTGTAGATGATCAGTCAGTTTAT
**CHEK2_7R**	AGACGTGTGCTCTTCCGATCTTTTTGCTGATGATCTTTATGGCTACT
**CHEK2_8F**	TACACGACGCTCTTCCGATCTGCTTTCGAGAGGAAAACATGTAAG
**CHEK2_8R**	AGACGTGTGCTCTTCCGATCTTGATGATGCAAGGATGATTTAGCT
**CHEK2_9F**	TACACGACGCTCTTCCGATCTAATGTTGAAACAGAAATAGAAATTTTGAAA
**CHEK2_9R**	AGACGTGTGCTCTTCCGATCTGGCGTTTATTCCCCACCA
**CHEK2_10F**	TACACGACGCTCTTCCGATCTGGGGAGAGCTGTTTGACAAA
**CHEK2_10R**	AGACGTGTGCTCTTCCGATCTGACAGTAAAACATTCTCTGGCTTTAAG
**CHEK2_11F**	TACACGACGCTCTTCCGATCTTTCATGAAAACGGTATTATACACCGT
**CHEK2_11R**	AGACGTGTGCTCTTCCGATCTGGGGTTCCACATAAGGTTCTC
**CHEK2_12F**	TACACGACGCTCTTCCGATCTGATTTTGGGAGAGACCTCTCTC
**CHEK2_12R**	AGACGTGTGCTCTTCCGATCTCCCACTAAGGCAGATAAAAAGAATAAC
**CHEK2_13F**	TACACGACGCTCTTCCGATCTTGGACTGCTGGAGTTTAGGA

Table S4. Tiling primers. For each of the plasmid libraries from non-selective and selective pools, primers carrying a binding site for Illumina sequencing adaptors were used to amplify short template amplicons (tiles) of ~150 bp such that the union of tile positions internal to the priming sites covered the entire ORF.
